# Oxidation State Tuning of Room Temperature Phosphorescence and Delayed Fluorescence in Phenothiazine and Phenothiazine‐5,5‐dioxide Dimers

**DOI:** 10.1002/chem.202300428

**Published:** 2023-04-19

**Authors:** Iain A. Wright, Marc K. Etherington, Andrei S. Batsanov, Andrew P. Monkman, Martin R. Bryce

**Affiliations:** ^1^ Department of Chemistry Durham University South Road Durham DH1 3LE UK; ^2^ School of Chemistry University of Edinburgh David Brewster Road Edinburgh EH9 3FJ UK; ^3^ Department of Physics Durham University South Road Durham DH1 3LE UK; ^4^ Department of Mathematics, Physics and Electrical Engineering Northumbria University Ellison Place Newcastle upon Tyne NE1 8ST UK

**Keywords:** delayed fluorescence, heterocycles, oxidation, phenothiazine, photophysics

## Abstract

Heterocyclic dimers consisting of combinations of butterfly‐shaped phenothiazine (**PTZ**) and its chemically oxidized form phenothiazine‐5,5‐dioxide (**PTZ(SO_2_)**) have been synthesized. A twist is imposed across the dimers by *ortho*‐substituents including methyl ethers, sulfides and sulfones. X‐ray crystallography, cyclic voltammetry and optical spectroscopy, underpinned by computational studies, have been employed to study the interplay between the oxidation state, conformational restriction, and emission mechanisms including thermally activated delayed fluorescence (TADF) and room temperature phosphorescence (RTP). While the **PTZ(SO_2_)** dimers are simple fluorophores, the presence of **PTZ** induces triplet‐mediated emission with a mixed **PTZ**‐**PTZ(SO_2_)** dimer displaying concentration dependent hallmarks of both TADF and RTP.

## Graphical abstract

**Relationships** between prompt fluorescence, room temperature phosphorescence and thermally activated delayed fluorescence are established in a series of dimers of phenothiazine and its chemically oxidized congener phenothiazine‐5.5‐dioxide. This provides detailed insights into the interplay between the singlet and triplet manifolds of these heterocycles and emphasizes the complex functional electronic structure of phenothiazines.

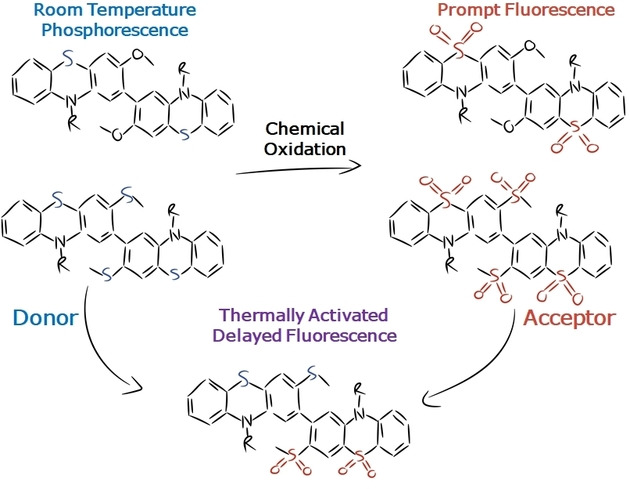

## Introduction

**Figure 1:**
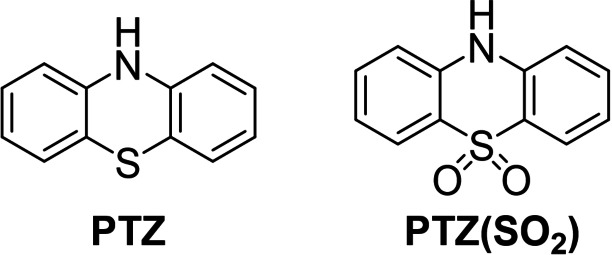
Molecular structures of **PTZ** and **PTZ(SO_2_)**.

10*H*‐phenothiazine (**PTZ**, Figure 1) is a classic heterocyclic motif with a rich history in pharmaceuticals and dyestuffs.[^1^, ^2^, ^3^, ^4^] It is a strong electron‐donor with a total of 16‐π‐electrons due to contributions from the lone pairs of the saturated N and S atoms. However, the break in conjugation between the two benzene rings renders the central 1,4‐thiazine ring non‐aromatic which results in a non‐planar conformation that is bent between the plane of the two benzene rings. This is often referred to as a “butterfly” geometry.

The electrochemical properties of **PTZ** are well‐understood. It is able to undergo a fully reversible single‐electron oxidation at low applied potentials to form stable radical cation species which gives it intriguing electrochemical and magnetic properties.[^5^, ^6^, ^7^, ^8^, ^9^, ^10^] These reliable electrochemical properties of **PTZ** derivatives have led to their study in a variety of organic electronic technologies,^[11]^ most notably organic dye‐sensitized,[^12^, ^13^, ^14^, ^15^, ^16^, ^17^] bulk heterojunction[^18^, ^19^, ^20^, ^21^] and perovskite solar cells.^[22]^

In contrast, the emissive properties of **PTZ** are complex, often involving an interplay between triplet‐mediated luminescence processes and conformational changes associated with the butterfly geometry. **PTZ** derivatives have been studied as the donor component of thermally activated delayed fluorescence (TADF) molecules. It was discovered to exhibit two conformations (*H*‐intra and *H*‐extra) that affect the lone pair of the nitrogen when **PTZ** is bonded to an acceptor moiety. The conformation of any specific **PTZ** has been observed to have significant implications on the TADF mechanism[^23^, ^24^, ^25^, ^26^] and the overall performance of organic light‐emitting diodes (OLEDs) utilising charge‐transfer molecules containing phenothiazine.^[27]^

Chemical oxidation of the bridging sulfide in **PTZ** to 10*H*‐phenothiazine‐5,5‐dioxide (**PTZ(SO_2_)** Figure 1) removes the endocyclic lone pair of the sulfur atom from the π‐cloud and introduces an electron‐withdrawing sulfone group to the molecule. This renders **PTZ(SO_2_)** an electron‐diminished π‐system in comparison with **PTZ**. **PTZ(SO_2_)** retains a butterfly geometry but has a wider angle between the benzene rings. Complementing **PTZ** which acts as a donor, electron‐poor **PTZ(SO_2_)** has shown promise as the acceptor component of TADF emitters.[^28^, ^29^, ^30^] The oxidation state of the sulfur atom provides a further route towards controlling optoelectronic properties for any given application.

Alongside TADF, **PTZ** and **PTZ(SO_2_)** based emitters have also been shown to exhibit a second triplet‐mediated emission mechanism, organic room temperature phosphorescence (RTP).[^31^, ^32^, ^33^] By utilising the triplet manifold, both TADF and RTP can circumvent traditional loss mechanisms and achieve an internal quantum efficiency of 100 % in OLEDs.

The question arises of what optoelectronic interplay might be expected between these two moieties. Covalently linking **PTZ** and **PTZ(SO_2_)** together will produce a D‐A structure in line with TADF design rules and both **PTZ** and **PTZ(SO_2_)** are established RTP emitters. Which emission mechanism will dominate, or will aspects of both be observed?

Furthermore, in recent work we demonstrated how sensitive the photophysical properties of dimers of ring‐fused *N*‐ and *S*‐heterocycles including carbazole, dibenzothiophene‐ and thioxanthene‐*S*,*S*‐dioxide are to the molecular conformation.[^34^, ^35^] If a large dihedral angle is enforced between adjacent heterocycles by introducing steric hindrance, the energy of the triplet state (*E*_T_) of the molecule is increased. This is observed experimentally when *E*_T_ is measured in dilute thin films of the dimers. However, packing forces in the supramolecular environment of a neat thin film can flatten the molecules and reduce the dihedral angles or inhibit any molecular motion required to enter an excited state. This suppresses the *E*_T_ and turns an objectively useful molecule (in dilute film) into a material of more limited practical utility in an OLED. Given the oxidation of **PTZ** to **PTZ(SO_2_)** results in significant changes to the fold angle of the molecule, this conformational change should also impact on the concentration dependent modulation of *E*_T_ and other excited state properties.

In existing studies of **PTZ** dimers and short chain length oligomers,[^36^, ^37^, ^38^, ^39^, ^40^, ^41^, ^42^, ^43^, ^44^, ^45^, ^46^] we are aware of only three reports which feature functional groups bound *ortho*‐ to a **PTZ**‐**PTZ** bond. Two of these[^47^, ^48^] report this motif as a consequence of photodegradation of the drug molecules methopromazine and levomepromazine, while the third^[6]^ is the only report of *ortho*‐substituted functional groups being used specifically to restrict inter‐cyclic rotation of a **PTZ**‐dimer. At time of writing no studies of **PTZ(SO_2_)** dimers could be found.

**Figure 2:**
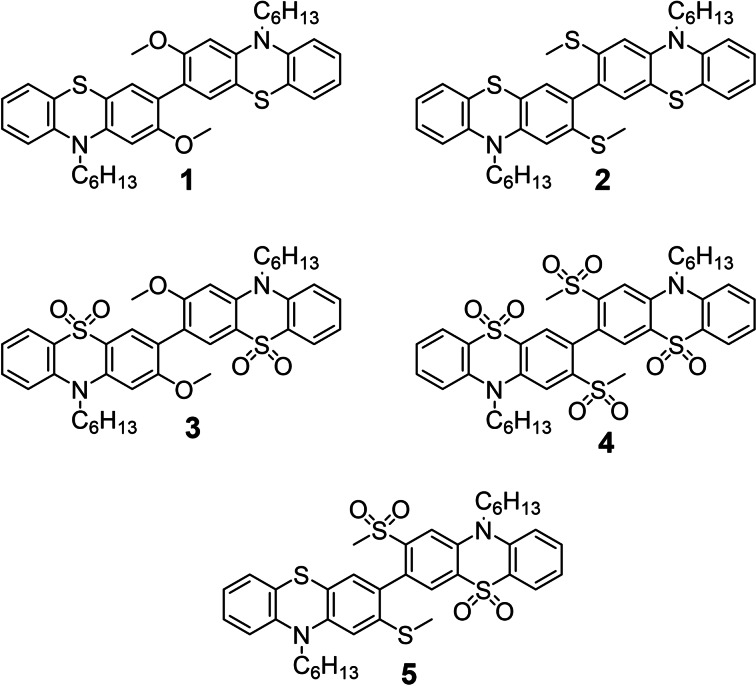
Molecular structures of electron‐rich **PTZ** D‐D dimers **1** and **2**, electron‐poor **PTZ(SO_2_)** A‐A dimers **3** and **4** and mixed **PTZ**‐**PTZ(SO_2_)** D‐A dimer **5**.

The synthesis and study of twisted dimers of **PTZ**, **PTZ(SO_2_)** and a mixed **PTZ‐PTZ(SO_2_)** dimer are therefore presented here with a view to understanding any interplay between: i) the conformation of the inter‐cyclic bond; ii) whether the phenothiazines adopt the *H*‐intra or *H*‐extra conformation; and iii) the oxidation state of sulfur atoms in the dimers on triplet mediated luminescence mechanisms. **PTZ** heterocycles are bound directly together with sterically incumbent methoxy **1** or methylsulfanyl **2** groups *ortho*‐ to the central inter‐cyclic bond (see Figure 2) to make D‐D dimers. Simple chemical oxidation of the sulfur atoms to sulfones provides the electron deficient A‐A **PTZ(SO_2_)** dimers **3** and **4** and a cross‐coupling approach produces the novel **PTZ‐PTZ(SO_2_)** D‐A compound **5**.

This concise series of compounds allows us to gain a deeper understanding of the photophysical properties of these donor and acceptor moieties, and provides routes to control the layout and behaviour of the excited state of phenothiazine‐based materials through both structural and electronic tuning. By producing the **PTZ(SO_2_)** acceptor molecules directly from the **PTZ** donors, we have also established an efficient single synthetic approach to produce structural diversity rapidly and economically.

## Results and Discussion

### Synthesis

**Scheme 1:**
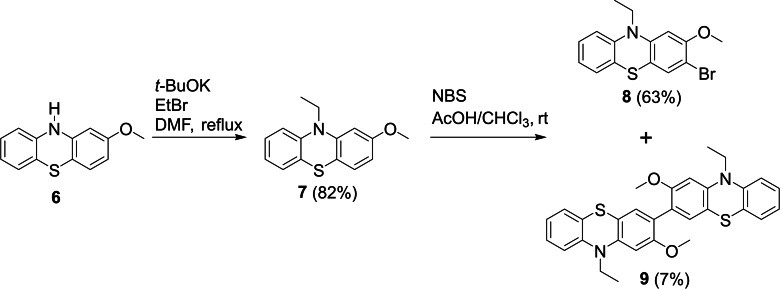
Initial synthetic approach and serendipitous synthesis of D‐D dimer **9**.

In our initial studies, alkylation of commercially available 2‐methoxy‐10*H*‐phenothiazine **6** was achieved by deprotonation with potassium *tert*‐butoxide prior to addition of ethylbromide and heating overnight to give **7** in a good yield (Scheme 1). Subsequent electrophilic bromination with *N*‐bromosuccinimide (NBS) in a mixture of equal volumes of acetic acid and chloroform gave the desired 3‐bromo‐10‐ethyl‐2‐methoxy‐10*H*‐phenothiazine **8** in 63 % yield after purification by column chromatography. During this purification step a second, highly emissive, band was observed on the column displaying a much lower Rf value than **8** and was also collected. Pale yellow/green needles crystallized spontaneously from these column fractions. NMR spectroscopy and mass spectrometry indicated that this emissive band was the desired final product 10,10’‐diethyl‐2,2’dimethoxy‐3,3’‐bi‐10*H*‐phenothiazine **9** which had formed serendipitously, albeit in low yield (7 %). This was subsequently confirmed by X‐ray crystallographic analysis.

Phenothiazines are known to be susceptible to oxidative dimerization and the isolation of **9** from this reaction is concordant with previous studies on the bromination of 10‐phenylphenothiazine.^[49]^ Unfortunately this oxidative dimerization of **7** does not appear to be a favourable reaction as attempts to perform it intentionally with FeCl_3_ as the oxidant in chloroform only yielded unreacted starting material.

**Scheme 2:**
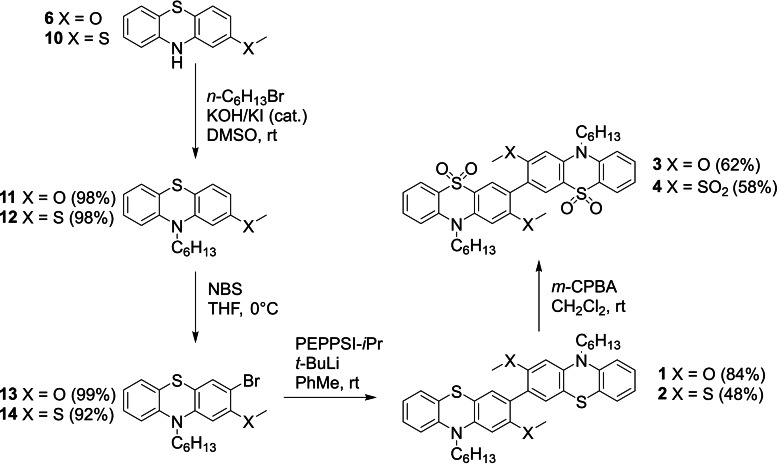
Synthesis of D‐D dimers **1** and **2** followed by oxidation to A‐A dimers **3** and **4**.

Pure **9** proved to be poorly soluble in most common organic solvents, therefore by attaching longer alkyl chains to the nitrogen atom of the phenothiazines we aimed to improve the mechanical properties for solution‐based analysis and processing. The synthetic route was revised and optimized utilising *n*‐hexyl chains as shown in Scheme 2.

**Scheme 3:**
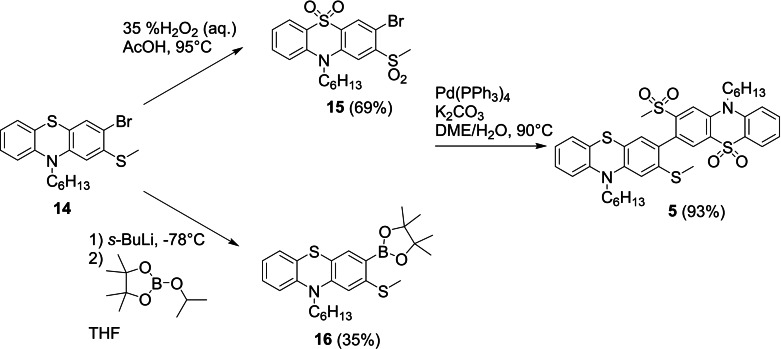
Synthesis of D‐A dimer **5** by Suzuki‐Miyaura coupling.

Both **6** and 2‐(methylsulfanyl)‐10*H*‐phenothiazine **10** were alkylated with *n*‐hexylbromide according to the procedure of Marszalek et al.^[50]^ to give **11** and **12** in near quantitative yields prior to straightforward and selective bromination with NBS in THF at reduced temperature to provide key intermediates **13** and **14** in excellent yields. Palladium‐catalysed *tert*‐butyllithium mediated homocoupling proved very effective in obtaining the target dimers **1** and **2**.^[51]^ The drop in yield when moving from *o*‐methoxy to *o*‐methylsulfanyl substituents can be largely attributed to losses incurred during recrystallization of **2** which proved quite soluble in non‐polar solvents. Prior to recrystallization the yield of **2** was comparable to that of **1**. Oxidation with *meta*‐chloroperbenzoic acid (*m*‐CPBA) in methylene chloride at room temperature provided a straightforward approach to the electron poor dimers **3** and **4**. Finally, to obtain the donor‐acceptor dimer **5** (Scheme 3) intermediate **14** presented a convenient starting point as it is easily synthesized in multigram quantities. Oxidation to **15** was achieved with aqueous 35 % hydrogen peroxide in acetic acid while lithium halogen exchange followed by trapping with 2‐isopropoxy‐4,4,5,5‐tetramethyl‐1,3,2‐borolane provided boronic ester **16** which was isolated as an oil. NMR analysis indicated that this oil was a mixture of **16** and the dehalogenated precursor **12** in an approximate ratio of 2 : 1. Owing to the relatively low yield of **16** and the tendency of **12** and **16** to coelute in a range of solvents, this mixture was used in the next step. Suzuki coupling of **15** and **16** in a mixture of 1,2‐dimethoxyethane (DME) and water provided **5** in excellent yield which was purified in a similar fashion to **1**–**4**.

### X‐ray crystallography

**Figure 3:**
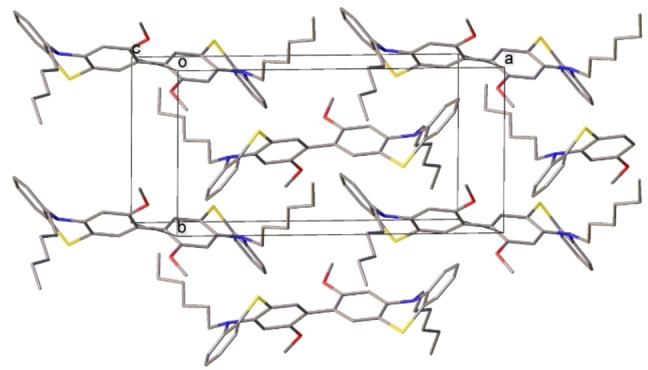
Crystal packing of **1**. All arene‐arene contacts are edge to face (interplanar angle 63°).

**Figure 4:**
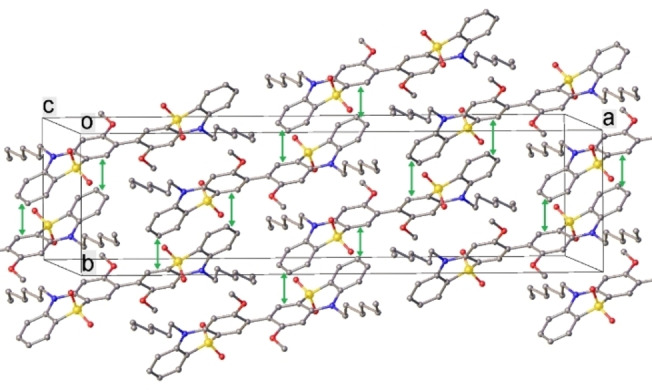
Crystal packing of **3**. Green vectors indicate π‐π stacking contacts (arene/arene interplanar angles 19–20°, mean interplanar separations 3.4 Å, shortest C…C contacts 3.32‐3.35 Å).

**Figure 5:**
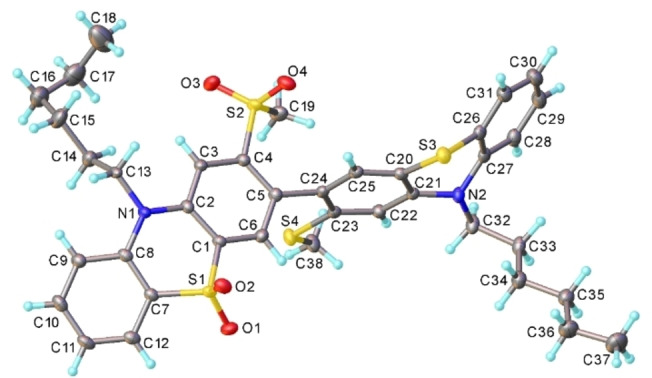
X‐ray molecular structure of **5**.

**Figure 6:**
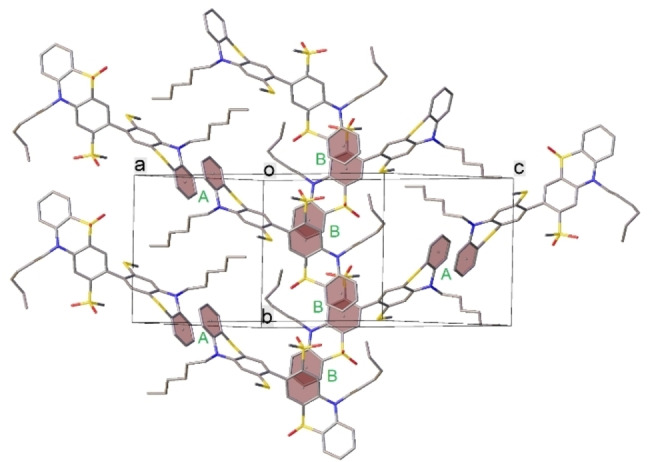
Crystal packing of **5**; π‐π stacked rings are highlighted. Contacts A: arene rings are inversion‐related and rigorously parallel, interplanar separation 3.50 Å; contacts B: interplanar angle 17°, mean separation 3.63 Å, shortest C…C contacts 3.39 Å.

X‐ray molecular structures were obtained for compounds **1**–**5** and **9** (Figures 3, 4, 5, 6, S4.1–S4.6). Molecules **1** and **9** have crystallographic *C*_2_ symmetry, others occupy general positions. **3** crystallizes as a di‐solvate with deuterochloroform. The *n*‐hexyl side‐chains are ordered in **1**, **5** and **9** but intensely disordered in **2**, **3** and **4**. This disorder may be relevant to the fact that the last three materials all undergo reversible phase transitions on cooling, below 160 K (**2**), 200 K (**3**) and between 220 and 200 K (**4**), although the low‐temperature polymorphs were not characterized due to twinning and/or incommensurate modulation emerging during the transition.

All molecules are twisted around the central C−C bond, enough to prevent π‐π conjugation, while the **PTZ** systems are folded along the S…N vectors. Both the twist (τ) and folding (θ) angles vary widely (Table 1), whether between or (for θ) within molecules. The deviation, δ, of the N atom from the plane of the three adjacent C atoms and the intra‐**PTZ** bond distances N−C broadly correlate with θ.

**Table 1:** Molecular geometry from X‐ray structures.

	τ [°]	θ [°]	N−C(**PTZ**) [Å]	δ [Å]
**1**	61.8	51.0	1.417(2)	0.24
**2**	77.1	39.0, 28.2	1.416(4)	0.14
**3**	60.3	20.1, 19.2	1.398(3)	0.04
**4**	84.8	17.7, 30.7	1.395(3)	0.01, 0.05
**5**	79.4	22.7 (SO2), 40.1 (S)	1.400(3), 1.416(3)	0.07, 0.21
**9**	51.5	24.2	1.416(2)	0.18

The crystal packing of **1**, **2** and **9** does not show any π‐π interactions: **PTZ** moieties are surrounded by *n*‐hexyl sidechains (which in **1** and **2** fill the **PTZ** fold), methoxy groups and arene rings (contacting edge‐to‐face) of adjacent molecules. On the contrary, in the crystal of **3**, both **PTZ** moieties of the molecule participate in (symmetrically independent) infinite slipped stacks. Crystal packing of **1** and **3** are shown in Figures 3 and 4 and Figure S4.7.

In the crystal of donor‐acceptor compound **5** (Figure 5), three arene rings of the molecule form stacking interactions with different adjacent molecules (Figure 6), thus also creating a continuous network. Structure **4** is dominated by relatively strong C−H…O hydrogen bonds; polar substituents hinder efficient **PTZ** stacking. Nevertheless, each molecule participates in (rather awkward) π‐π contacts with two others (arene/arene angle 21°, closest C…C separation 4.10 Å). Crystal data and experimental details are included in the Supporting Information (Table S1).

### Electrochemistry

**Figure 7:**
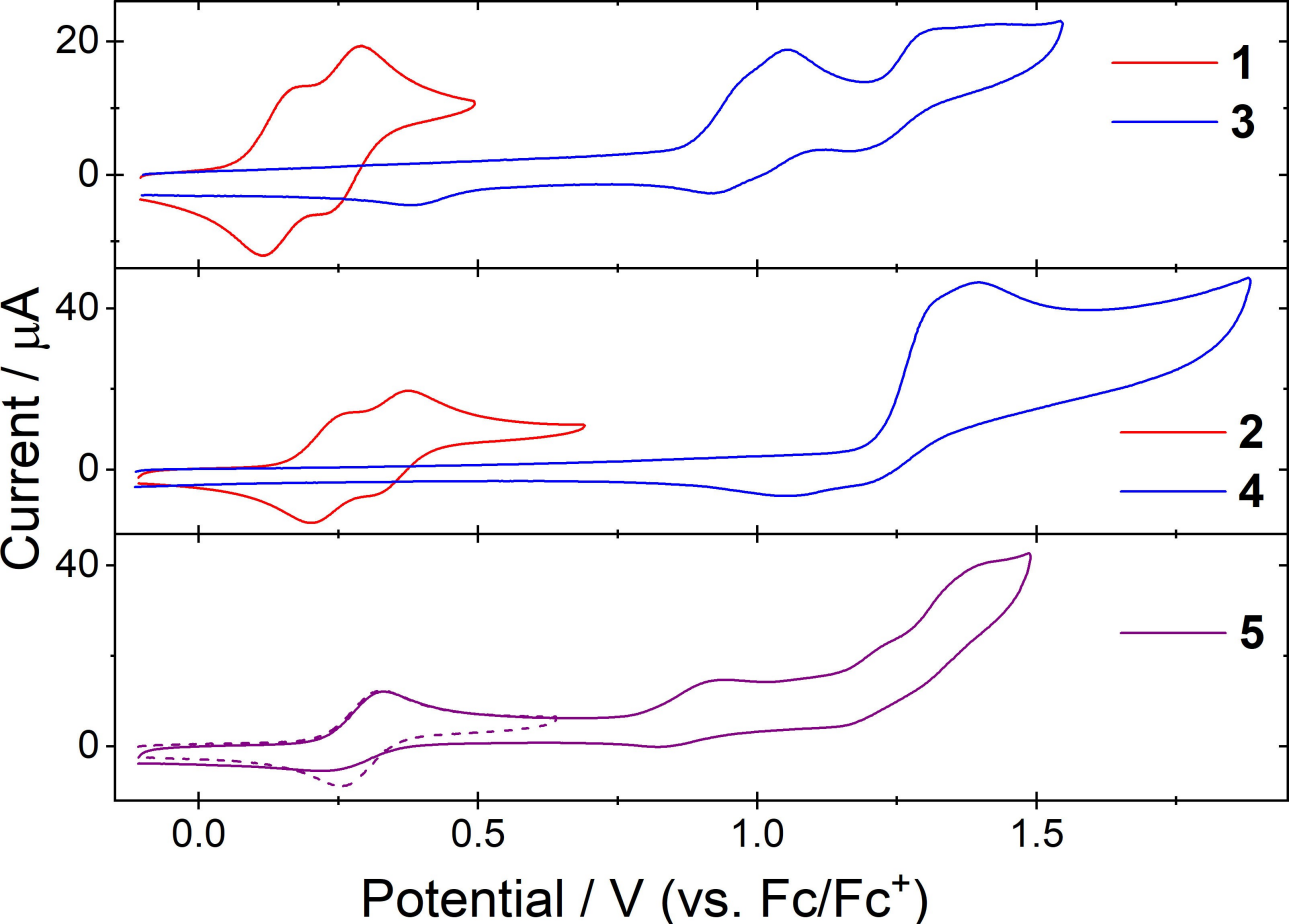
Cyclic voltammetry of **1**–**5**.

The results of cyclic voltammetry (CV) measurements of the dimers **1**–**5** are shown in Figure 7 and summarized in Table 2. To identify their electron donating or accepting ability highest occupied molecular orbital (HOMO) levels were estimated by measuring the onset of the first oxidation potential relative to the half‐wave potential of ferrocene. Only compound **4** displayed a reduction process within the accessible solvent window, therefore, the lowest unoccupied molecular orbital (LUMO) energies were estimated by addition of the optical HOMO‐LUMO gaps calculated from the absorption spectra.

**Table 2:** Electrochemical properties of **1**–**5**.

	*E* ^ox1^ [V]^[a]^	*E* ^ox2^ [V]^[a]^	*E* ^ox3^ [V]^[a]^	*E* ^ox4^ [V]^[a]^	*E* ^red1^ [V]^[a]^	HOMO [V]^[b]^
**1**	+0.15	+0.26	+0.93/0.78^qr^	+1.20^ir^	−2.31^ir^	−5.15
**2**	+0.23	+0.34	+0.98/0.84^qr^	+1.28	–	−5.21
**3**	+0.96^qr^	+1.02^qr^	+1.32/1.17^qr^	–	–	−5.93
**4**	+1.40^ir^	–	–	–	−2.12^ir^	−6.30
**5**	+0.33/ 0.26	+0.88/0.78^qr^	+1.03^ir^	+1.40^ir^	−2.21^ir^	−5.26

[a] CV data were obtained from solutions of 0.1 mmol of the compound, 0.1 M *n*‐Bu_4_NBF_4_ in 4 : 1 CH_3_CN:CH_2_Cl_2_, scan rate 100 mV/s, using a glassy carbon disc working electrode, a non‐aqueous Ag/AgNO_3_ reference electrode and a platinum wire counter electrode. Potentials are quoted versus F_c_^+^/F_c_ couple which was used as internal reference. Reversible oxidations are quoted as *E*_half_ otherwise peak potentials are provided. [b] *E*_HOMO_=−(*E*_onset,ox_ vs F_c_^+^/F_c_+vacuum level) eV. Vacuum level taken as 5.10 eV.^[52]^ ^qr^ Quasi‐reversible peak. ^ir^ Irreversible peak.

The **PTZ** dimers **1** and **3** displayed two overlapping single‐electron oxidations at low oxidation potentials which correspond to sequential radical cation formation on the adjacent phenothiazine heterocycles. Both peaks are fully reversible displaying peak separations close to the 59 mV theoretical ideal and a linear dependence between scan rate and peak current (Figures S5.1 and S5.2). The molecules are easily oxidized because of strong electron donation from the MeO‐/MeS‐groups into the ring. The fact that the oxidation of each phenothiazine is observed sequentially rather than simultaneously indicates that the two phenothiazine rings remain electronically coupled despite the presence of the methoxy groups.[^36^, ^37^, ^38^, ^40^, ^41^, ^42^, ^46^] The separation between the first and second oxidation peaks is *ca*. 50 mV narrower than that of simple 3,3’‐bis(*N*‐alkylphenothiazine) dimers[^36^, ^42^] which demonstrates that the sterically opposed substituents act to reduce delocalization across both heterocycles. Further oxidation processes to higher cationic states are observed (see Supporting Information, Figure S5.3) but these are irreversible.

**PTZ(SO_2_)** dimers **2** and **4** have significantly higher first oxidation potentials and reduced reversibility when compared to the precursors **1** and **3**. This indicates that for the sulfone derivatives, reversible oxidation to a radical cationic state is no longer possible. The absence of any single‐electron reductions down to −2.00 V also proves that the sulfonyl groups do not induce formal electron accepting behaviour and instead act simply as electron‐withdrawing groups resulting in increased first oxidation potentials.

In the mixed D‐A compound **5** the electrochemical characteristics of both the **PTZ** and **PTZ(SO_2_)** heterocycles are clearly evident. A low potential reversible oxidation at +0.30 V is followed by a quasi‐reversible oxidation at +0.83 V, then by two irreversible oxidations at >+1.00 V. The results for **5**, when compared to those of the donor and acceptor dimers **2** and **4**, leads us to assign the first two oxidations as occurring over the donor side of the molecule and the latter two as occurring over the acceptor.

### Steady state computational analysis

**Figure 8:**
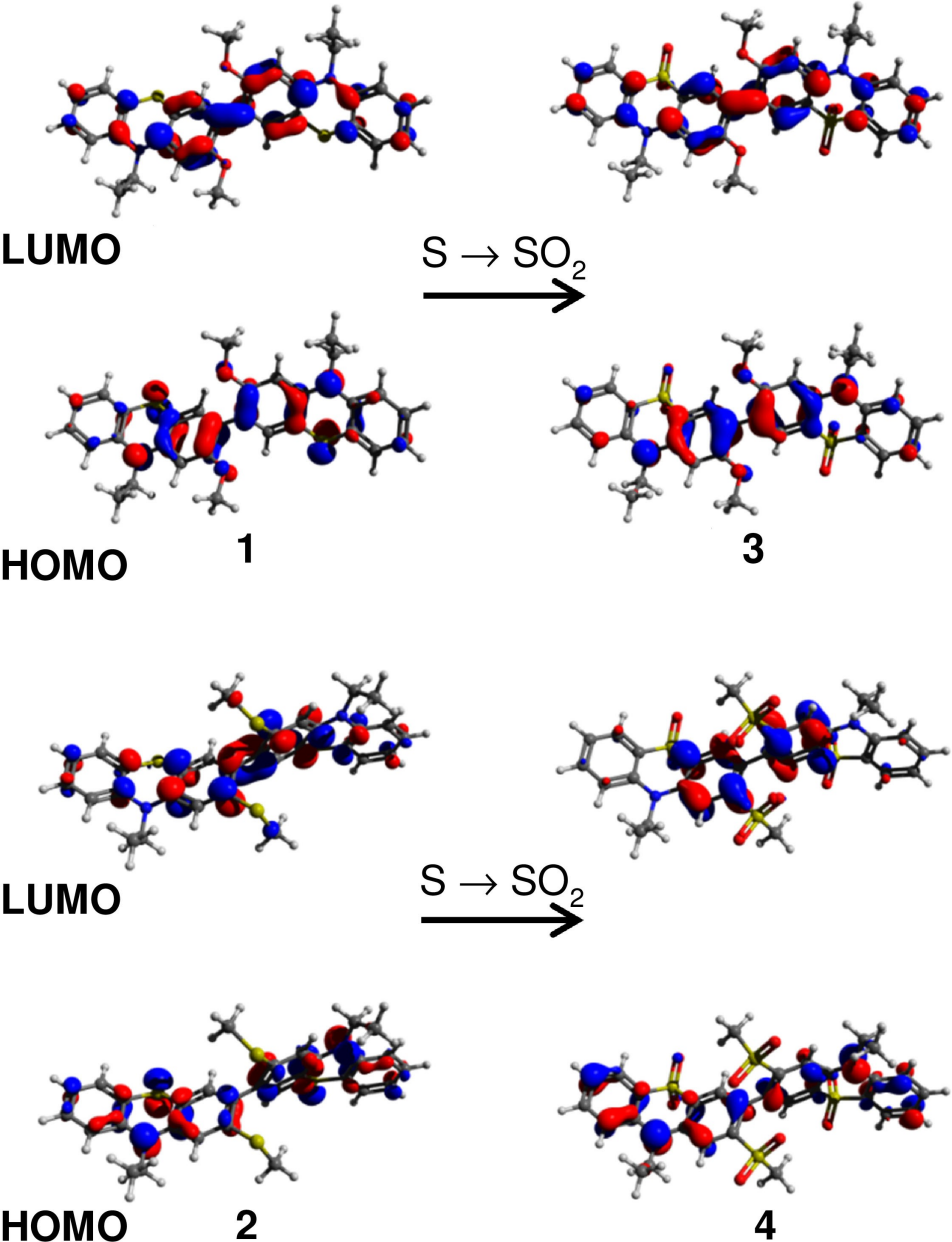
Optimized structures and HOMO and LUMO distributions for compounds **1** and **3** (upper) and **2** and **4** (lower) highlighting the changes in MO profile upon oxidizing S to SO_2_ which converts the D‐D dimers to A‐A dimers.

**Figure 9:**
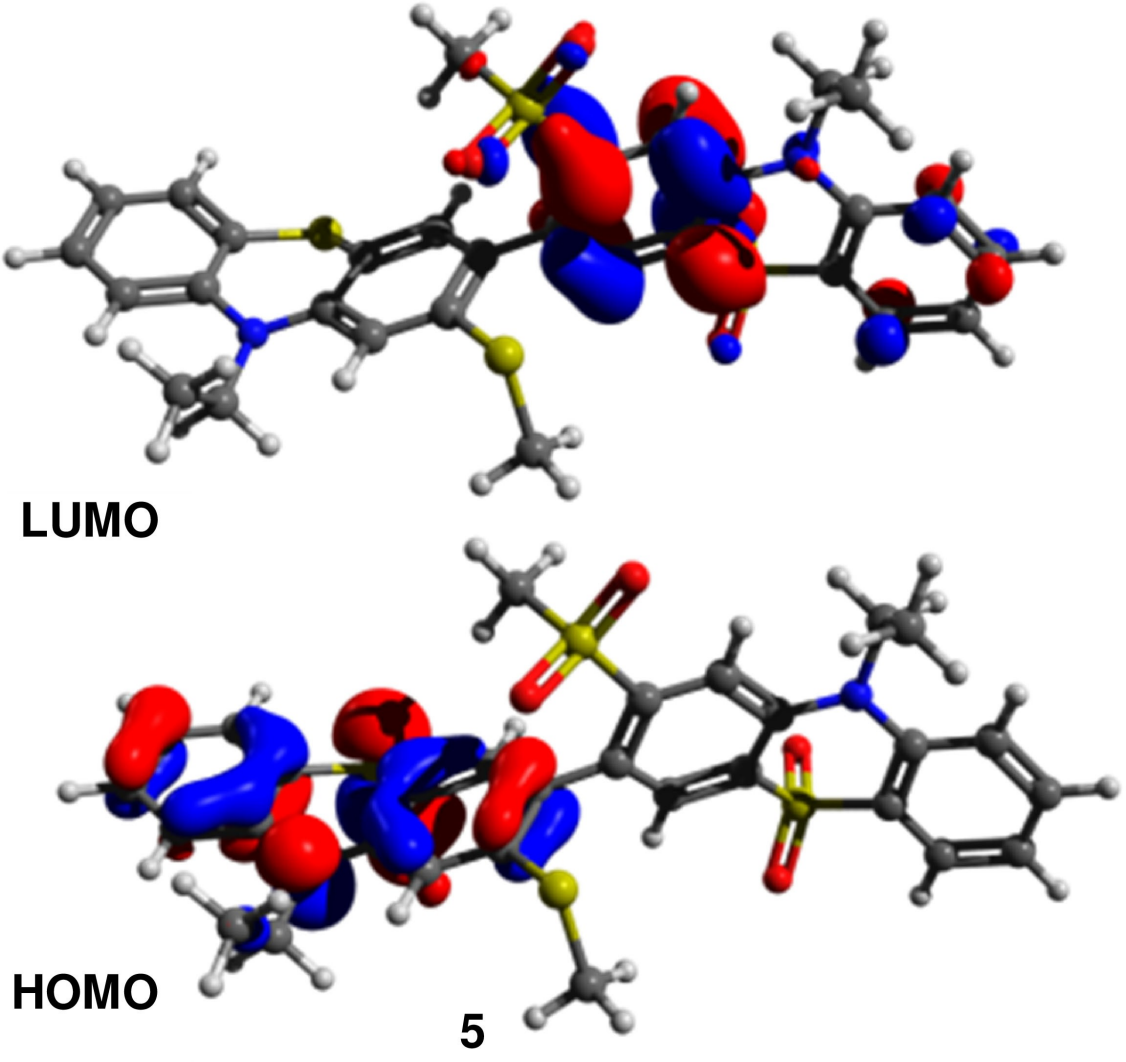
Optimized structures and HOMO and LUMO distributions for D‐A dimer **5** showing the HOMO over the **PTZ** and the LUMO over the **PTZ(SO_2_)**.

Density functional theory (DFT) calculations were performed with ORCA^[53]^ v4.1.1 (**4** and **5**) using the B3LYP hybrid functional and 6‐31G** basis set.[^54^, ^55^, ^56^] Ground state structural optimizations were performed prior to frequency and frontier orbital calculations. Visualization was achieved with Avogadro v1.1.1.^[57]^ at an isosurface value of 0.04. Ethyl rather than hexyl chains were appended to the nitrogen atoms of the phenothiazines in order to reduce the computational cost (N.B. this renders compounds **1** and **9** identical). Calculations were also performed on the simple non‐functionalized dimer 3,3’‐bis(10‐ethyl‐phenothiazine) **bisPTZ** (HOMO and LUMO shown in Figure S6.1) as a model compound to permit comparison with the sterically hindered dimers **1**–**5**. Results are shown in Figures 8 and 9, and Tables 3 and 4.

**Table 3:** Calculated structural properties of **1**–**5** and model compound **bisPTZ** in the ground and first excited singlet state.

	S_0_			S_1_		
	∠_C2C3C3’C2’_ [°]	τ [°]	θ [°]	∠_C2C3C3’C2’_ [°]	τ [°]	θ [°]
**1/9**	131.7	50.74	26.06, 26.03	146.1	35.77	15.73, 15.95
**2**	74.2	70.65	26.20, 26.15	50.1	49.35	24.80, 12.21
**3**	135.5	46.52	25.26	150.2	33.00	23.39, 19.15
**4**	93.2	88.64	26.92	98.4	85.04	23.91, 26.53
**5**	83.8	79.46	26.58(SO2), 26.15(S)	99.7	86.37	23.49(SO2), 10.74(S)
**bisPTZ**	145.5	35.42	25.74, 25.82	160.2	20.55	10.94, 23.80

**Table 4:** DFT and TDDFT calculated properties of **1**–**5** and model compound **bisPTZ**.

	HOMO [eV]	LUMO [eV]	*E*g [eV]	*E*_S1_ [eV]	*f*_osc_	*E*_T1_ [eV]	*E*_ST_ [eV]	SOCME [cm^−1^]
**1/9**	−4.385	−0.486	3.899	3.335	0.41	2.794	0.541	15.69
**2**	−4.762	−0.687	4.075	3.418	0.08	2.899	0.519	8.06
**3**	−5.285	−0.988	4.297	3.841	1.18	3.133	0.708	0.21
**4**	−6.080	−1.810	4.270	3.782	0.11	3.181	0.601	3.19
**5**	−5.025	−1.542	3.483	3.040	0.025	2.906	0.135	4.80
**bisPTZ**	−4.593	−0.747	3.846	3.316	0.50	2.747	0.569	14.11

Certain trends in the calculated structures are immediately obvious. Firstly, considering the overall conformation of the dimers, in comparison with the non‐substituted **bisPTZ** the MeO‐ groups in compounds **1** and **3** have a much more limited effect on τ than the larger MeS and MeSO_2_‐ groups. **1** and **3** are respectively only 13.8° and 10° more twisted than **bisPTZ**. In contrast **2** is significantly twisted at 74.2° and in the MeSO_2_‐ bearing compounds **4** and **5** the heterocycles sit almost completely orthogonal. The oxygen atoms of the MeO‐ groups lie well within H‐bonding distance to the hydrogen on the 1‐position of the adjacent **PTZ** (2.563 Å for **1** and 2.489 Å for **3**) while the MeS‐ groups are at 4.360 Å and the nearest point of contact with the MeSO_2_‐ groups is 3.922 Å between the S and the 1‐hydrogen so a combination of stronger intramolecular forces and steric hindrance explains the differences. These are gas phase calculations and upon comparison with the crystal structures it becomes clear that **1**/**9** and **3** are much more twisted in the crystal phase which indicates that crystal packing interactions are strong enough to overcome these intramolecular forces. The calculated intercyclic dihedral angles for the **PTZ(SO_2_)** containing dimers are broadly consistent with those obtained crystallographically.

Secondly, looking at the fold angle of the **PTZ** and **PTZ(SO_2_)** moieties of the dimers. Major variations in θ were observed in the crystal structures, with the **PTZ(SO_2_)** heterocycles generally assuming a less folded conformation. In contrast, the calculated structures display minimal variation after chemical oxidation of **PTZ** to **PTZ(SO_2_)**. This serves to highlight an interesting difference between chemical oxidation and electrochemical oxidation. Under electrochemical oxidation to a radical cation **PTZ** becomes significantly more planar as is clearly observed in the crystal structures of its charge transfer salts.[^8^, ^10^, ^58^] This effect was replicated in the DFT optimized structures of radical cations (θ^RC^) calculated for the **PTZ** containing dimers **1**, **2** and **5** (Table S6.1 and Figure S6.2).

Trends in the calculated position of the HOMO agree well with those obtained using cyclic voltammetry. The HOMO energy decreases steadily from **1** to **4** while the HOMO of **5** is intermediate to that of **2** and **3**. The calculated LUMO energies also decrease steadily with *E*_g_ increasing from **1** to **4**. The LUMO of **5** sits intermediate to those of the **PTZ(SO_2_)** dimers **3** and **4**. The *E*_g_ for **5** is the narrowest due to its donor‐acceptor structure.

Comparing **1** and **3** the distribution of the frontier orbitals changes very little upon oxidation. The HOMO and LUMO reside primarily over the two central aromatic rings with HOMO contributions from the heteroatoms and quinoidal LUMO distributions with significant LUMO density over the intercyclic bond. This indicates that upon excitation some planarization between the rings may be expected. The frontier orbital distribution for **2** is similar to that of **1** but upon oxidation to **4** the HOMO and LUMO reside exclusively over the **PTZ(SO_2_)** rings with no contribution from the intercyclic bond to the LUMO. By comparing **4** with the other electron deficient dimer **2** we attribute this to the large sulfones on the 2‐positions causing a greater degree of conformational restriction alongside their strong electron inductive effect. These factors combine to significantly increase the energy required to assume a quinoidal form.

The donor‐acceptor character of **5** is obvious with the HOMO situated entirely over the non‐oxidized **PTZ** and the LUMO over the **PTZ(SO_2_)** unit (Figure 6).

### Time‐dependent computational analysis

To provide insight into structural changes upon excitation and to help the interpretation of photophysical studies (see below), TDDFT calculations were completed using TD‐B3LYP/6‐31G**.

The geometry of the first excited singlet state (S_1_) of each molecule was optimized. In S_1_ the dihedral angle between the heterocycles planarizes with the *ortho*‐substituents twisting away from each other in all cases due to steric effects. Smaller changes are observed in the MeSO_2_‐substituted compounds **4** and **5** which remain close to orthogonal in both the ground and excited state. This agrees with the steady state calculations which show significant LUMO distribution across the central inter‐cyclic bond for **1**–**3** but none for **4** and **5** (Figures 5 and 6). The fold angle between the planes of the **PTZ** and **PTZ(SO_2_)** moieties generally decreases upon excitation, but there is a noted asymmetry to this in compounds **2** and **5**. In **5** this is easily understood due to its ICT structure. The electron‐donating **PTZ** planarizes by >15° assuming a charge separated, radical cation‐like state, while the electron poor **PTZ(SO_2_)** is only <3° flatter. As such, this asymmetry seems to indicate some ICT character of **2**.

Vertical excitation energies were calculated for the first 10 singlet and triplet states and optimized geometries for the first excited singlet state obtained (Table 4 and Tables S6.2‐6.6). Across the series, the presence of sulfur atoms on the 2‐positions of **2** and **4** results in greatly reduced S_0_→S_1_ oscillator strength, *f*_osc_, compared to both the compounds **1** and **3** which bear methoxy groups and for the non‐substituted dimer **bisPTZ**. Comparing the D‐D (**1** and **2**) and A‐A (**3** and **4**) dimers, the oxidation of **PTZ** to **PTZ(SO_2_)** serves to increase *f*_osc_ albeit to a much larger extent for **1** / **3** versus **2** / **4**. Chemical oxidation also serves to increase *E*_S1_ and to a lesser extent *E*_T1_. This results in larger *E*_ST_ for the **PTZ(SO_2_)** compounds **3** and **4**. The D‐A structure of **5** has served to greatly suppress *E*_S1_ making it the lowest in the series **1**–**5** while the *E*_T1_ is nearly identical to that of **2** which implies local excited state character to the triplet state.

### Photophysics

**Figure 10:**
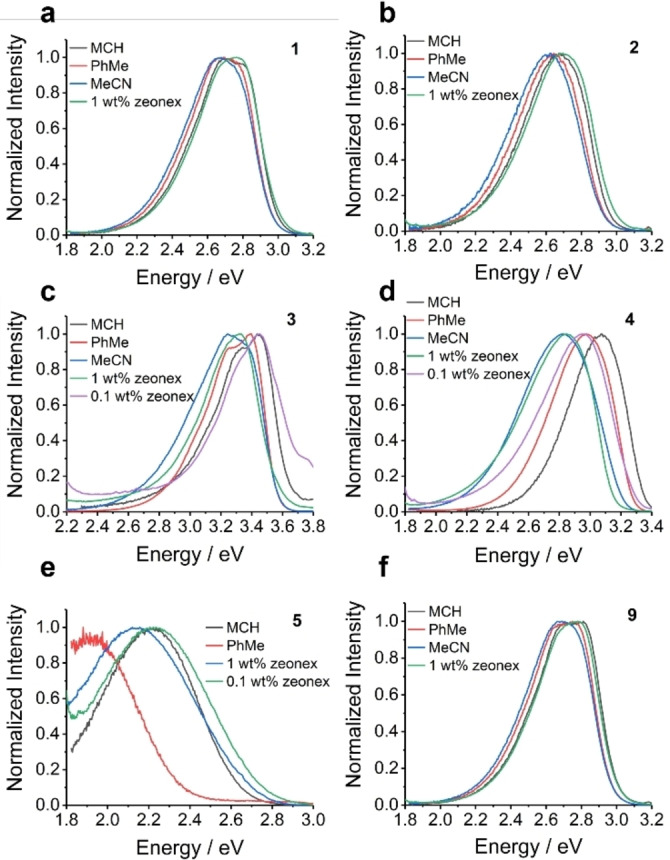
The steady‐state photoluminescence in doped zeonex films and solutions of 40 μM concentration. (a) and (b) are the two D‐D dimers **1** and **2** that show no solvatochromism. (c) shows an A‐A dimer **3** and shows no solvatochromism and (d) is the A‐A dimer **4** that has a minor bathochromic shift with increasing solvent polarity. (e) is the D‐A molecule **5** that has strong solvatochromism. (f) shows the emission for the D‐D compound **9**, that exhibits no solvatochromism.

The steady‐state photoluminescence of the compounds both in solution and film are shown in Figure 10. As expected, only compound **5** shows significant solvatochromism with increasing solvent polarity indicating a strongly ICT based emission because of its D‐A character. Compound **4** has a slight bathochromic shift indicative of some charge transfer character in the excited states. This will arise from transitions of the *n*‐electrons of the N atom towards the electron‐poor central biphenyl moiety which bears four strong inductively electron‐withdrawing sulfone groups. The other D‐D and A‐A dimer molecules exhibit no solvatochromism. The absorption and excitation spectra of the compounds as a function of solvent polarity are shown in Figures S7.1 and S7.2 respectively.

Comparing the emission spectra from solution to those of the doped zeonex films there is a notable contrast between the **PTZ** dimers and the **PTZ(SO_2_)** dimers. For **PTZ** dimers **1**, **2** and **9** the emission from the 1 wt.% zeonex films matches closely to the emission from the methylcyclohexane (MCH) solutions, whereas the same concentration films for **PTZ(SO_2_)** containing compounds **3**, **4** and **5** have an emission spectrum that is redshifted compared to the MCH solution. On reduction of the doping concentration to 0.1 wt.% for these compounds the emission then matches the MCH solution. Therefore, any matrix effects of the zeonex can be discounted as being responsible for the redshift and we conclude that this is instead due to intermolecular aggregation at higher concentrations. The UV‐Vis absorption spectra of the compounds as a function of doping concentration in zeonex films are shown in Figure S7.3. The photoluminescence spectra for these doped films and neat films are shown in Figure S7.4 The observation of π‐π stacking in the crystal packing of the **PTZ(SO_2_)**‐containing **3**–**5**, but not in **1**, **2** and **9**, adds weight to this conclusion.

As mentioned in the introduction, **PTZ** is known to induce RTP and the computational results indicated that compound **5** has a relatively narrow *E*_ST_ which may also facilitate TADF. We performed further experiments on the dimers to confirm whether these emission processes are observed.

**Figure 11:**
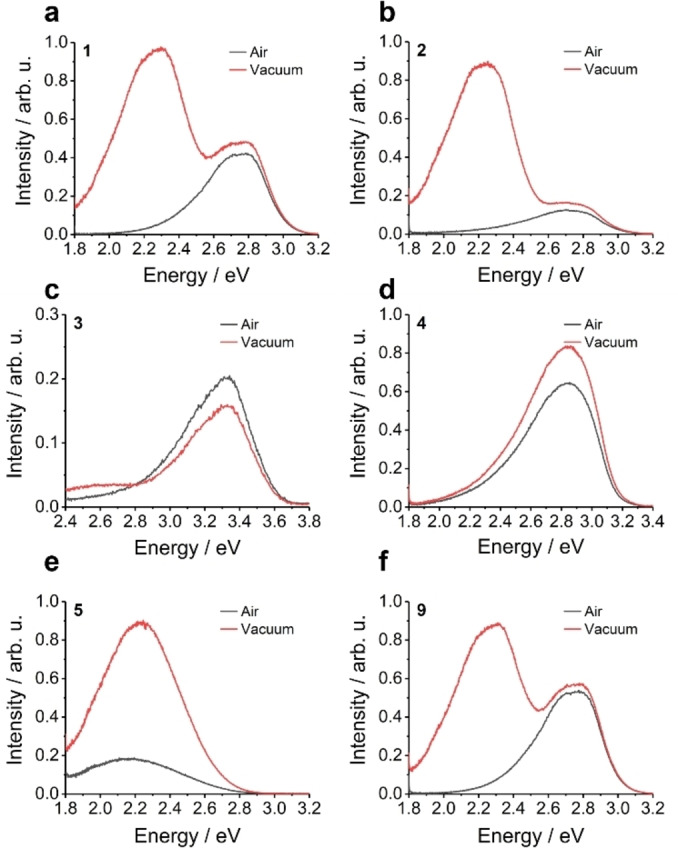
The air and vacuum steady‐state photoluminescence of the molecules in 1 wt.% zeonex films. All but **3** and **4** show significant contribution from an oxygen‐quenched species. Of these remaining molecules only **5** shows the oxygen dependent emission at the same energy of the fluorescence. (a‐b) Fluorescence and room temperature phosphorescence in air and in vacuum of molecules **1** and **2** (c‐d) Fluorescence of molecules **3** and **4** in air and in vacuum (e) Oxygen dependent emission and fluorescence of molecule **5** in air and in vacuum (f) the room temperature phosphorescence and fluorescence of molecule **9** in air and in vacuum.

Both TADF and RTP are triplet mediated and are therefore more efficient without the presence of oxygen, therefore the doped 1 wt.% zeonex films were measured both in air and under vacuum to observe the effect of oxygen on the emission processes (Figure 11). For the D‐D dimers **1**, **2** and **9** a low energy emission appeared with the removal of oxygen. This was attributed to an RTP process due to the significant redshift with respect to the fluorescence. The band profile of the two A‐A dimers **3** and **4** showed no significant changes when comparing the air and vacuum measurements rendering them both RTP and TADF inactive. Returning to the TDDFT analysis, the root sum squared spin orbit coupling matrix elements (SOCME, Table 4) between S_0_ and T_1_ indicate that there is a much larger SOC for the **PTZ** containing molecules compared to those featuring **PTZ(SO_2_)** heterocycles. We conclude that chemical oxidation reduces SOC which explains why RTP is observed for **1**, **2**, and **5**, but not for **3** and **4**.

The *E*_ST_ for all compounds (in this case defined as the gap between the prompt photoluminescence and phosphorescence onsets) are shown in Table 5 and Figure S7.14. **1**–**4** and **9** do not have an obvious ICT structure and their *E*_ST_ is also quite large therefore TADF is not observed. The behaviour of D‐A molecule **5** shows no major changes in the band position or width but does display a significant increase in intensity with the removal of oxygen, consistent with triplet mediated emission. Considering that there is only a limited shift in the spectrum with the removal of oxygen and that **5** does display ICT and a relatively narrow *E*_ST_ TADF seems the likely cause of this increase in intensity. However, if RTP and TADF are competing it is difficult to ascribe this emission to either mechanism using steady‐state measurements.

**Table 5:** Prompt (*E*_S_) and delayed emission (*E*_T_) onset energies and the estimated *E*_ST_.

	*E*_S_ [eV]^[a]^	*E*_T_ [eV]^[a]^	*E*_ST_ [eV]
**1**	2.98	2.60	0.38
**2**	2.88	2.60	0.28
**3**	3.07	2.82^[b]^	0.25
**4**	3.05	2.93^[b]^	0.12
**5**	2.71	2.58	0.13
**9**	2.97	2.58	0.39

[a] Data was obtained from measurements of 1 wt.% zeonex films except [b] taken from 0.1 wt.% zeonex films to minimise the influence of molecular aggregates in the film on the delayed emission.

Time‐resolved spectra were obtained to reveal the temporal resolution of the delayed emission and the energy of the phosphorescence of all the molecules. Time‐resolved spectra were measured at room temperature (RT) and 80 K (Figures S7.5–7.13). Molecules **3** and **4** exhibited no delayed emission and can be classified as TADF and RTP inactive. As expected from the steady‐state measurements under vacuum **PTZ** dimers **1**, **2** and **9** show RTP as seen in the energetics of the time resolved spectra (Figures S7.5, S7.6 and S7.13).

**Figure 12:**
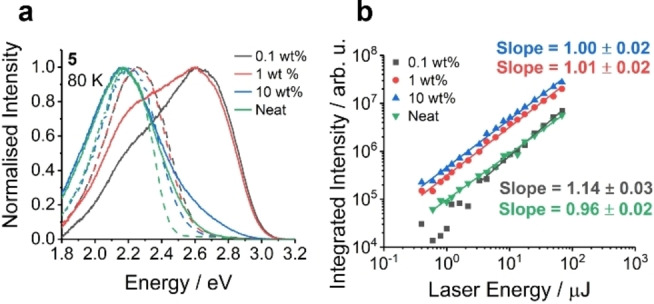
a) The prompt fluorescence and phosphorescence of compound **5** at 80 K in zeonex films of 0.1 wt.%, 1 wt.% and 10 wt.% and neat film. b) The laser fluence of these films measured at room temperature in the delayed emission region. The slopes of approximately 1 confirm a monomolecular process and thus the delayed fluorescence can be ascribed to TADF as opposed to a bimolecular process such as triplet‐triplet annihilation.

As mentioned above, D‐A molecule **5** is the most interesting, firstly due to its charge transfer behaviour and secondly due to its concentration dependent emission. Figure 12 shows the shift in the triplet and singlet state of molecule **5** as a function of concentration meaning it has a tuneable energy gap (the full time resolved spectra of the films are shown in Figures S7.9–7.12). The laser fluence of the various concentration films of **5** all show a linear relationship demonstrating the monomolecular nature of the delayed fluorescence and strongly implying that it is TADF. The change in the kinetics at RT and 80 K are shown in Figure S7.15 (as for all of the other compounds), which seems to indicate a shift to lower energy emission and shorter lifetimes. Comparing this temporal data with the shifts shown in Figure 9 it could be that at higher concentrations **5** forms aggregates and the emission starts to be dominated by this lower energy species.

In Figure 12 the dilute films at 80 K have a clear high energy onset (consistent with the steady state photoluminescence in Figure S7.4) compared to the 10 wt.% and neat film. This is more prominent at 80 K and thus suggests a dynamic process. It could be that at 80 K some relaxation mechanism is restricted or otherwise slowed down providing this higher energy emission, or that the formation of some aggregate species is also slowed down. Also, there is very limited shift in the energy of the phosphorescence as a function of concentration and the delayed fluorescence is either the same or very similar for each concentration. Therefore, it is suggested that any concentration dependence observed in the luminescence of these systems is probably related to the notorious conformation effects of **PTZ** systems[^2^, ^26^, ^27^, ^59^, ^60^, ^61^, ^62^] and arises from a combination of relaxation and aggregation effects which can be slowed down with decreasing temperature.

## Conclusion

The series of compounds **1**–**5** (and **9**) show a fascinating progression between delayed emission inactivity and activity through simple modifications to their chemical structures. In this series the A‐A compounds (**3** and **4**) have no delayed emission; it is only those compounds that feature donor molecules either in D‐D (**1**, **2** and **9**) or D‐A configuration (**5**) that show delayed emission. More interestingly, the D‐D compounds show only room temperature phosphorescence, whereas the D‐A compound that has charge transfer character displays much more complicated delayed emission phenomena. Compound **5**, especially at the higher concentrations, has a very small singlet‐triplet gap which makes it more difficult to clearly identify the phosphorescence cleanly. However, even at the 1 wt.% zeonex it still far outperforms all of the other compounds regarding its delayed contribution. This study adds new scope to the versatility of phenothiazine as a building block for luminescent materials.

## Experimental Section

Additional experimental details, supporting figures, packing diagrams and single‐crystal data, and NMR spectra are provided in the Supporting Information. Deposition Numbers 1888002 (for **1**), 1888003 (for **2**), 1888004 (for **3**), 1888022 (for **4**), 1888005 (for **5**) 1888006 (for **9**) contain the supplementary crystallographic data for this paper. These data are provided free of charge by the joint Cambridge Crystallographic Data Centre and Fachinformationszentrum Karlsruhe Access Structures service.

## Conflict of interest

There are no conflicts of interest.

1

## Supporting information

As a service to our authors and readers, this journal provides supporting information supplied by the authors. Such materials are peer reviewed and may be re‐organized for online delivery, but are not copy‐edited or typeset. Technical support issues arising from supporting information (other than missing files) should be addressed to the authors.

Supporting Information

## Data Availability

The data that support the findings of this study are available from the corresponding author upon reasonable request.
