# Supplementary material for: Oxidation State Tuning of Room Temperature Phosphorescence and Delayed Fluorescence in Phenothiazine and Phenothiazine‐5,5‐dioxide Dimers
Source: Chemistry. 2023 Apr 19;29(30):e202300428. doi: 10.1002/chem.202300428 (PMC10946842; doi:10.1002/chem.202300428)
Supplement: Supplementary file 1 — Supporting Information [file CHEM-29-0-s001.pdf]

Chemistry–A European Journal

Supporting Information

Oxidation State Tuning of Room Temperature Phosphorescence and Delayed Fluorescence in Phenothiazine and Phenothiazine-5,5-dioxide Dimers

Iain A. Wright,* Marc K. Etherington, Andrei S. Batsanov, Andrew P. Monkman,* and Martin R. Bryce*

Supporting information

Oxidation State Tuning of Room Temperature Phosphorescence and Delayed Fluorescence in Phenothiazine and Phenothiazine-5,5-dioxide Dimers

Iain A. Wright,*[a,b] Marc K. Etherington,[c,d] Andrei S. Batsanov,[a] Andrew P. Monkman*[a] and Martin R. Bryce*[a]

[a]Department of Chemistry, Durham University, South Road, Durham, DH1 3LE (UK)

[b]School of Chemistry, University of Edinburgh, David Brewster Road, Edinburgh, EH9 3FJ (UK)

[c]Department of Physics, Durham University, South Road, Durham, DH1 3LE (UK)

[d]Department of Mathematics, Physics and Electrical Engineering, Northumbria University, Ellison Place, Newcastle-upon-Tyne, NE1 8ST (UK)

Table of Contents

1. Experimental Section S2
2. Synthetic Methods S3
3. ^{1}H and ^{13}C NMR Spectra S12
4. X-ray Crystallography S26
5. Electrochemistry S34
6. Computational Results S35
7. Photophysics S43
8. References S51

1. Experimental Section

General Considerations

All commercially available chemicals were used without further purification. The catalyst [1,3-bis(2,6-diisopropylphenyl)imidazol-2-ylidene](3-chloropyridyl)palladium(II) dichloride (Pd-PEPPSI-*i*Pr) was synthesized according to literature protocols.[1] Reactions requiring an inert atmosphere were performed under a blanket of argon gas, which was dried over a phosphorus pentoxide column. Anhydrous solvents were dried through an HPLC column on an Innovative Technology Inc. solvent-purification system. Column chromatography was performed using 40–60 μm mesh silica gel. Analytical thin-layer chromatography (TLC) was performed on plates precoated with silica gel (Merck, silica gel 60F254) and visualized using UV light (254, 315, 365 nm).

NMR spectra were recorded on Bruker Avance 400 MHz and Varian Mercury 400 MHz spectrometers. Melting points were determined in open-ended capillaries using a Gallenkamp MPD-350 melting point apparatus at a ramping rate of 1 °C/min and are uncorrected. Atmospheric solids analysis probe (ASAP) mass spectra were recorded on a Waters Xevo QTOF spectrometer. Elemental analyses were obtained on an Exeter Analytical Inc. CE-440 elemental analyzer.

Cyclic voltammetry was recorded using a Princeton Applied Research VersaSTAT 3. A glassy carbon disk, Pt wire and Ag/Ag^+ ($AgNO_3$ in acetonitrile) were used as the working, counter, and reference electrodes respectively. Measurements were corrected to the ferrocene/ferrocenium redox couple. A 4:1 mixture of acetonitrile and methylene chloride was used as solvent with an analyte molarity of ca. 10^{-4} M in the presence of 10^{-1} M (n-Bu_4N)(PF_6) as a supporting electrolyte. Solutions were degassed with Ar and experiments run under a blanket of Ar.

UV−vis absorbance spectra were measured using a UV-3600 double beam spectrophotometer (Shimadzu). Photoluminescence (PL) spectra of solutions were recorded using a Fluorolog (Jobin-Yvon Horiba) system. Photoluminescence decays in film and solution were recorded using nanosecond gated luminescence and lifetime measurements (from 400 ps to 1 s) using the third harmonic of a high-energy pulsed Nd:YAG laser emitting at 355 nm (EKSPLA) as an excitation source. The emitted light was focused onto a spectrograph and detected with a sensitive gated iCCD camera (Stanford Computer Optics) with sub-nanosecond resolution. Time-resolved measurements were performed by exponentially increasing gate and integration times. Further details are available in reference 2.[2] Low temperature experiments were conducted using a liquid nitrogen cryostat VNF-100 (sample in flowing vapour, Janis Research) under nitrogen atmosphere, while measurements at room temperature were recorded under vacuum in the same cryostat. Thin films were deposited from toluene solutions through drop casting and dried under vacuum at room temperature.

2. Synthetic Methods

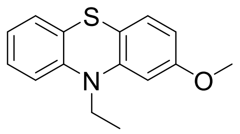

10-Ethyl-2-methoxy-10*H*-phenothiazine (7) Potassium *tert*-butoxide (0.67 g, 6 mmol) was added to a solution of 2-methoxy-10*H*-phenothiazine **6** (1.15 g, 5 mmol) in DMF (25 mL) and the reaction was left to stir for 1 h. Ethylbromide (1.65 g, 10 mmol) was then added and the reaction was heated to reflux overnight. Upon cooling the reaction was poured into water (50 mL) prior to addition of 2M HCl (aq.) (50 mL). The mixture was transferred to a separating funnel and extracted with EtOAc (3 × 30 mL). The combined organic extracts were washed with water (3 × 75 mL) and brine before drying over $MgSO_4$ and removal of the solvent under reduced pressure.

Purification was achieved by column chromatography on SiO_2 (eluting 10% EtOAc/hexane) to give **7** as an off-white solid (1.05 g, 82%). mp. 101–102 °C

^{1}H-NMR (400 MHz, acetone-*d*6): δ = 7.22 – 7.15 (m, 1H), 7.12 (dd, J = 7.6, 1.6 Hz, 1H), 7.01 (d, J = 8.3 Hz, 1H), 6.92 (t, J = 7.5 Hz, 1H), 6.57 (d, J = 2.5 Hz, 1H), 6.55 (dd, J = 8.3, 2.5 Hz, 1H), 3.98 (q, J = 6.9 Hz, 2H), 3.78 (s, 3H), 1.37 (t, J = 6.9 Hz, 3H)

^{13}C-NMR (101 MHz, acetone-*d*6): δ = 161.0, 147.4, 145.7, 128.2, 128.1, 127.8, 125.7, 123.2, 116.4, 115.7, 107.9, 103.7, 55.7, 42.3, 13.3

MS (ASAP): m/z = 258.1 $[M+H]^+$

HRMS (ASAP): m/z = calculated for $C_{15}H_{16}NOS$ $[M+H]^+$: 258.0953; found: 258.0951

Elemental analysis: Found C, 69.95; H, 5.87; N, 5.40 Calculated C, 70.01; H, 5.88; N, 5.44

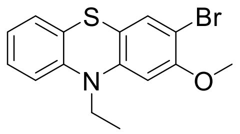

3-Bromo-10-ethyl-2-methoxy-10*H*-phenothiazine (8) A solution of **7** (1 g, 3.7 mmol) in a mixture of AcOH (10 mL) and $CHCl_3$ (10 mL) was cooled to 0 °C in the dark. Over a period of 1 h, NBS (0.66 g, 3.7 mmol) was added to the reaction mixture in three portions before the mixture was allowed to warm up to room temperature and left to stir for 3 h.

Purification was achieved by column chromatography on SiO_2 (eluting 0-20% EtOAc/hexane) to give **8** as a white solid (0.78 g, 63%). mp. 115–116 °C

^{1}H-NMR (400 MHz, acetone-*d*6): δ = 7.24 (s, 1H), 7.20 (ddd, J = 8.2, 7.3, 1.5 Hz, 1H), 7.14 (dd, J = 7.6, 1.5 Hz, 1H), 7.03 (dd, J = 8.3, 1.2 Hz, 1H), 6.95 (td, J = 7.5, 1.2 Hz, 1H), 6.75 (s, 1H), 4.04 (q, J = 7.0 Hz, 2H), 3.91 (s, 3H), 1.39 (t, J = 6.9 Hz, 3H)

^{13}C-NMR (101 MHz, acetone-*d*6): δ = 156.7, 147.1, 145.6, 130.9, 128.3, 128.0, 125.3, 123.6, 117.1, 116.7, 104.1, 102.1, 56.8, 42.5, 13.4

MS (ASAP): m/z = 336.0 $[M+H]^+$

HRMS (ASAP): m/z = calculated for $C_{15}H_{14}NOSBr$ $[M]^+$: 334.9979; found: 334.9980

Elemental analysis: Found C, 53.56; H, 4.19; N, 4.12 Calculated C, 53.58; H, 4.20; N, 4.17

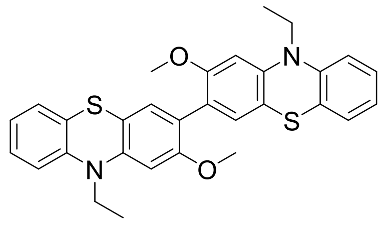

3,3'-Bi(10-ethyl-2-methoxy-10*H*-phenothiazine) (9) Isolated from the same reaction as **8** by slowly increasing the polarity of the column eluent to 40% EtOAc/hexane. The slowly moving brightly fluorescent band was collected in test tubes from which **9** crystallized spontaneously as pale yellow/green needles which were collected by filtration (70 mg, 7%). mp. 243–244 °C

NB. 5 drops of CS_2 had to be added to the NMR sample in order to obtain well resolved spectra

^{1}H-NMR (400 MHz, DMSO-*d*6): δ = 7.18 (ddd, J = 8.2, 7.3, 1.6 Hz, 2H), 7.09 (dd, J = 7.6, 1.5 Hz, 2H), 7.00 (dd, J = 8.2, 0.9 Hz, 2H), 6.92 (td, J = 7.5, 1.1 Hz, 2H), 6.78 (s, 2H), 6.63 (s, 2H), 4.00 (q, J = 6.9 Hz, 4H), 3.72 (s, 6H), 1.38 (t, J = 6.9 Hz, 6H)

^{13}C-NMR (101 MHz, DMSO-*d*6): δ = 156.6, 144.9, 144.2, 128.8, 127.1, 126.8, 124.0, 122.2, 120.5, 115.3, 112.9, 100.2, 55.6, 41.2, 12.9

MS (ASAP): m/z = 513.1 $[M+H]^+$

HRMS (ASAP): m/z = calculated for $C_{30}H_{29}N_2O_2S_2$ $[M+H]^+$: 513.1670; found: 513.1647

Elemental analysis: Found C, 70.20; H, 5.58; N, 5.35 Calculated C, 70.28; H, 5.51; N, 5.46

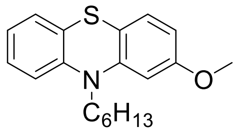

10-Hexyl-2-methoxy-10*H*-phenothiazine (11) Under an atmosphere of nitrogen, KOH (1.71 g, 30 mmol) was added to a solution of 2-methoxy-10*H*-phenothiazine **6** (2.29 g, 10 mmol), 1-bromohexane (2.48 g, 2.11 mL, 15 mmol) and a few crystals of KI in 40 mL dimethyl sulfoxide (DMSO) and the reaction was stirred at room temperature for 5 h. Water (200 mL) was added and the crude product was extracted into Et_2O (3 × 75 mL). The combined organic extracts were washed with NH_4Cl (satd.) (100 mL) and water (100 mL) before being dried over $MgSO_4$. The solvent was then removed under reduced pressure.

Purification was achieved by column chromatography on SiO_2 (eluting 0-5% EtOAc/hexane basified with 1% NEt_3) to yield **11** as a very pale yellow oil (3.07 g, 98%).

^{1}H-NMR (400 MHz, acetone-*d*6): δ = 7.18 (ddd, *J* = 8.3, 7.3, 1.6 Hz, 1H), 7.13 (dd, *J* = 7.6, 1.5 Hz, 1H), 7.02 (d, *J* = 8.3 Hz, 1H) overlapping with 7.02 (d, *J* = 8.3 Hz, 1H), 6.92 (td, *J* = 7.5, 1.2 Hz, 1H), 6.59 (d, *J* = 2.4 Hz, 1H), 6.55 (dd, *J* = 8.3, 2.5 Hz, 1H), 3.94 (t, *J* = 7.0 Hz, 2H), 3.78 (s, 3H), 1.89 – 1.70 (m, 2H), 1.46 (pentet, *J* = 7.1 Hz, 2H), 1.35 – 1.23 (m, 4H), 0.85 (t, 7.2 Hz, 3H)

^{13}C-NMR (101 MHz, acetone-*d*6): δ = 161.0, 147.8, 146.1, 128.3, 128.1, 127.9, 126.3, 123.2, 116.8, 116.3, 108.1, 104.0, 55.7, 47.8, 32.2, 27.6, 27.1, 23.3, 14.2

MS (ASAP): m/z = 314.1 $[M+H]^+$

HRMS (ASAP): m/z = calculated for $C_{19}H_{24}NOS$ $[M+H]^+$: 313.1500; found: 314.1579

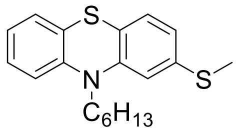

10-Hexyl-2-(methylsulfanyl)-10*H*-phenothiazine (12) Under an atmosphere of nitrogen, KOH (3.42 g, 60 mmol) was added to a solution of 2-(methylsulfanyl)-10*H*-phenothiazine **10** (4.91 g, 20 mmol), 1-bromohexane (4.96 g, 4.22 mL, 30 mmol) and a few crystals of KI in 80 mL dimethyl sulfoxide (DMSO) and the reaction was stirred at room temperature for 5 h. Water (400 mL) was added and the crude product was extracted into Et_2O (3 × 150 mL). The combined organic extracts were washed with NH_4Cl (satd.) (200 mL) and water (200 mL) before being dried over $MgSO_4$. The solvent was then removed under reduced pressure.

Purification was achieved by column chromatography on SiO_2 (eluting 0-5% EtOAc/hexane basified with 1% NEt_3) to yield **12** as a yellow oil (6.47 g, 98%).

^{1}H-NMR (400 MHz, acetone-*d*6): δ = 7.19 (ddd, *J* = 8.2, 7.3, 1.6 Hz, 1H), 7.13 (dd, *J* = 7.6, 1.5 Hz, 1H), 7.06 (d, *J* = 8.0 Hz, 1H), 7.03 (dd, *J* = 8.3, 1.2 Hz, 1H), 6.94 (td, *J* = 7.5, 1.2 Hz, 1H), 6.89 (d, *J* = 1.9 Hz, 1H), 6.86 (dd, *J* = 8.0, 1.9 Hz, 1H), 3.96 (t, *J* = 7.0 Hz, 2H), 2.49 (s, 3H), 1.86 – 1.70 (m, 2H), 1.46 (pentet, *J* = 7.1 Hz, 2H), 1.34 – 1.26 (m, 4H), 0.85 (t, *J* = 7.2 Hz, 3H)

^{13}C-NMR (101 MHz, acetone-*d*6): δ = 146.0, 145.1, 138.1, 127.4, 127.2, 127.1, 124.8, 122.5, 121.4, 120.4, 116.0, 114.0, 46.9, 31.3, 26.6, 26.2, 22.4, 15.0, 13.3

MS (ASAP): m/z = 330.1 $[M+H]^+$

HRMS (ASAP): m/z = calculated for $C_{19}H_{24}NS_2$ $[M+H]^+$: 330.1350; found: 330.1350

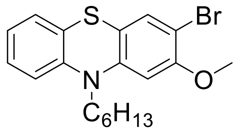

3-Bromo-10-hexyl-2-methoxy-10*H*-phenothiazine (13) In the dark, NBS (1.65 g, 9.23 mmol) was added in 3 portions over 45 minutes to a solution of **11** (2.90 g, 9.23 mmol), in THF (200 mL) at 0 °C. The reaction was stirred at this temperature for 1 h then allowed to warm to room temperature overnight. Water (200 mL) was added and the crude product was extracted into Et_2O (3 × 75 mL). The combined organic extracts were dried over $MgSO_4$ and the solvent was removed under reduced pressure.

Purification was achieved by column chromatography on SiO_2 (0-10% EtOAc/hexane basified with 1% NEt_3) to yield **13** as a pale yellow oil (3.60 g, 99%).

^{1}H-NMR (400 MHz, acetone-*d*6): δ = 7.25 (s, 1H), 7.20 (ddd, J = 8.2, 7.3, 1.6 Hz, 1H), 7.14 (dd, J = 7.6, 1.5 Hz, 1H), 7.04 (dd, J = 8.3, 1.2 Hz, 1H), 6.95 (td, J = 7.5, 1.2 Hz, 1H), 6.76 (s, 1H), 3.99 (t, J = 6.9 Hz, 2H), 3.90 (s, 3H), 1.84 – 1.75 (m, 2H), 1.51 – 1.39 (m, 2H), 1.34 – 1.24 (m, 4H), 0.84 (t, J = 7.1, 3H)

^{13}C-NMR (101 MHz, acetone-*d*6): δ = 156.7, 147.5, 146.0, 130.9, 128.3, 128.1, 125.8, 123.6, 117.7, 117.0, 104.1, 102.4, 56.8, 48.0, 32.1, 27.6, 27.1, 23.3, 14.2

MS (ASAP): m/z = 392.1 [M+H]

HRMS (ASAP): m/z = calculated for $C_{19}H_{23}NOSBr$ $[M+H]^+$: 392.0684; found: 392.0672

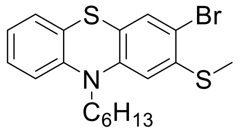

3-Bromo-10-hexyl-2-(methylsulfanyl)-10*H*-phenothiazine (14) In the dark, NBS (3.49 g, 19.60 mmol) was added in 3 portions over 45 minutes to a solution of **12** (6.47 g, 19.60 mmol), in THF (300 mL) at 0 °C. The reaction was stirred at this temperature for 1 h then allowed to warm to room temperature overnight. Water (200 mL) was added and the crude product was extracted into Et_2O (3 × 75 mL). The combined organic extracts were dried over $MgSO_4$ and the solvent was removed under reduced pressure.

Purification was achieved by column chromatography on SiO_2 (0-10% EtOAc/hexane basified with 1% NEt_3) to yield **14** as an orange oil which solidified very slowly upon standing (7.34 g, 92%). mp. 55–57 °C

^{1}H-NMR (400 MHz, acetone-*d*6): δ = 7.28 (s, 1H), 7.21 (ddd, *J* = 8.2, 7.3, 1.6 Hz, 1H), 7.14 (dd, *J* = 7.7, 1.5 Hz, 1H), 7.05 (dd, *J* = 8.2, 1.2 Hz, 1H), 6.96 (td, *J* = 7.5, 1.2 Hz, 1H), 6.82 (s, 1H), 4.00 (t, *J* = 7.0 Hz, 2H), 2.54 (s, 3H), 1.80 (dt, *J* = 14.7, 7.5 Hz, 2H), 1.52 – 1.39 (m, 2H), 1.34 – 1.26 (m, 4H), 0.85 (t, *J* = 7.1 Hz, 3H)

^{13}C-NMR (101 MHz, acetone-*d*6): δ = 146.5, 145.8, 139.6, 130.7, 128.5, 128.1, 125.1, 123.7, 123.4, 117.1, 114.0, 113.9, 48.0, 32.2, 27.5, 27.1, 23.3, 15.8, 14.2

MS (ASAP): m/z = 408.0 $[M+H]^+$

HRMS (ASAP): m/z = calculated for $C_{19}H_{21}NS_2Br$ $[M–H]^+$: 406.0299; found: 406.0295

Elemental analysis: Found C, 55.55; H, 5.37; N, 3.52 Calculated C, 55.88; H, 5.43; N, 3.43

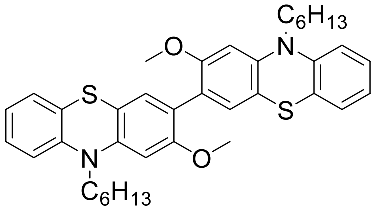

3,3'-Bi(10-hexyl-2-methoxy-10*H*-phenothiazine) (1) Under an atmosphere of nitrogen **13** (2.45 g, 6.24 mmol) and Pd-PEPPSI-*i*Pr (5 mol%, 211 mg, 0.31 mmol) were dissolved in dry toluene (80 mL) and degassed with stirring for 10 minutes. To this stirred mixture at room temperature was added a solution of *tert*-butyllithium (1.7 M in pentane, 2.94 mL, 4.99 mmol) over 1 h. The reaction was left to stir overnight then quenched with MeOH before the solvent was removed under reduced pressure. The residue was redissolved in CH_2Cl_2 (50 mL) and transferred to a separating funnel before being washed with water (100 mL). The aqueous phase was further extracted with CH_2Cl_2 (2 × 50 mL) and the combined organic phases were dried over $MgSO_4$ which was removed by filtration prior to evaporation of the solvent under reduced pressure.

Purification was achieved by column chromatography on SiO_2 (1:1 CH_2Cl_2/hexane basified with 1% NEt_3) followed by recrystallisation from EtOAc to yield **1** as colourless crystals (1.63 g, 84%). mp. 180–181 °C

^{1}H-NMR (400 MHz, acetone-*d*6): δ = 7.19 (ddd, J = 8.2, 7.4, 1.5 Hz, 2H), 7.14 (dd, J = 7.6, 1.5 Hz, 2H), 7.04 (dd, J = 8.2, 0.9 Hz, 2H), 6.93 (td, J = 7.5, 1.1 Hz, 2H), 6.90 (s, 2H), 6.73

(s, 2H), 4.01 (t, J = 7.0 Hz, 5H), 3.75 (s, 6H), 1.85 (dt, J = 14.7, 7.5 Hz, 4H), 1.55 – 1.44 (m, 4H), 1.40 – 1.27 (m, 8H), 0.86 (t, J = 7.1 Hz, 6H)

^{13}C-NMR (101 MHz, acetone-*d*6): δ = 158.2, 146.9, 146.3, 130.2, 128.04, 127.98 126.4, 123.2, 122.3, 116.7, 115.2, 101.5, 56.2, 47.9, 32.2, 27.8, 27.3, 23.3, 14.3

MS (ASAP): m/z = 625.3 $[M+H]^+$

HRMS (ASAP): m/z = calculated for $C_{38}H_{45}N_2S_2O_2$ $[M+H]^+$: 625.2922; found: 625.2932

Elemental analysis: Found C, 72.81; H, 7.13; N, 4.34 Calculated C, 73.04; H, 7.10; N, 4.48

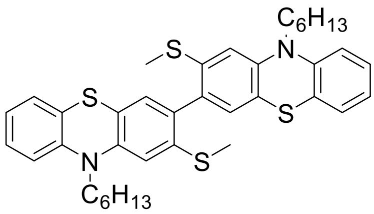

3,3'-Bi(10-hexyl-2-(methylsulfanyl)-10*H*-phenothiazine) (2) Under an atmosphere of nitrogen **14** (1.19 g, 2.92 mmol) and Pd-PEPPSI-*i*Pr (5 mol%, 102 mg, 0.15 mmol) were dissolved in dry toluene (50 mL) and degassed with stirring for 10 minutes. To this stirred mixture at room temperature was added a solution of *tert*-butyllithium (1.7 M in pentane, 1.38 mL, 2.34 mmol) over 1 h. The reaction was left to stir overnight then quenched with MeOH before the solvent was removed under reduced pressure. The residue was redissolved in CH_2Cl_2 (50 mL) and transferred to a separating funnel before being washed with water (100 mL). The aqueous phase was extracted with a further portion of CH_2Cl_2 (2 × 50 mL) and the combined organic phases were dried over $MgSO_4$ which was removed by filtration prior to evaporation of the solvent under reduced pressure.

Purification was achieved by column chromatography on SiO_2 (1:1 CH_2Cl_2/hexane basified with 1% NEt_3) to give **2** as an orange analytically pure oil which was dried under vacuum (910 mg, 95%). This material is very soluble in most organic solvents. Recrystallisation from Et_2O/pentane yielded a solid sample of the product as orange crystals (462 mg, 48%). mp. 148–149 °C

^{1}H-NMR (400 MHz, acetone-*d*6): δ = 7.21 (ddd, J = 8.2, 7.4, 1.5 Hz, 2H), 7.15 (dd, J = 7.6, 1.5 Hz, 2H), 7.06 (dd, J = 8.2, 0.8 Hz, 2H), 6.95 (td, J = 7.5, 1.1 Hz, 2H), 6.92 (s, 2H), 6.85 (s, 2H), 4.03 (t, J = 7.0 Hz, 4H), 2.37 (s, J = 2.8 Hz, 6H), 1.89 – 1.80 (m, 4H), 1.58 – 1.44 (m, 4H), 1.40 – 1.27 (m, 8H), 0.87 (t, J = 7.1 Hz, 6H)

^{13}C-NMR (101 MHz, acetone-*d*6): δ = 146.5, 145.9, 138.9, 133.3, 129.5, 128.3, 128.1, 125.6, 123.4, 121.3, 116.9, 113.9, 48.0, 32.2, 27.7, 27.2, 23.3, 16.0, 14.3

MS (ASAP): m/z = 657.2 $[M+H]^+$

HRMS (ASAP): m/z = calculated for $C_{38}H_{44}N_2S_4$ $[M]^+$: 656.2387; found: 656.2409

Elemental analysis: Found C, 69.65; H, 6.86; N, 4.09 Calculated C, 69.47; H, 6.75; N, 4.26

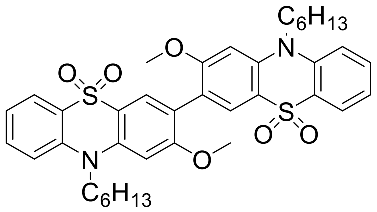

3,3'-Bi(10-hexyl-2-methoxy-10*H*-phenothiazine-*S*,*S*-dioxide) (3) *m*-CPBA (250 mg, 1.44 mmol) was added to a stirred solution of **1** (150 mg, 0.23 mmol) in CH_2Cl_2 (20 mL) held at 0 °C in an ice bath. After 15 minutes the cooling bath was removed and the reaction was allowed to warm to room temperature over 2 h. The reaction was quenched with 6M NaOH (10 mL) and stirred for a further 5 minutes. Water (30 mL) was added and the mixture transferred to a separating funnel and the product extracted with CH_2Cl_2 (3 × 30 mL). The combined organic extracts were dried over $MgSO_4$ which was removed by filtration prior to removal of solvent under reduced pressure.

Purification was achieved by recrystallisation from EtOAc yielding **3** as white crystals (103 mg, 62%). mp. 270–271 °C.

Single crystals suitable for X-ray analysis were obtained by liquid diffusion of hexane into the CDCl3 solution of **3** used to obtain the NMR spectra.

^{1}H-NMR (400 MHz, $CDCl_3$): δ = 8.12 (dd, J = 7.9, 1.6 Hz, 2H), 7.98 (s, 2H), 7.61 (ddd, J = 8.8, 7.3, 1.6 Hz, 2H), 7.34 (d, J = 8.5 Hz, 2H), 7.29 – 7.24 (m, 2H), 6.78 (s, 2H), 4.21 – 4.14 (m, 4H), 3.88 (s, 6H), 2.00 (dt, J = 15.3, 7.8 Hz, 4H), 1.58 – 1.48 (m, 4H), 1.46 – 1.33 (m, 8H), 0.93 (t, J = 7.1 Hz, 6H)

^{13}C-NMR (101 MHz, $CDCl_3$): δ = 161.0, 142.4, 141.0, 132.9, 126.9, 125.2, 123.6, 122.0, 120.6, 116.7, 116.0, 98.1, 56.1, 48.9, 31.6, 27.0, 26.7, 22.7, 14.1

MS (ASAP): m/z = 689.2 $[M+H]^+$

HRMS (ASAP): m/z = calculated for $C_{38}H_{44}N_2S_2O_6$ $[M]^+$: 688.2641; found: 688.2666

Elemental analysis: Found C, 66.22; H, 6.36; N, 4.04 Calculated C, 66.25; H, 6.44; N, 4.07

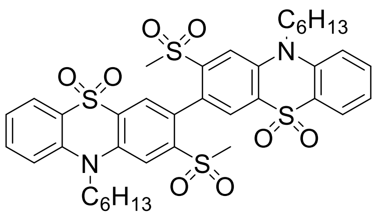

3,3'-Bi(10-hexyl-2-(methylsulfonyl)-10*H*-phenothiazine-*S*,*S*-dioxide) (4) *m*-CPBA (3.58 g, 20.78 mmol) was added to a stirred solution of **2** (0.91 g, 1.38 mmol) in CH_2Cl_2 (100 mL) held at 0 °C in an ice bath. After 15 minutes the cooling bath was removed and the reaction was allowed to warm to room temperature over 2 h. The reaction was quenched with 6M NaOH (50 mL) and stirred for a further 5 minutes. Water (100 mL) was added and the mixture transferred to a separating funnel and the product extracted with CH_2Cl_2 (3 × 75 mL). The combined organic extracts were dried over $MgSO_4$ which was removed by filtration prior to removal of solvent under reduced pressure.

The crude product was dissolved in CH_2Cl_2 and passed through a short plug of SiO_2 (eluting CH_2Cl_2 with 1% MeOH) and the solvent was removed to yield essentially pure **4** as a white powder (0.97 g, 90%). If required, further purification may be achieved by column

chromatography on SiO2 (eluting 50% EtOAc in hexane) followed by recrystallisation from EtOAc with a small amount of CH_2Cl_2 to give **4** as small white needles (214 mg, 20%). The mother liquor may be repeatedly concentrated to yield further crops of the product in order to minimize losses. mp. 296–297 °C

^{1}H-NMR (400 MHz, $CDCl_3$): δ = 8.20 (s, 2H), 8.16 – 8.11 (m, 4H), 7.73 (ddd, J = 8.8, 7.3, 1.6 Hz, 2H), 7.45 (d, J = 8.6 Hz, 2H), 7.39 (t, J = 7.6 Hz, 2H), 4.35 – 4.23 (m, 4H), 3.03 (s, 6H), 2.04 – 1.88 (m, 4H), 1.59 – 1.48 (m, 4H), 1.45 – 1.34 (m, 8H), 0.92 (t, J = 7.0 Hz, 6H)

^{13}C-NMR (101 MHz, $CDCl_3$): δ = 143.7, 141.3, 140.5, 134.0, 128.6, 128.1, 126.7, 124.7, 124.2, 123.3, 118.2, 116.7, 49.1, 45.8, 31.4, 27.0, 26.5, 22.7, 14.1

MS (ASAP): m/z = 785.2 $[M+H]^+$

HRMS (ASAP): m/z = calculated for $C_{38}H_{45}N_2S_4O_8$ $[M+H]^+$: 785.2059; found: 785.2073

Elemental analysis: Found C, 57.93; H, 5.59; N 3.49, Calculated C, 58.14; H, 5.65; N, 3.57

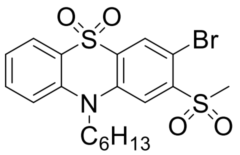

3-Bromo-10-hexyl-2-(methanesulfonyl)-10*H*-phenothiazine-*S*,*S*-dioxide (15) Hydrogen peroxide (30% w/w, 3.5 ml) was added to a solution of **14** (650 mg, 1.59 mmol) in AcOH whereupon the solution turned from colourless to blue. The reaction mixture was heated to 95 °C for 3 h during which time the reaction colour changed from blue to colourless to orange. The hot reaction mixture was poured over ice. Once the ice had completely melted the resulting emulsion was extracted with CH_2Cl_2 (3 × 50 mL). The combined organic phases were washed with water (3 × 100 mL) the dried over $MgSO_4$ and the solvent removed under reduced pressure.

Purification was achieved by recrystallisation from EtOH to give **15** as colourless crystals (518 mg, 69%). mp.151–152 °C

^{1}H-NMR (400 MHz, $CDCl_3$): δ = 8.42 (s, 1H), 8.23 (s, 1H), 8.12 (d, J = 7.9 Hz, 1H), 7.73 – 7.67 (m, 1H), 7.41 (d, J = 8.6 Hz, 1H), 7.37 (t, J = 7.6 Hz, 1H), 4.27 – 4.17 (m, 2H), 3.36 (s, 3H), 1.92 (dt, J = 15.4, 7.7 Hz, 2H), 1.49 (dt, J = 14.8, 7.4 Hz, 2H), 1.42 – 1.28 (m, 4H), 0.90 (t, J = 7.0 Hz, 3H)

^{13}C-NMR (101 MHz, $CDCl_3$): δ = 143.3, 140.5, 140.3, 134.1, 130.6, 128.5, 124.5, 124.2, 123.3, 119.7, 116.7, 110.7, 49.1, 42.5, 31.4, 27.0, 26.4, 22.7, 14.1

MS (ASAP): m/z = 472.0 $[M+H]^+$

HRMS (ASAP): m/z = calculated for $C_{19}H_{23}BrNO_4S_2$ $[M+H]^+$: 472.0252; found: 472.0234

Elemental analysis: Found C, 48.48; H, 4.71; N, 3.04 Calculated C, 48.31; H, 4.69; N, 2.96

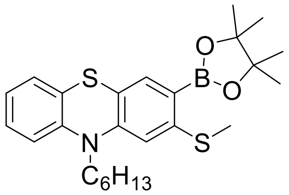

10-Hexyl-2-(methylsulfanyl)-3-(4,4,5,5-tetramethyl-1,3,2-dioxaborolan-2-yl)-10*H*-phenothiazine (16) Phenothiazine **14** was dissolved in dry THF (20 mL) and cooled to -78 °C. To the stirred solution was added *sec*-butyllithium (1.4 M in cyclohexane, 1.85 mL, 2.59 mmol) and the reaction left to stir for 1 h. Subsequently, 2-isopropoxy-4,4,5,5-tetramethyl-1,3,2-borolane (0.66 mL, 3.23 mmol) was added. After 15 min the cooling bath was removed and the reaction was allowed to warm to room temperature overnight. The reaction was quenched with H_2O (20 mL) and the crude product extracted with EtOAc (3 × 25 mL) and dried over $MgSO_4$ before the solvent was removed under reduced pressure.

After column chromatography on SiO_2 (eluting 10% EtOAc in hexane) an oil was obtained (512 mg) which comprised a mixture of 66% the desired product **16** (337 mg, 35% yield) and 34% dehalogenated precursor **12** as calculated using the –SCH_3 resonance in the ^{1}H-NMR spectrum occurring at 2.49 and 2.46 ppm for **12** and **16** respectively. The mixture thus obtained was used without further separation. Stacked NMR spectra of **16** and **12** with the resonances due to **12** clearly illustrated are shown below in Section 3 of this Supplementary Information (Section 3 NMR Spectra).

^{1}H-NMR (400 MHz, acetone-*d*6): δ = 7.36 (s, 1H), 7.21 – 7.16 (m, 1H), 7.12 (dd, J = 7.6, 1.5 Hz, 1H), 7.05 – 7.02 (m, 1H), 6.97 – 6.92 (m, 1H), 6.80 (s, 1H), 4.01 (t, J = 7.0 Hz, 2H), 2.46 (s, 3H), 1.86 – 1.76 (m, 2H), 1.52 – 1.43 (m, 2H), 1.32 (s, J = 4.3 Hz, 12H), 1.31 – 1.26 (m, 4H), 0.88 – 0.83 (m, 3H)

^{11}B NMR (128 MHz, acetone-*d*6): δ = 31.06.

MS (ASAP): m/z = 455.2 $[M]^+$

HRMS (ASAP): m/z = calculated for $C_{25}H_{34}BNO_2S_2$ $[M]^+$: 454.2160; found: 454.2169

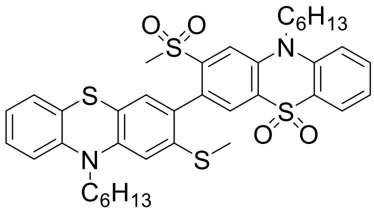

10,10’-Dihexyl-2-methanesulfonyl-2’-(methylsulfanyl)-10*H*,10’*H*-5λ^6-(3,3’biphenothiazine)-5,5-dione (5) Compound **15** (269 mg, 0.57 mmol), boronic ester **16** (247 mg, 0.54 mmol) and $Pd(PPh_3)_4$ (5 mol%, 31 mg, 0.03 mmol) were dissolved in a thoroughly degassed mixture of dimethoxyethane (10 mL) and H_2O (2 mL). K_2CO_3 (224 mg, 1.62 mmol) was then added and the reaction was heated to 90 °C for 14 h. Upon cooling H_2O (50 mL) was added and the reaction mixture was extracted into CH_2Cl_2 (3 × 25 mL) and dried over $MgSO_4$ before the solvent was removed under reduced pressure.

The crude product was purified by column chromatography on SiO_2 (eluting 20% EtOAc in hexane) followed by recrystallisation from EtOH to give pure **5** as yellow crystals (361 mg, 93%). mp. 186–187 °C

^{1}H-NMR (400 MHz, acetone-*d*6): δ = 8.32 (s, 1H), 8.07 (dd, J = 7.8, 1.4 Hz, 1H), 7.92 (s, 1H), 7.86 – 7.77 (m, 2H), 7.45 (ddd, J = 8.0, 6.7, 1.4 Hz, 1H), 7.26 – 7.20 (m, 1H), 7.15 (dd, J = 7.6, 1.5 Hz, 1H), 7.13 (s, 1H), 7.09 (d, J = 7.4 Hz, 1H), 7.00 – 6.95 (m, 2H), 4.54 – 4.45 (m, 2H), 4.07 (t, J = 7.1 Hz, 2H), 2.93 (s, 3H), 2.48 (s, 3H), 1.99 (m, 2H), 1.92 – 1.82 (m, 2H), 1.62 – 1.47 (m, 4H), 1.45 – 1.28 (m, 8H), 0.90 (t, *J* = 7.2 Hz, 3H) overlapping with 0.88 (t, *J* = 7.3 Hz, 3H)

^{13}C-NMR (101 MHz, acetone-*d*6): δ = 147.4, 145.6, 145.0, 141.6, 141.3, 139.1, 134.7, 131.8, 130.7, 129.6, 129.1, 128.4, 128.1, 128.0, 125.7, 125.4, 124.0, 123.67, 123.66, 121.1, 118.6, 118.3, 117.0, 113.3, 49.0, 48.1, 43.6, 32.2, 32.1, 27.6, 27.5, 27.2, 26.8, 23.3, 23.2, 15.9, 14.3, 14.2

MS (ASAP): m/z = 721.2 $[M+H]^+$

HRMS (ASAP): m/z = calculated for $C_{38}H_{45}N_2O_4S_4$ $[M+H]^+$: 721.2262; found: 721.2232

Elemental analysis: Found C, 63.34; H, 6.13; N, 3.83 Calculated C, 63.30; H, 6.15; N, 3.89

3. ^{1}H and ^{13}C NMR Spectra

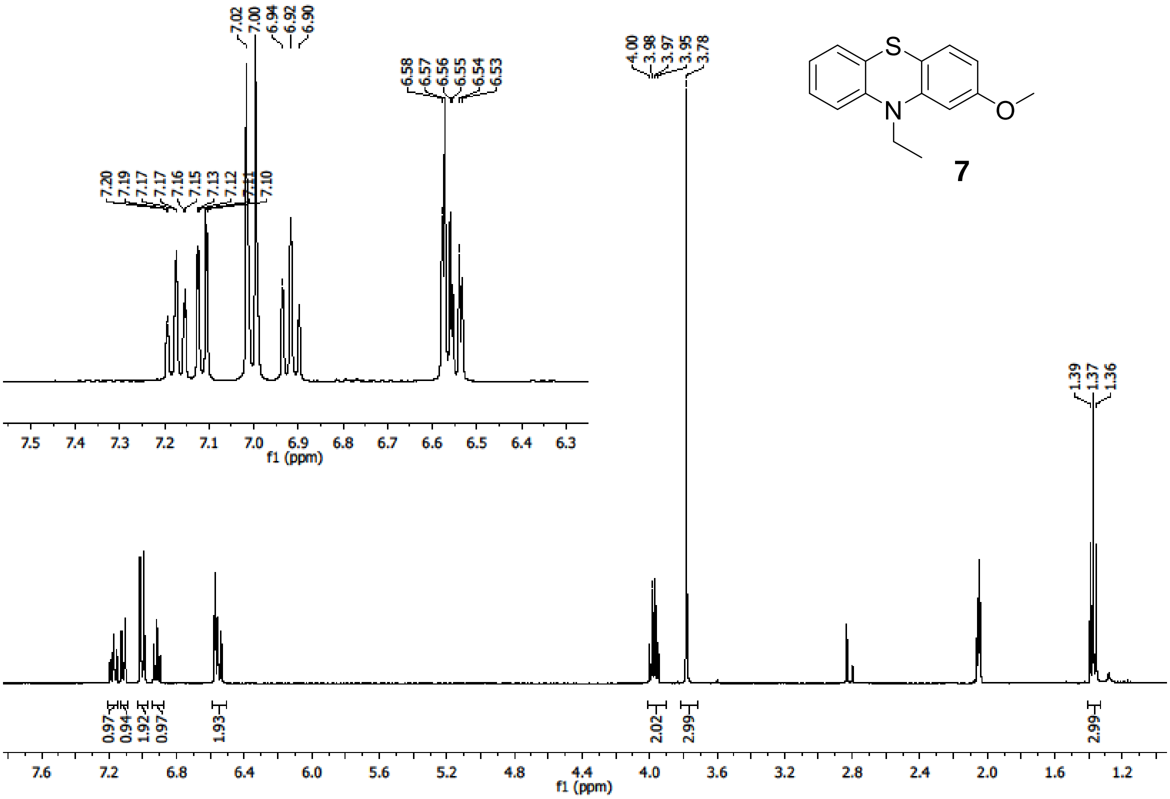

7

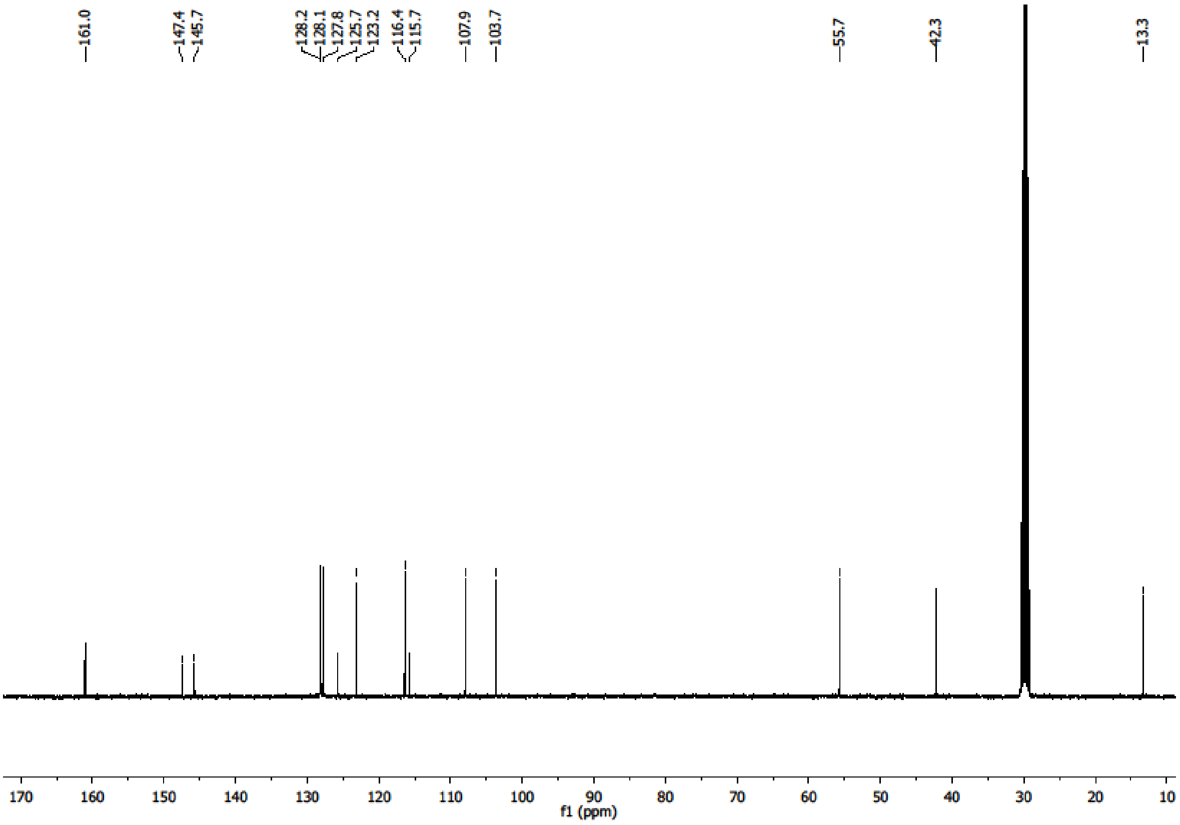

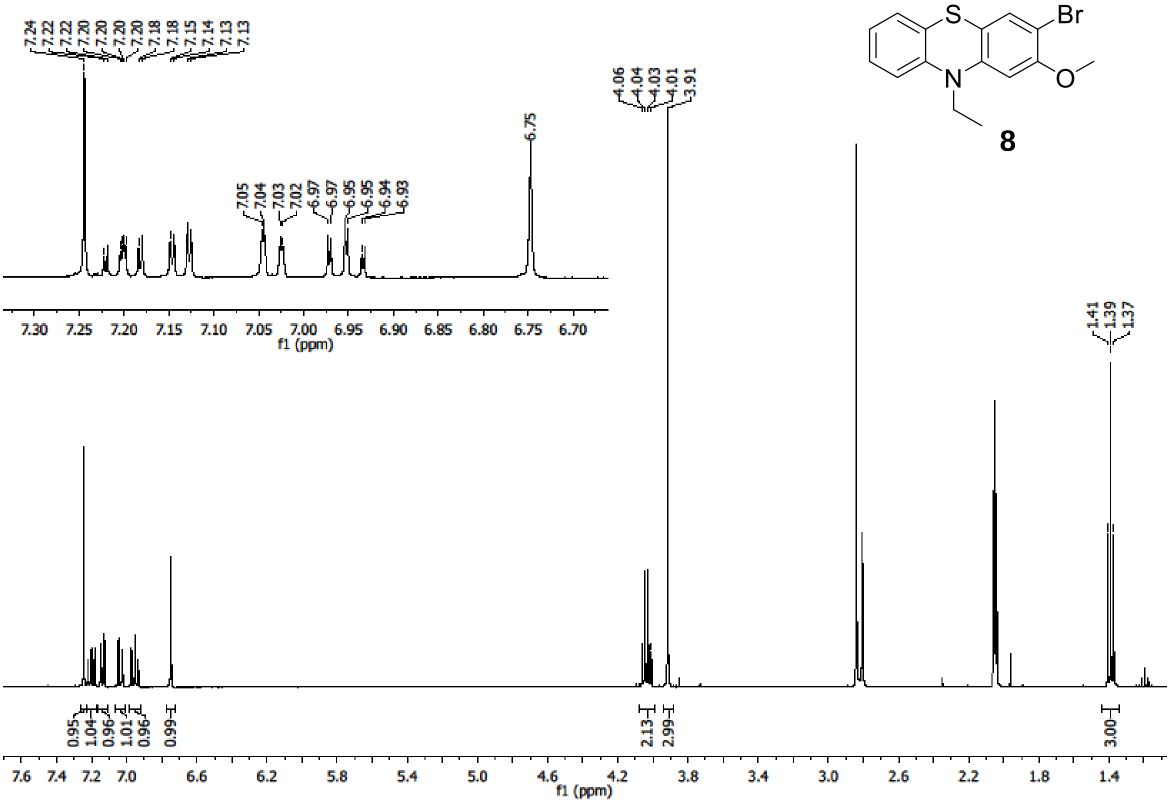
S
Br
N
O
7.24 7.22 7.22 7.20 7.20 7.20 7.20 7.18 7.18 7.15 7.14 7.13 7.13
7.05 7.04 7.03 7.02 6.97 6.97 6.95 6.95 6.94 6.93
6.75
4.06 4.04 4.03 4.01 3.91
1.41 1.39 1.37
0.95 1.04 0.96 1.01 0.96 0.99
2.13 2.99
3.00
f1 (ppm)
f1 (ppm)

8

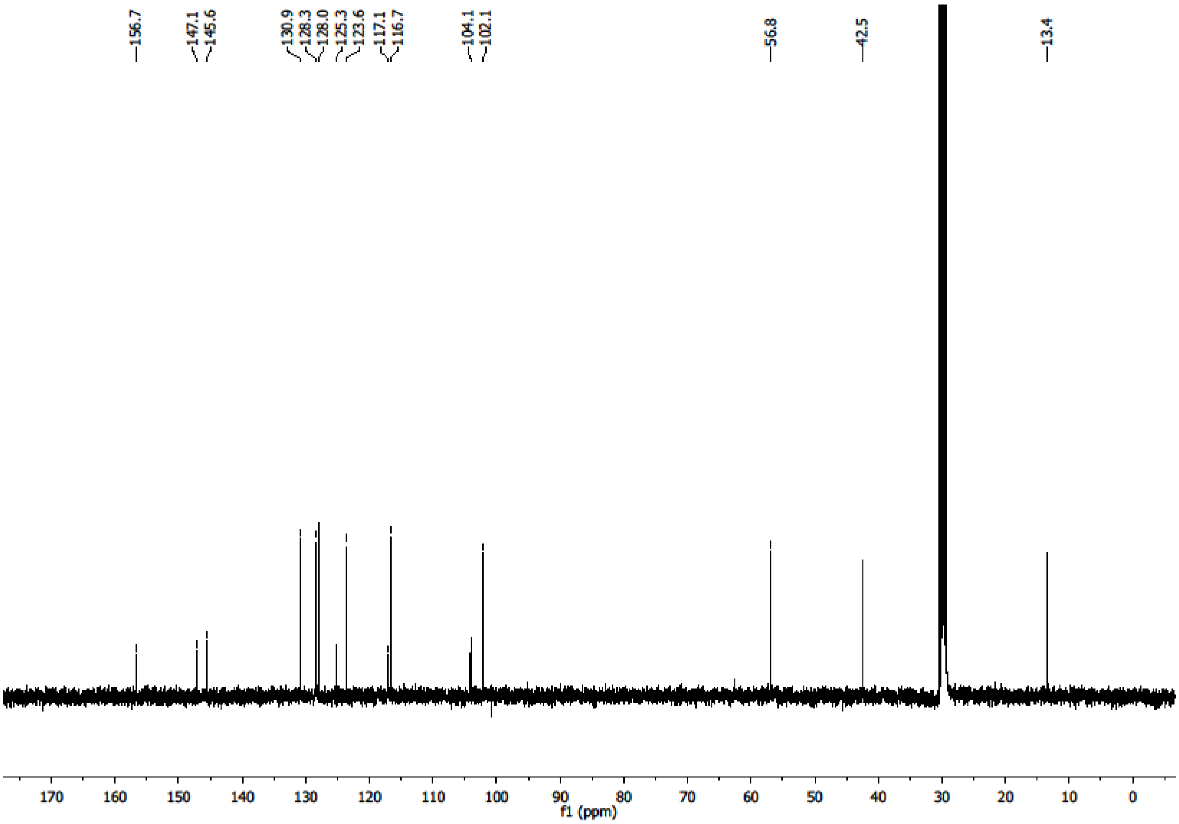
156.7
147.1 145.6
130.9 128.3 128.0 125.3 123.6 117.1 116.7
104.1 102.1
56.8
42.5
13.4
f1 (ppm)

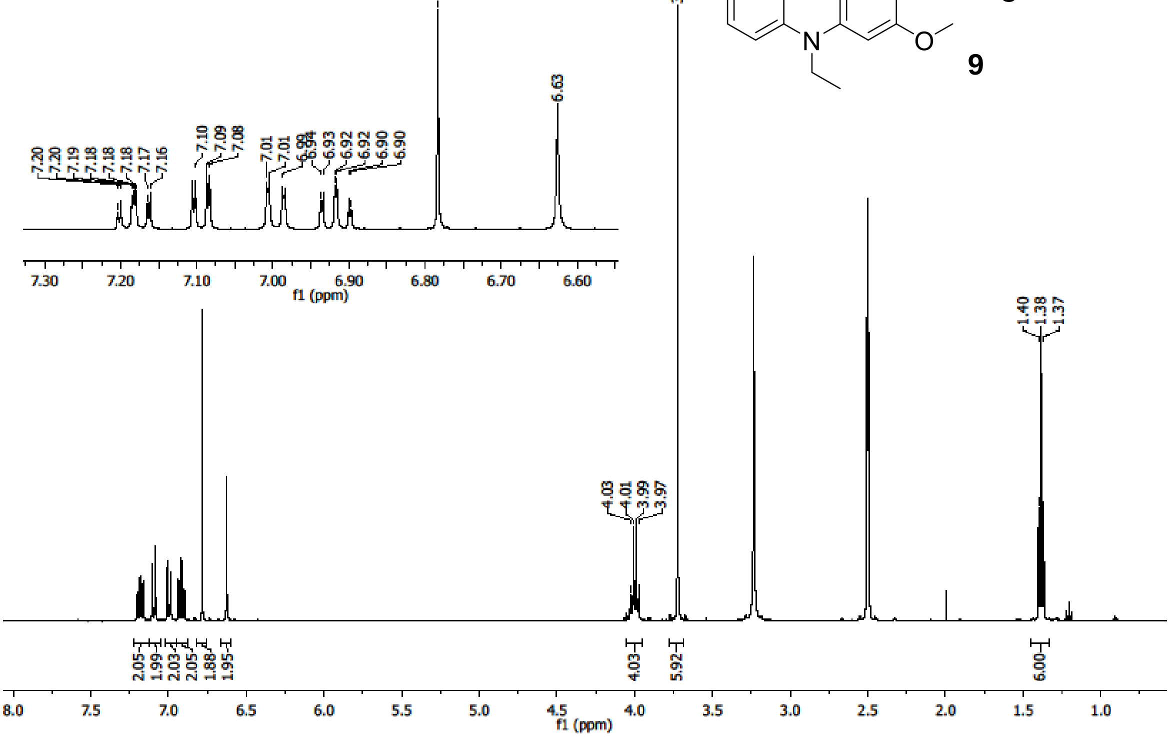
6.78
6.63
3.72
7.20 7.20 7.19 7.18 7.18 7.18 7.17 7.16
7.10 7.09 7.08
7.01 7.01 6.99 6.94 6.93 6.92 6.92 6.90 6.90 6.90
7.30 7.20 7.10 7.00 6.90 6.80 6.70 6.60
f1 (ppm)
1.40 1.38 1.37
4.03 4.01 3.99 3.97
2.05 1.99 2.03 2.05 1.88 1.95
4.03
5.92
6.00
8.0 7.5 7.0 6.5 6.0 5.5 5.0 4.5 4.0 3.5 3.0 2.5 2.0 1.5 1.0
f1 (ppm)

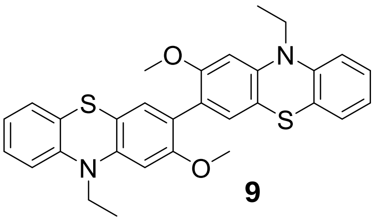
S
N
O
S
N
O

9

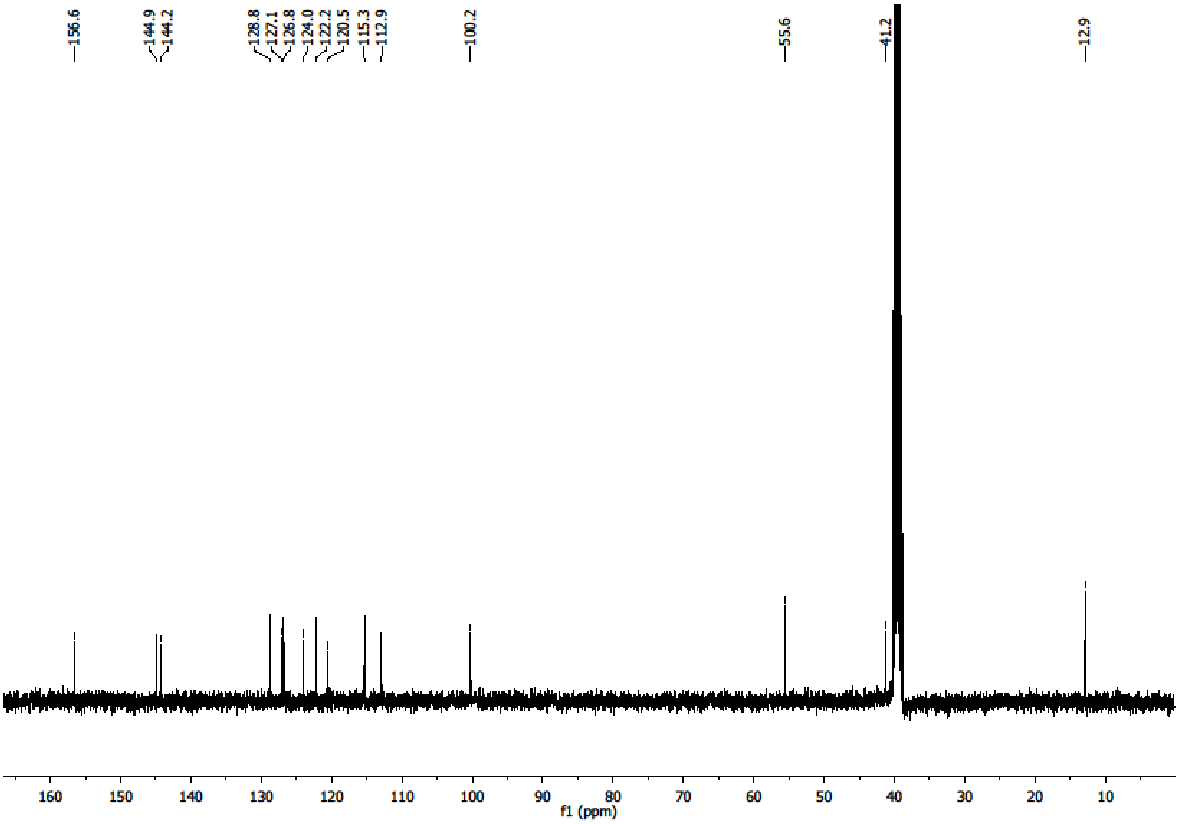
156.6
144.9
144.2
128.8
127.1
126.8
124.0
122.2
120.5
115.3
112.9
100.2
55.6
41.2
12.9
160 150 140 130 120 110 100 90 80 70 60 50 40 30 20 10
f1 (ppm)

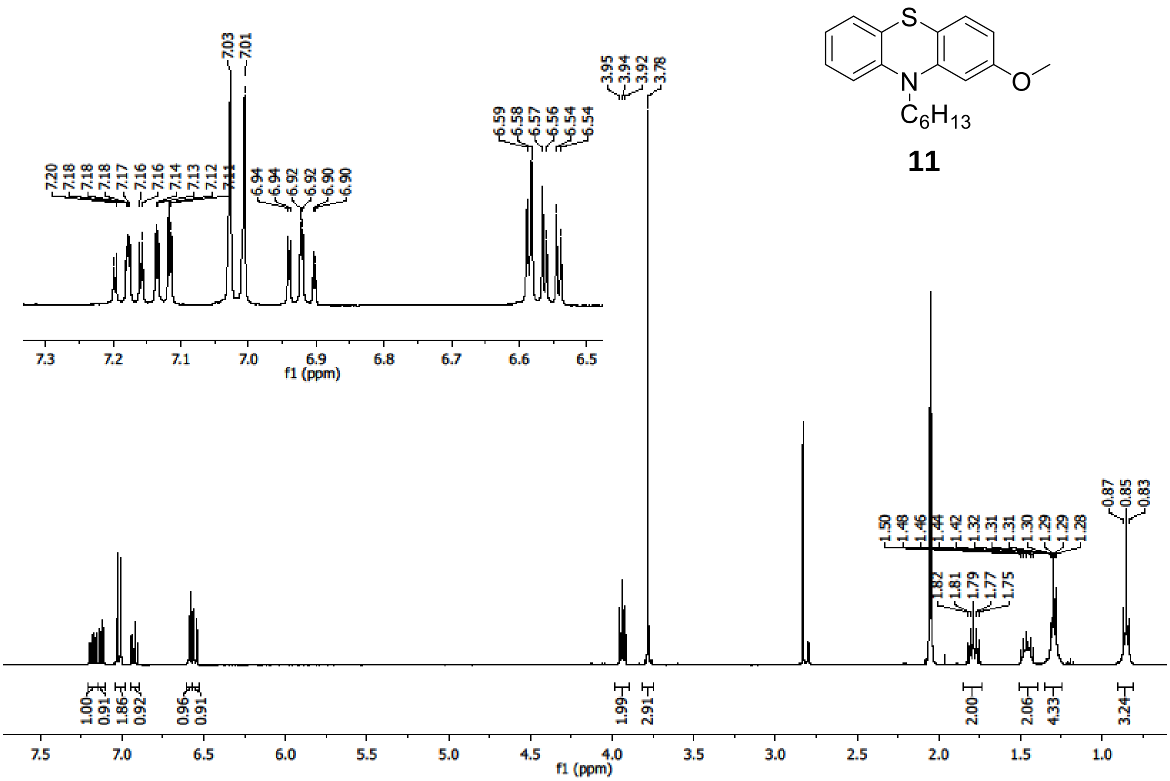
S
N
O
C6H13
11
f1 (ppm)

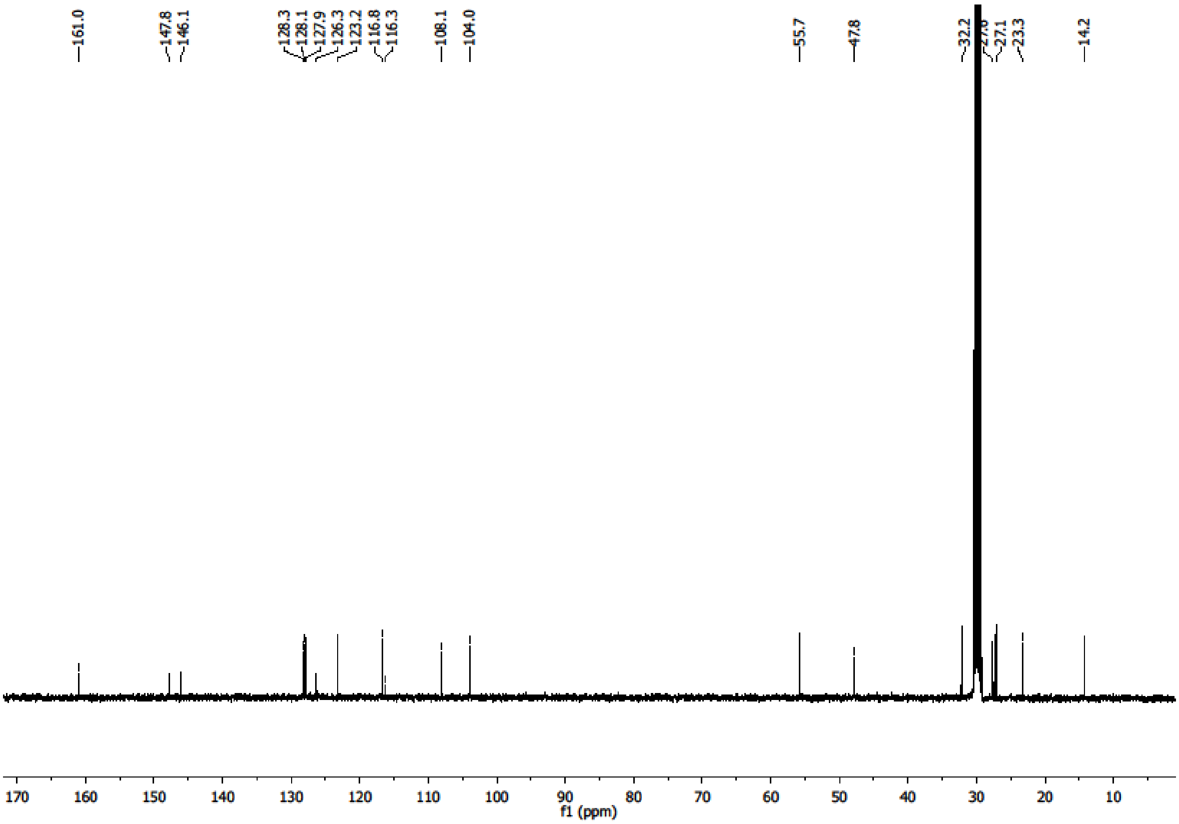
f1 (ppm)

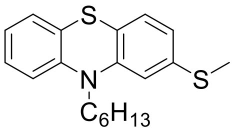

12

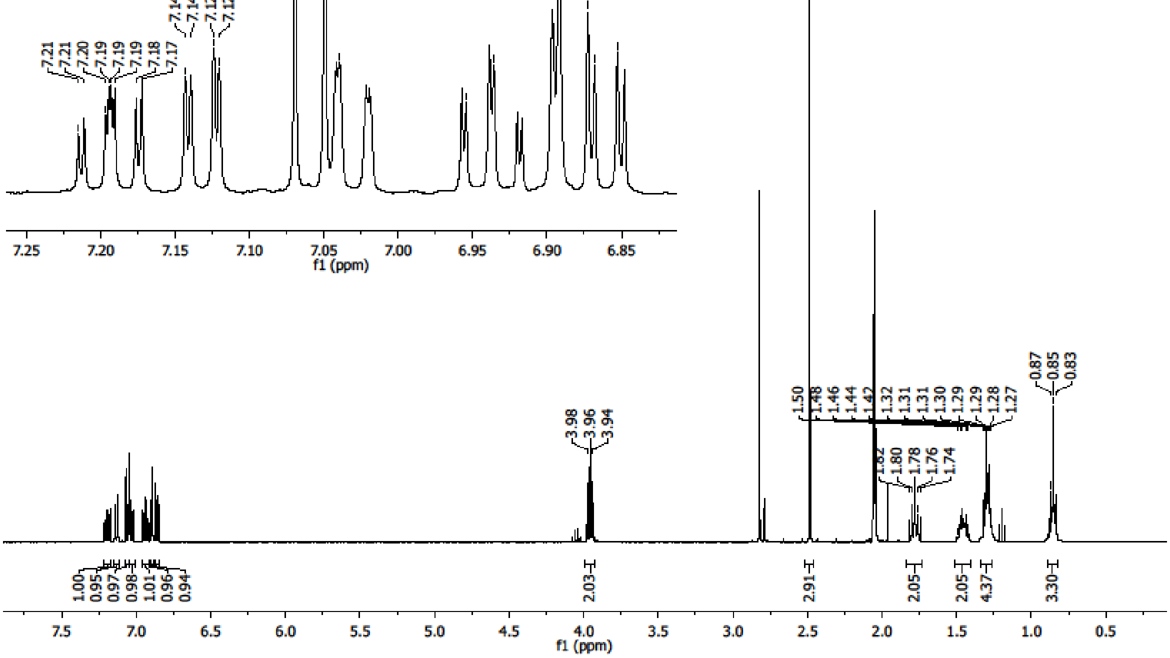

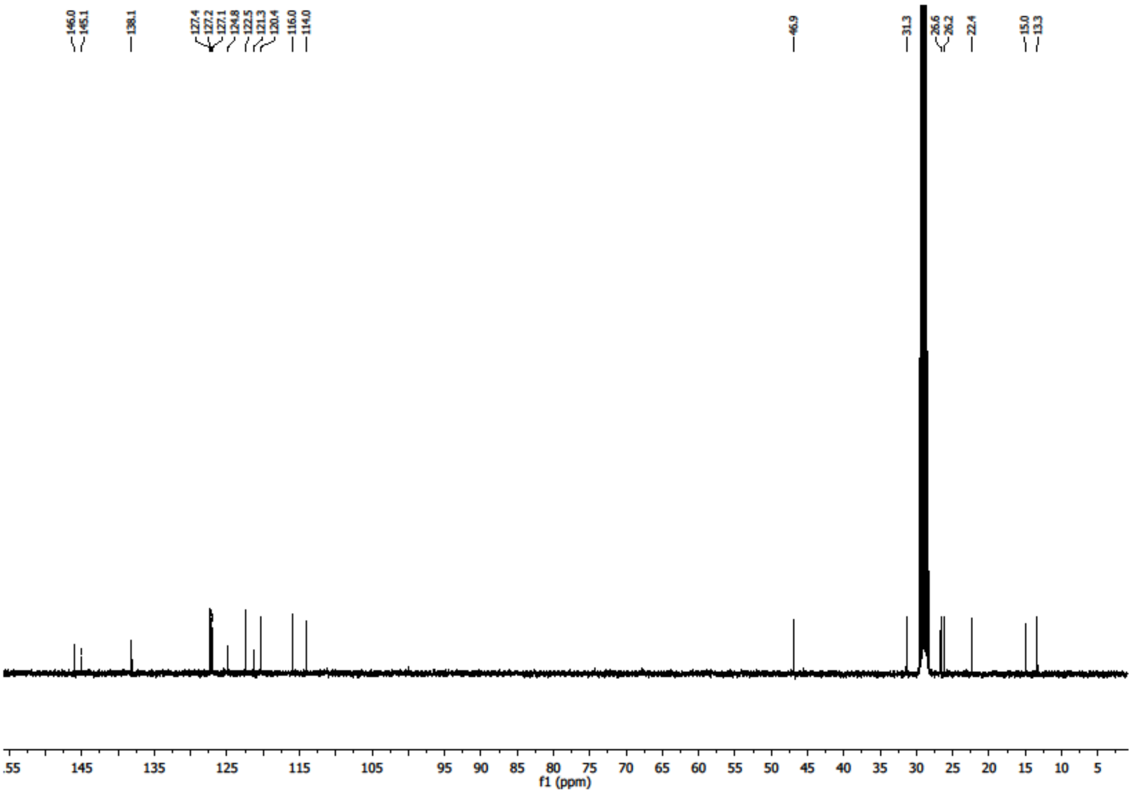

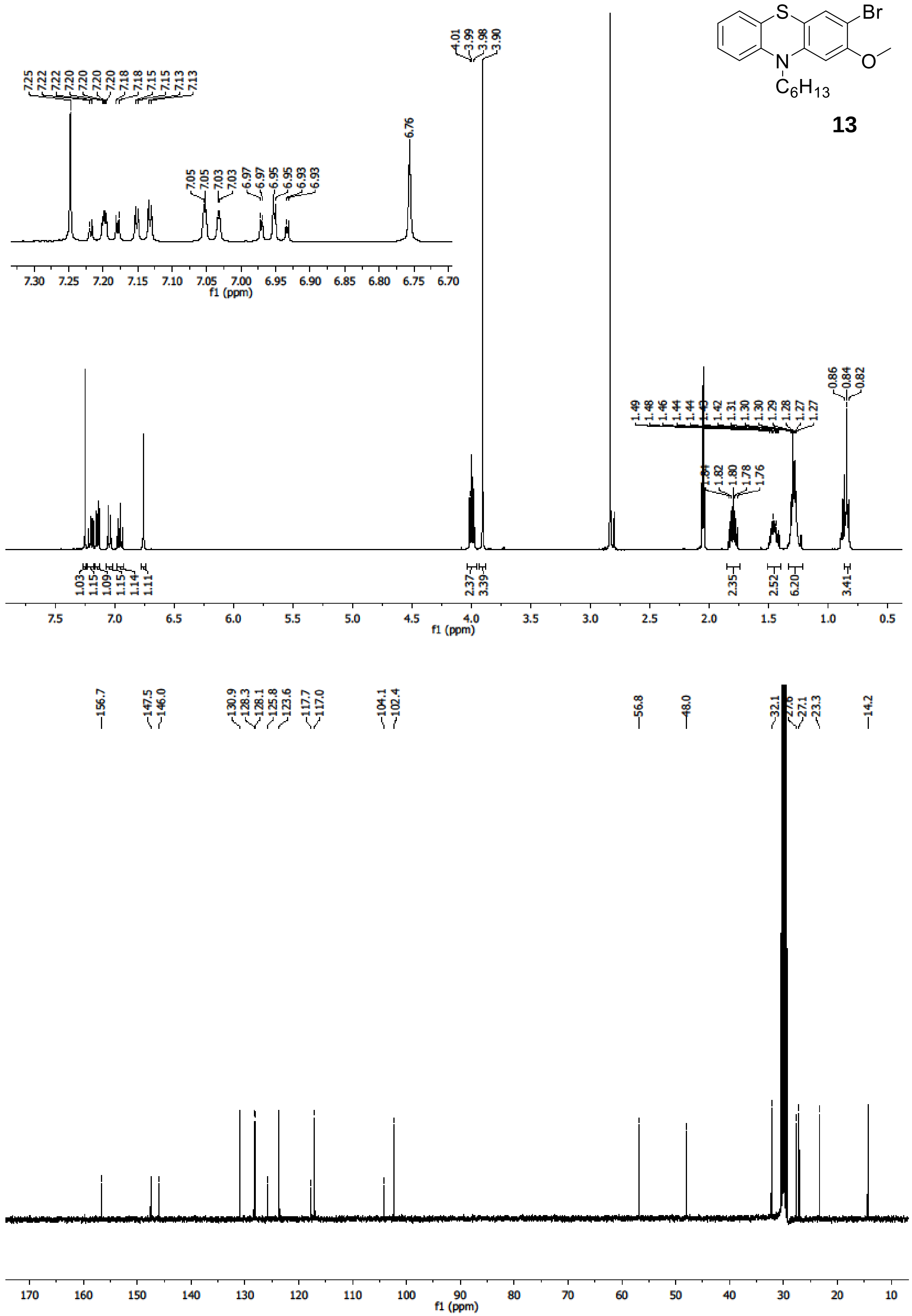

13

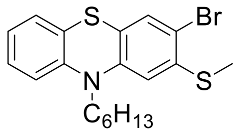
S
Br
N
S
C6H13

14

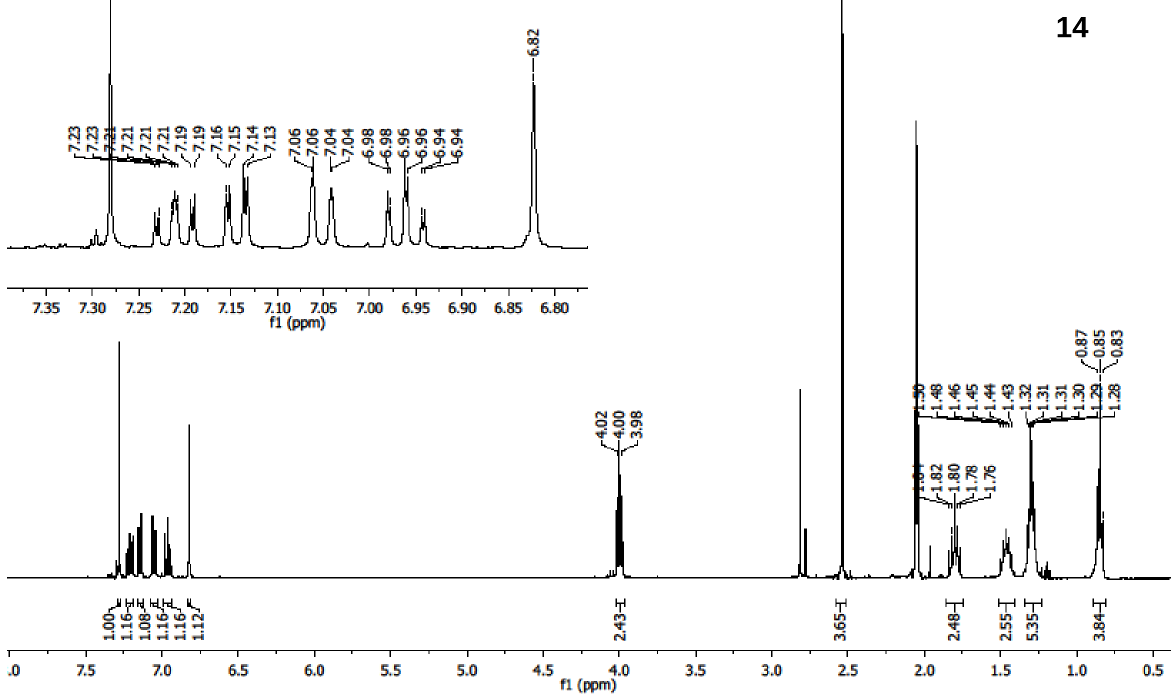
7.28
6.82
2.54
4.02
4.00
3.98
0.87
0.85
0.83
f1 (ppm)

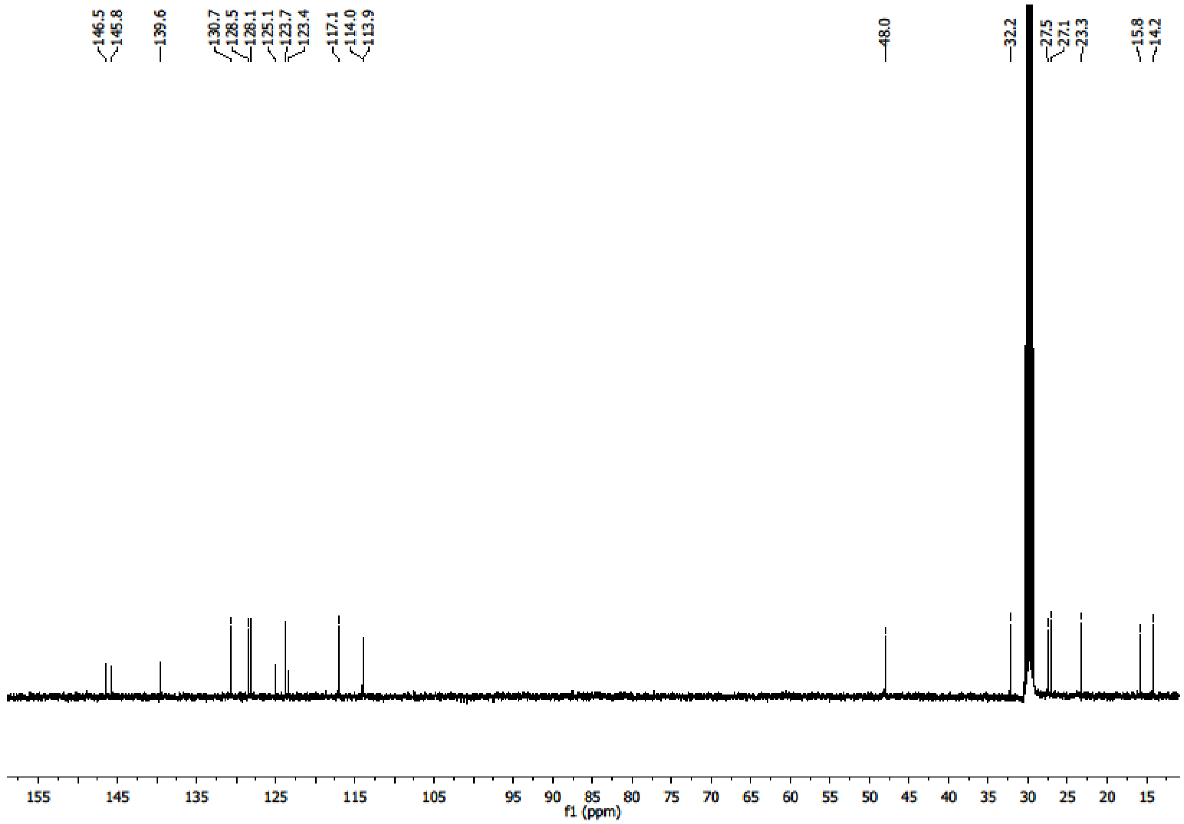
146.5
145.8
139.6
130.7
128.5
128.1
125.1
123.7
123.4
117.1
114.0
113.9
48.0
32.2
27.5
27.1
23.3
15.8
14.2
f1 (ppm)

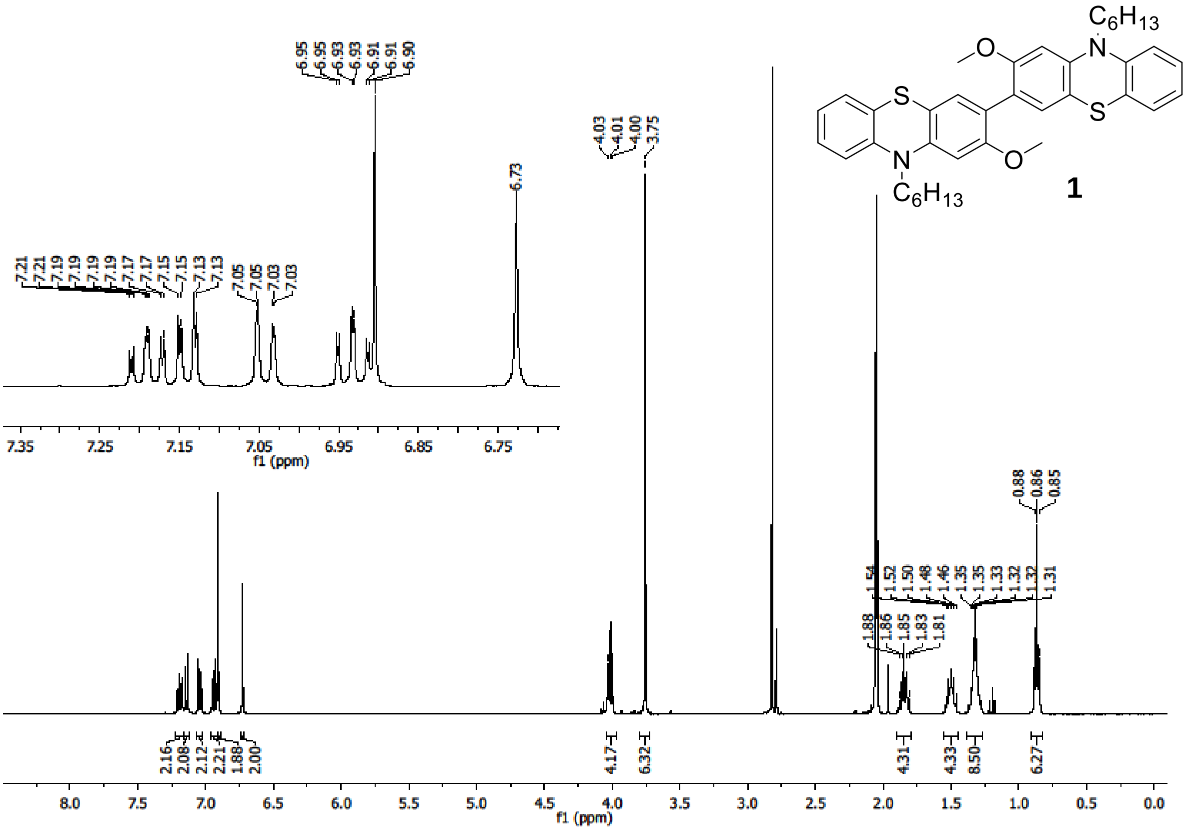
7.21 7.21 7.19 7.19 7.19 7.19 7.17 7.17 7.15 7.15 7.13 7.13 7.05 7.05 7.03 7.03
6.95 6.95 6.93 6.93 6.91 6.91 6.90
6.73
4.03 4.01 4.00 3.75
0.88 0.86 0.85
1.54 1.52 1.50 1.48 1.46 1.35 1.35 1.33 1.32 1.32 1.31
1.88 1.86 1.85 1.83 1.81
2.16 2.08 2.12 2.21 1.88 2.00 4.17 6.32 4.31 4.33 8.50 6.27
f1 (ppm)
C6H13
O
N
S
S
N
O
C6H13

1

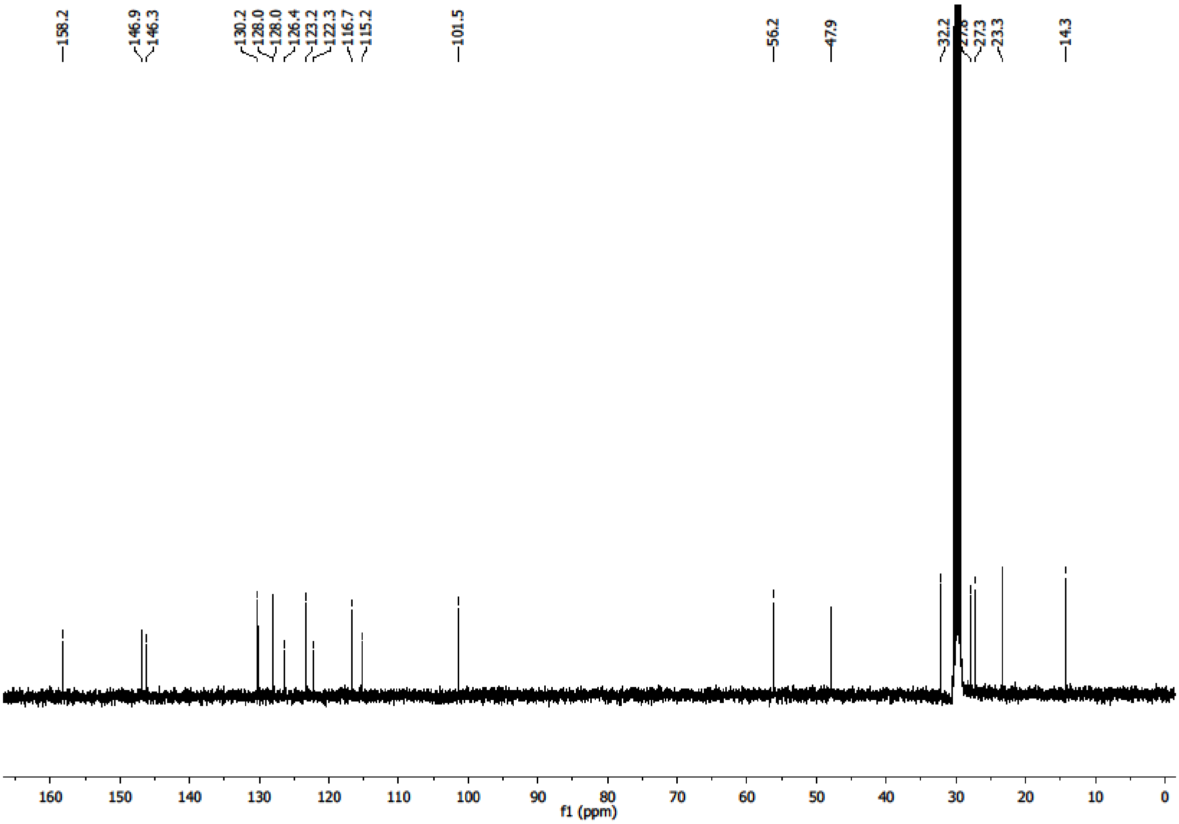
158.2
146.9 146.3
130.2 128.0 128.0 126.4 123.2 122.3 116.7 115.2
101.5
56.2
47.9
32.2 27.3 23.3
14.3
f1 (ppm)

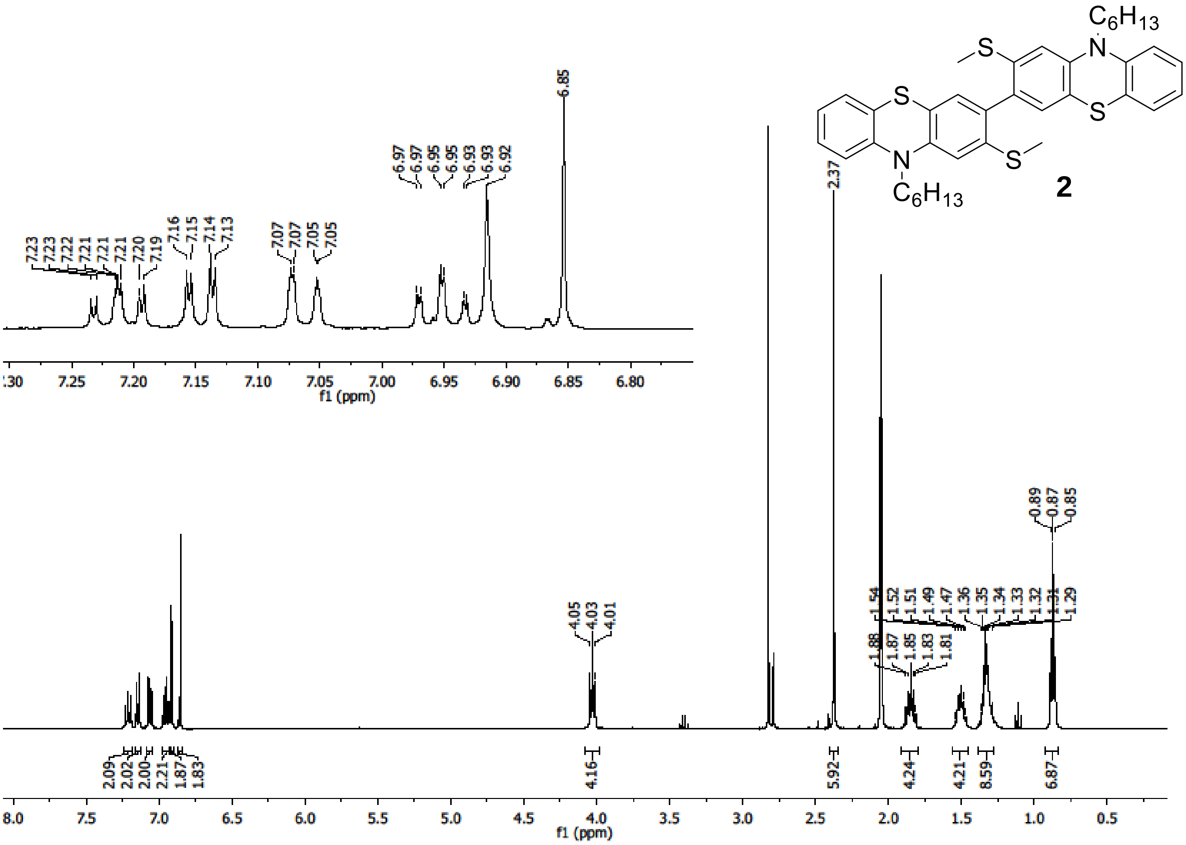
7.23 7.23 7.22 7.21 7.21 7.21 7.20 7.19 7.16 7.15 7.14 7.13 7.07 7.07 7.05 7.05 6.97 6.97 6.95 6.95 6.93 6.93 6.92 6.85
f1 (ppm)
C_6H_{13}
S
N
S
S
N
C_6H_{13}
S
2
4.05 4.03 4.01
2.37
1.88 1.87 1.85 1.83 1.81
1.54 1.52 1.51 1.49 1.47 1.36 1.35 1.34 1.33 1.32 1.31 1.29
0.89 0.87 0.85
2.09 2.02 2.00 2.21 1.87 1.83 4.16 5.92 4.24 4.21 8.59 6.87
f1 (ppm)

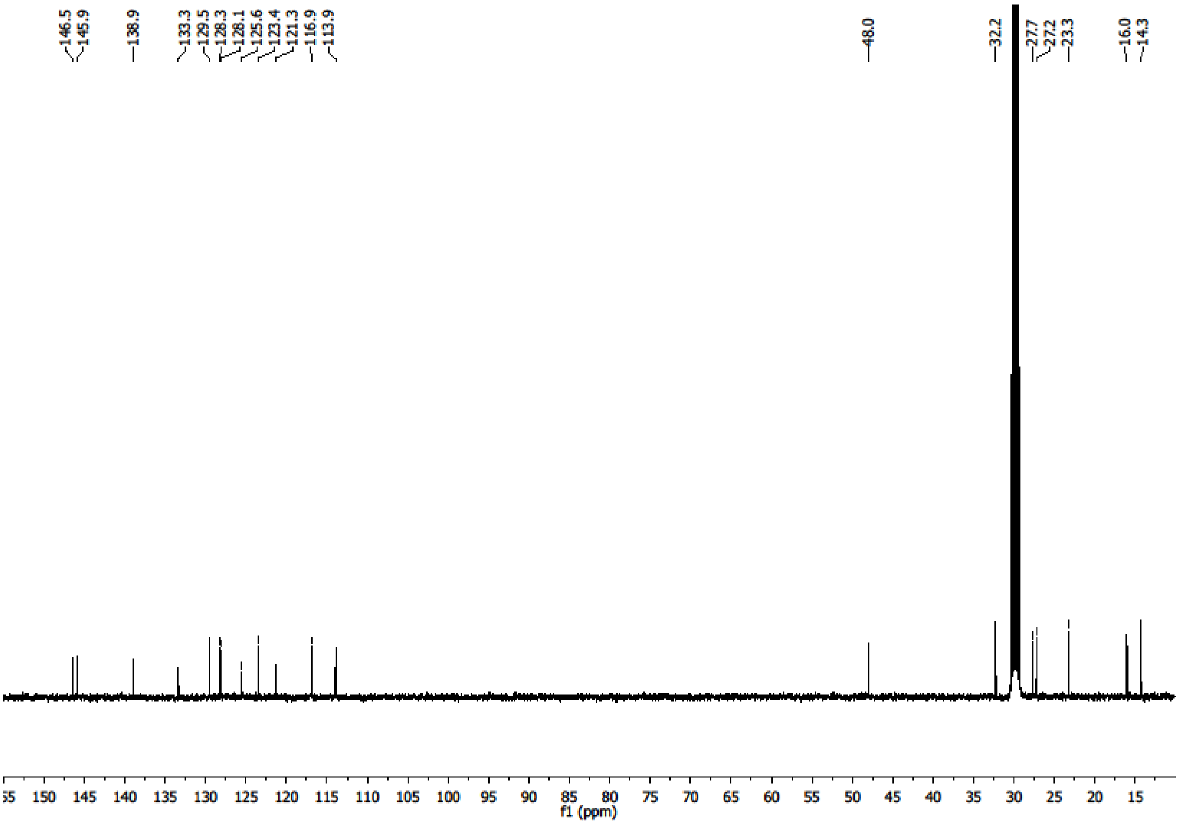
146.5 145.9 138.9 133.3 129.5 128.3 128.1 125.6 123.4 121.3 116.9 113.9 48.0 32.2 27.7 27.2 23.3 16.0 14.3
f1 (ppm)

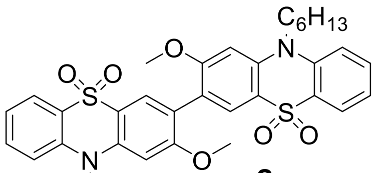

C_6H_{13}
N
O
S
O
C_6H_{13}

3

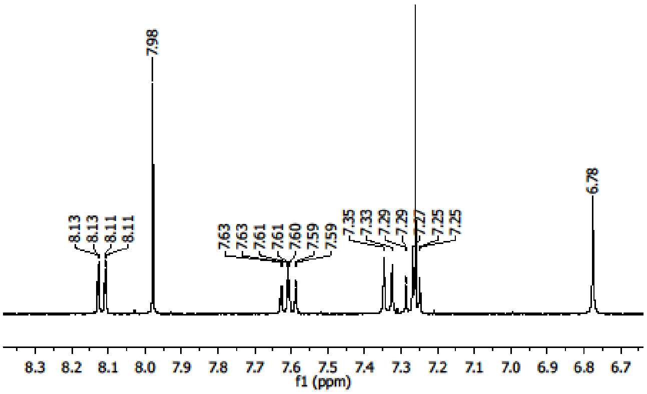

8.13 8.13 8.11 8.11 7.98 7.63 7.63 7.61 7.61 7.60 7.59 7.59 7.35 7.33 7.29 7.29 7.27 7.25 7.25 6.78
f1 (ppm)

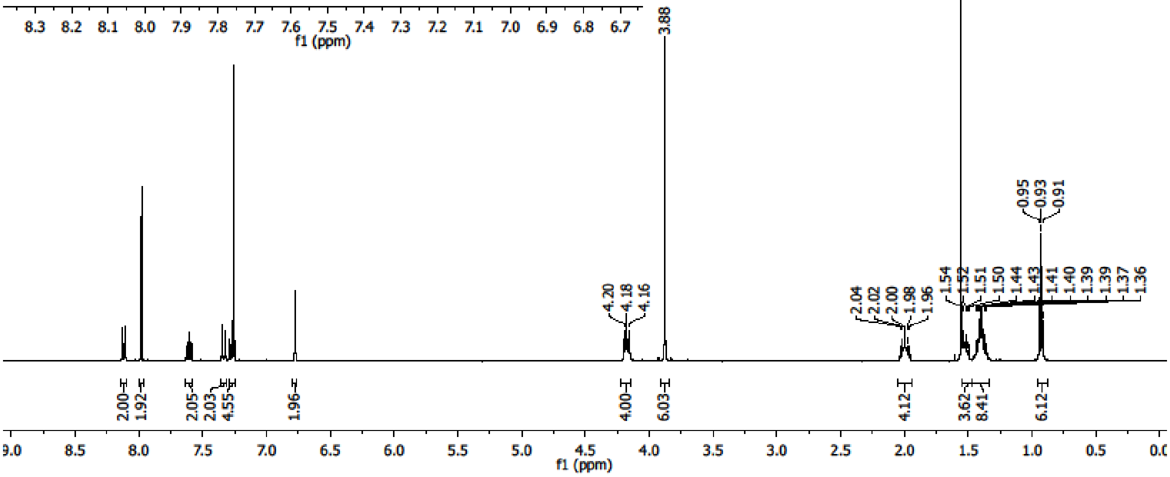

3.88
4.20 4.18 4.16
2.04 2.02 2.00 1.98 1.96
1.54 1.52 1.51 1.50 1.44 1.43 1.41 1.40 1.39 1.39 1.37 1.36
0.95 0.93 0.91
2.00 1.92 2.05 2.03 4.55 1.96 4.00 6.03 4.12 3.62 8.41 6.12
f1 (ppm)

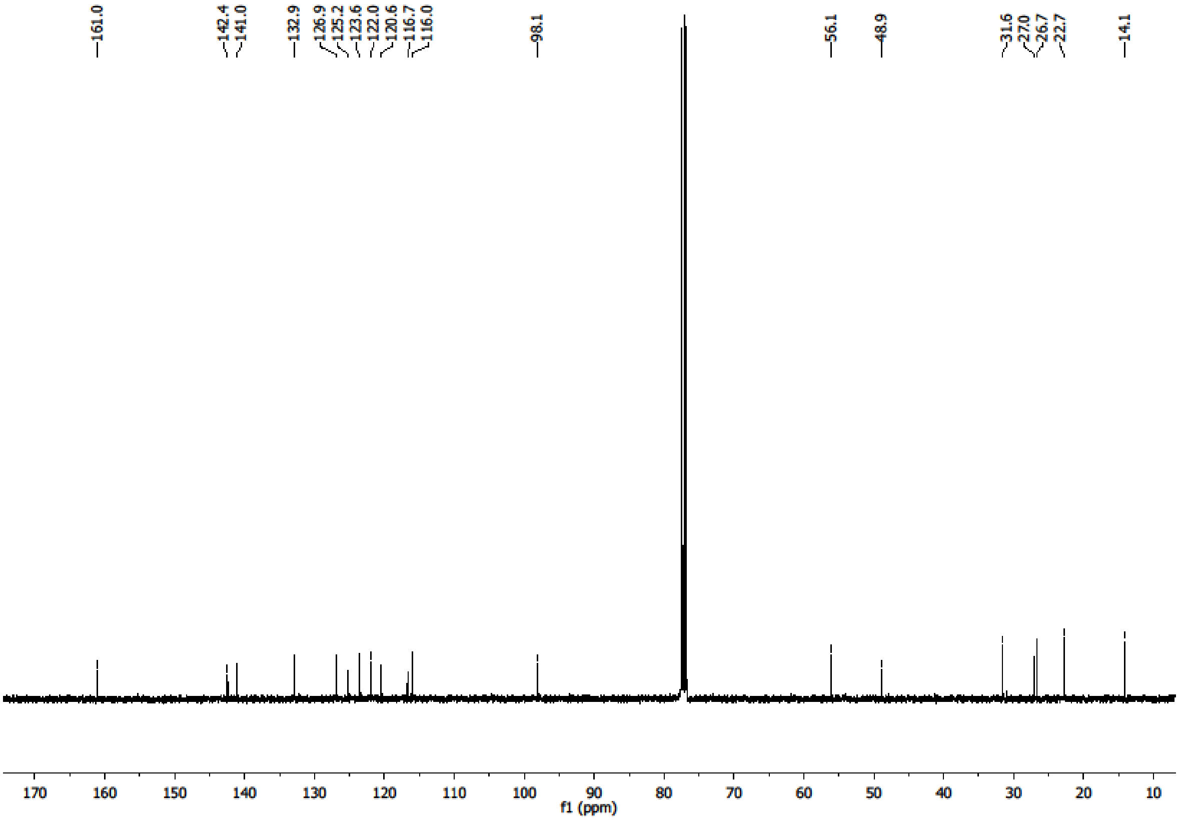

161.0 142.4 141.0 132.9 126.9 125.2 123.6 122.0 120.6 116.7 116.0 98.1 56.1 48.9 31.6 27.0 26.7 22.7 14.1
f1 (ppm)

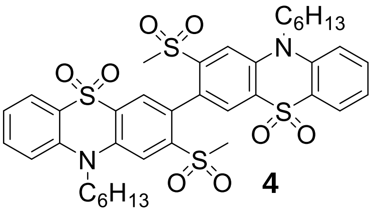
O O
S
O O
S
N
C6H13
N
C6H13
S
O O
S
O O

4

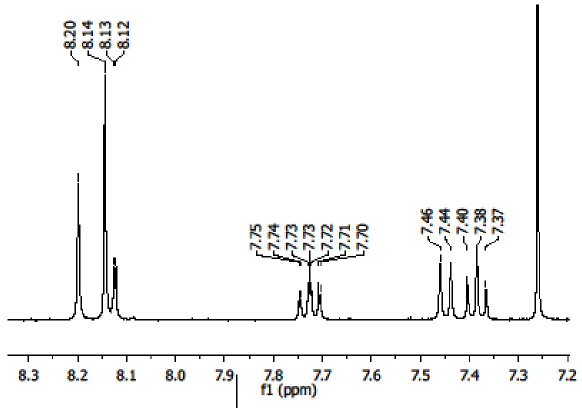
8.20
8.14
8.13
8.12
7.75
7.74
7.73
7.73
7.72
7.71
7.70
7.46
7.44
7.40
7.38
7.37
f1 (ppm)

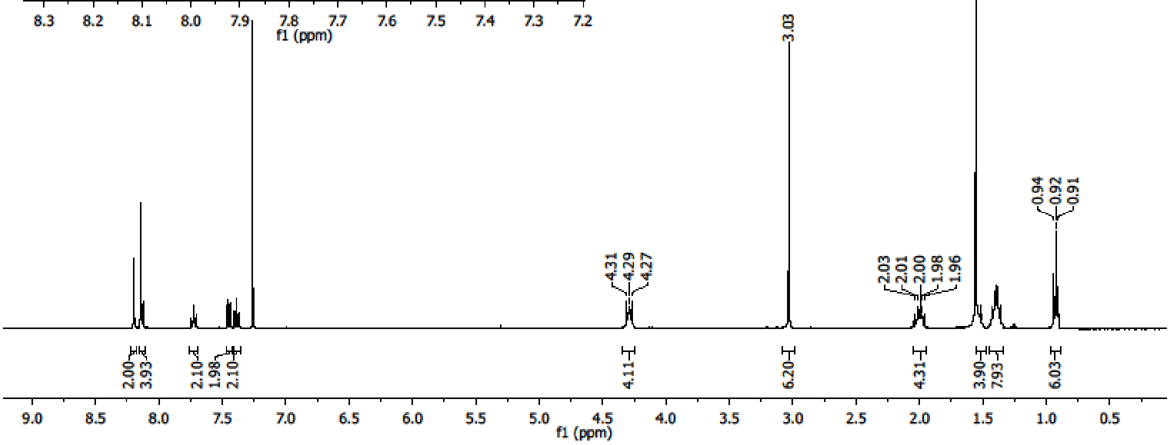
3.03
4.31
4.29
4.27
2.03
2.01
2.00
1.98
1.96
1.56
1.52
1.50
1.43
1.42
1.41
1.40
1.39
1.38
1.36
0.94
0.92
0.91
2.00
3.93
2.10
1.98
2.10
4.11
6.20
4.31
3.90
7.93
6.03
f1 (ppm)

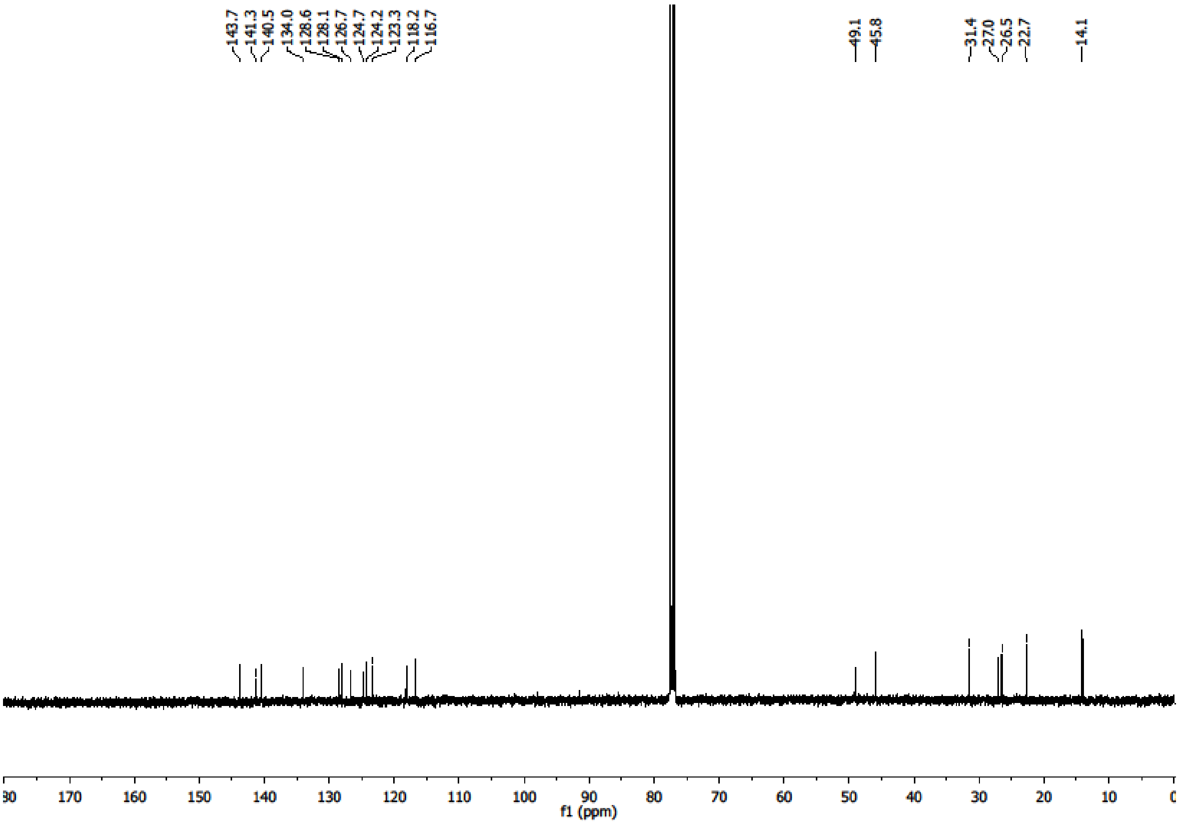
143.7
141.3
140.5
134.0
128.6
128.1
126.7
124.7
124.2
123.3
118.2
116.7
49.1
45.8
31.4
27.0
26.5
22.7
14.1
f1 (ppm)

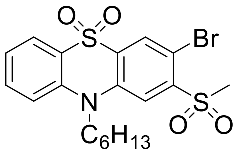

Br
N
S
O
C_6H_{13}

15

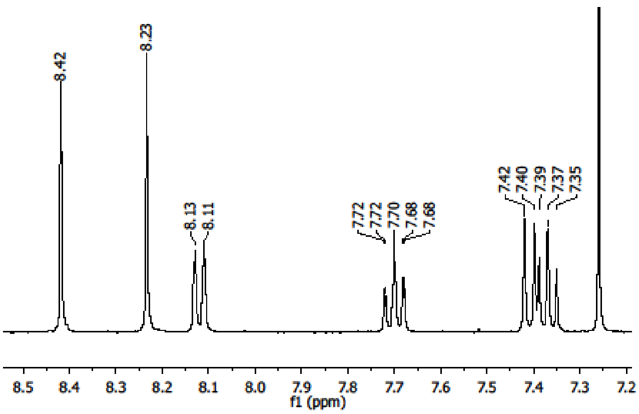

8.42
8.23
8.13
8.11
7.72
7.72
7.70
7.68
7.68
7.42
7.40
7.39
7.37
7.35
f1 (ppm)

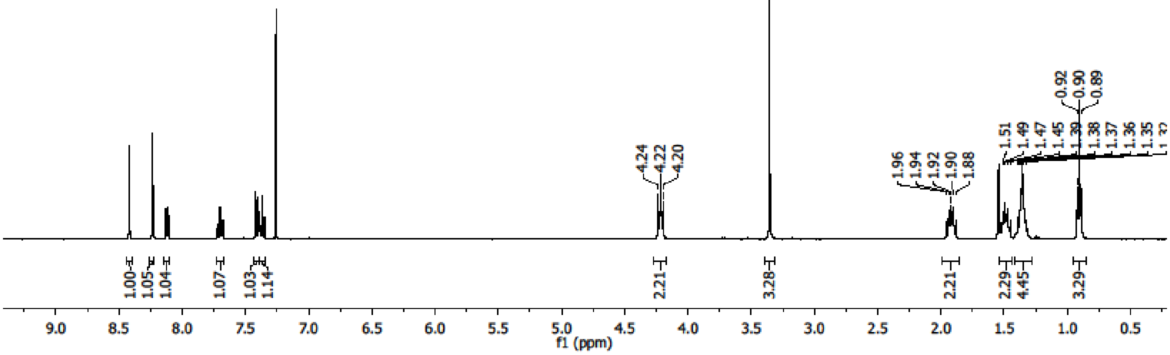

4.24
4.22
4.20
3.36
1.96
1.94
1.92
1.90
1.88
0.92
0.90
0.89
1.00
1.05
1.04
1.07
1.03
1.14
2.21
3.28
2.21
2.29
4.45
3.29
f1 (ppm)

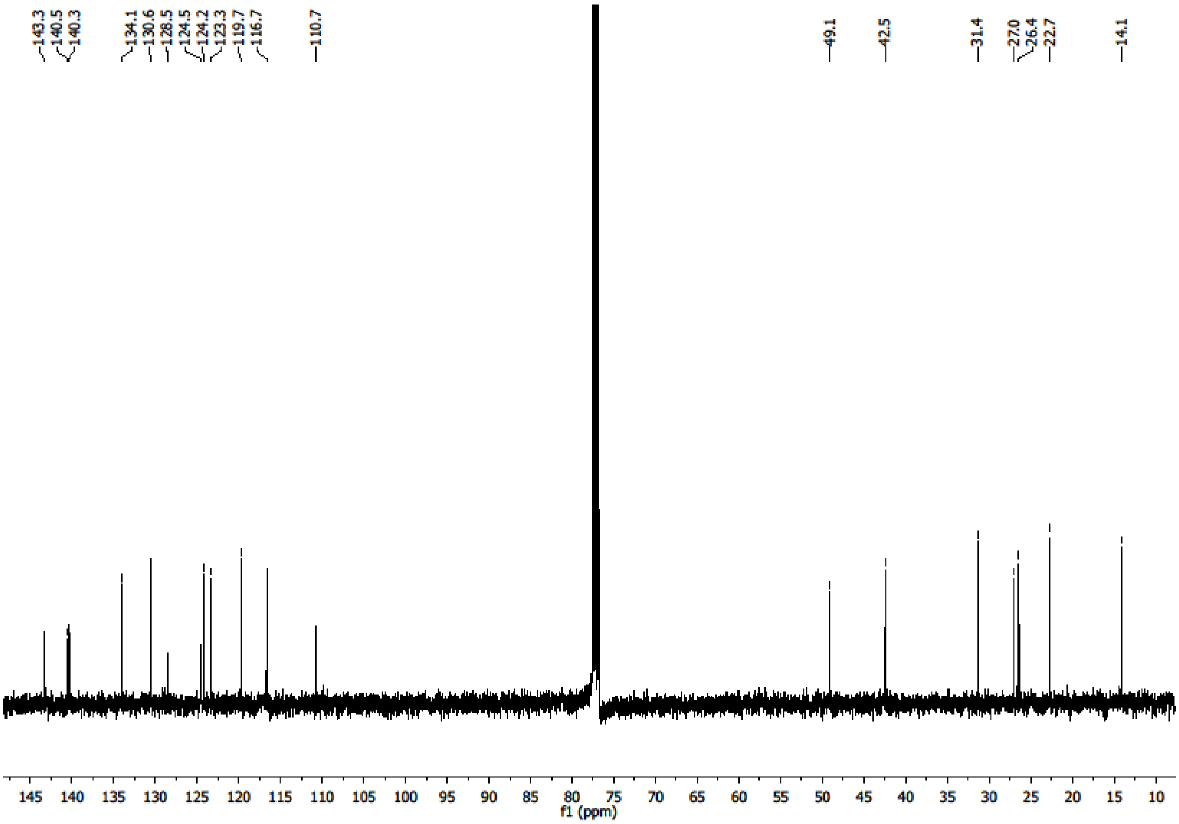

143.3
140.5
140.3
134.1
130.6
128.5
124.5
124.2
123.3
119.7
116.7
110.7
49.1
42.5
31.4
27.0
26.4
22.7
14.1
f1 (ppm)

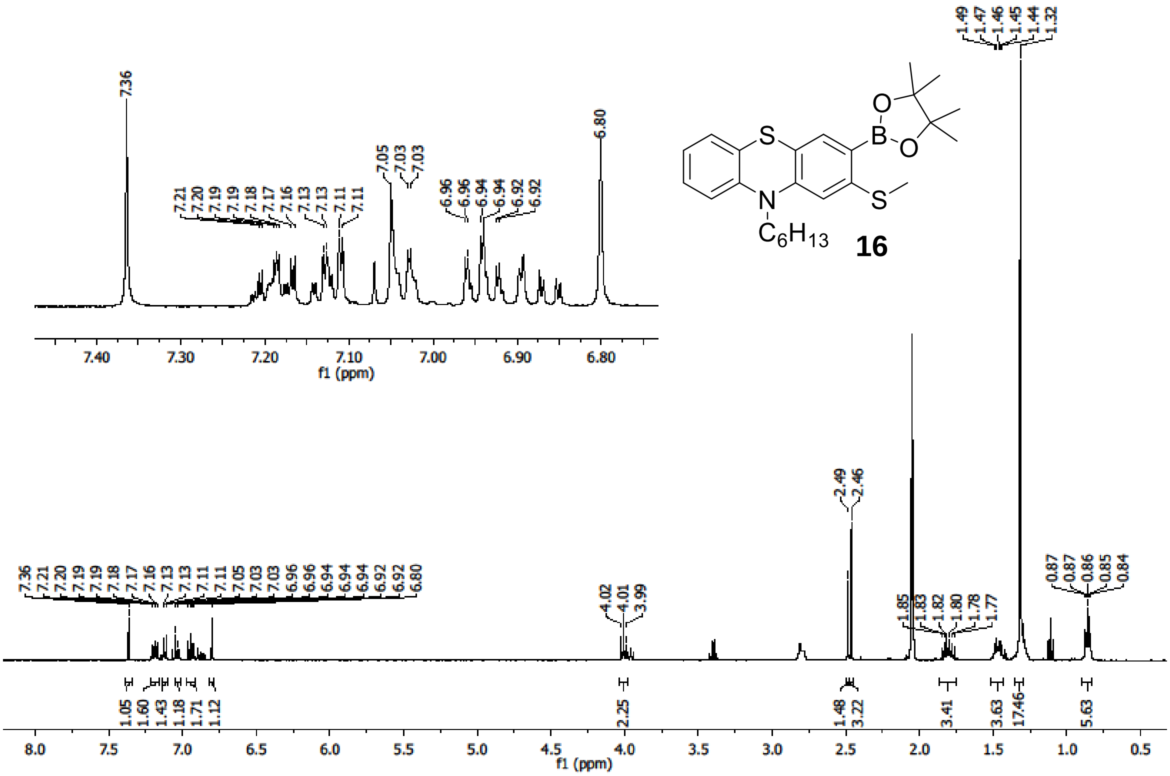
16
f1 (ppm)

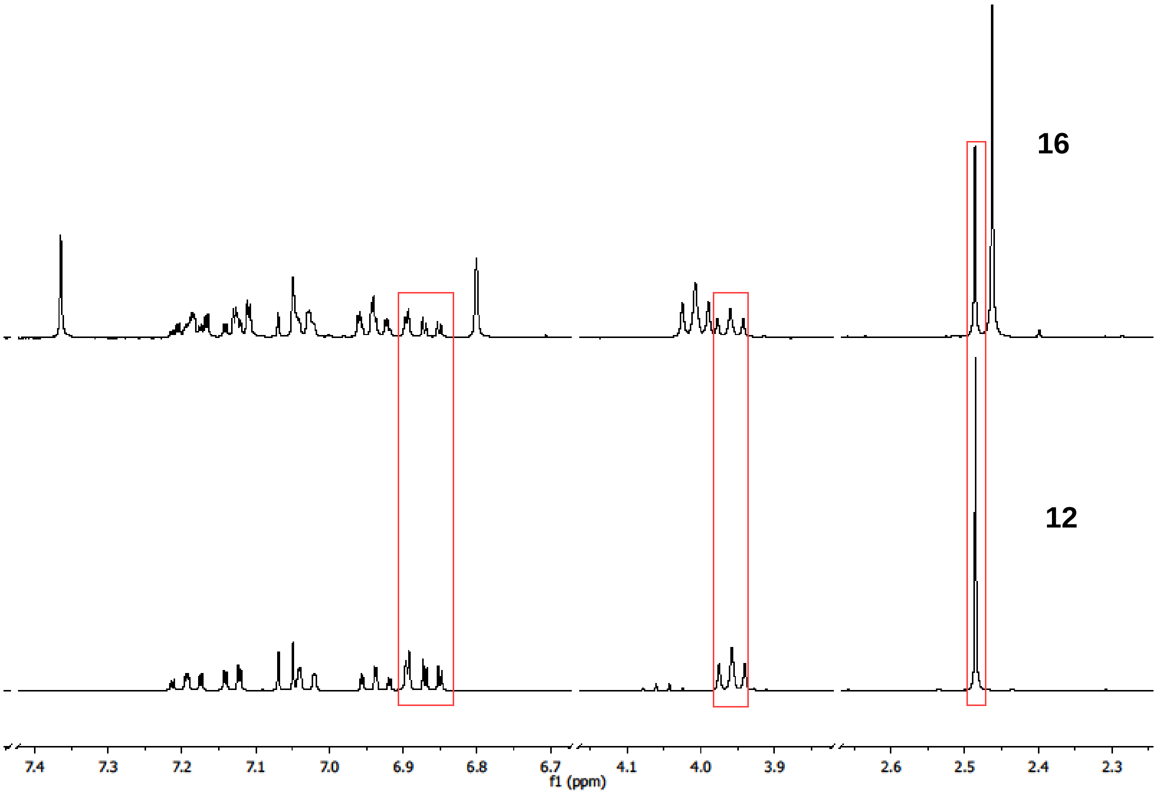
16
12
f1 (ppm)

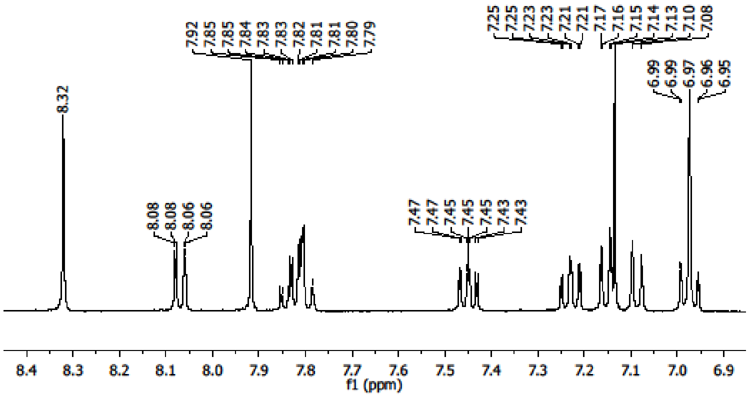

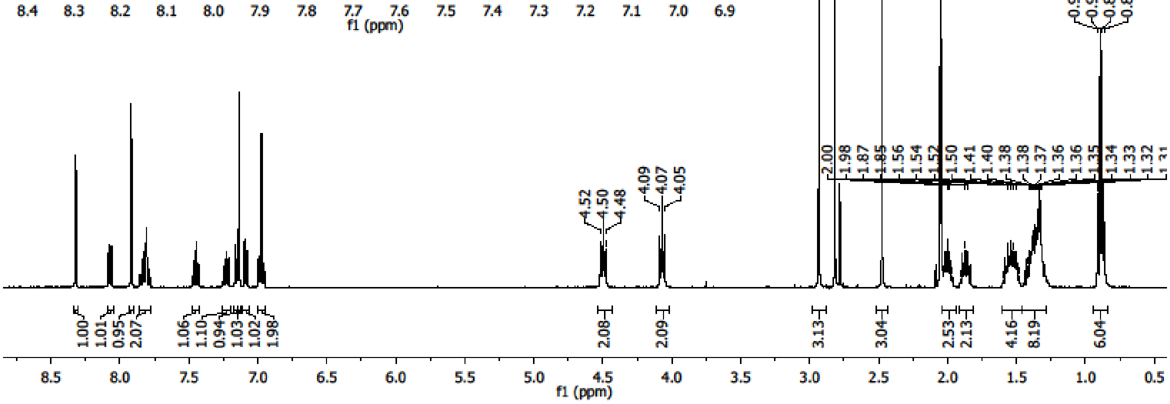

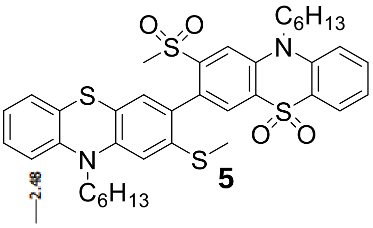

5

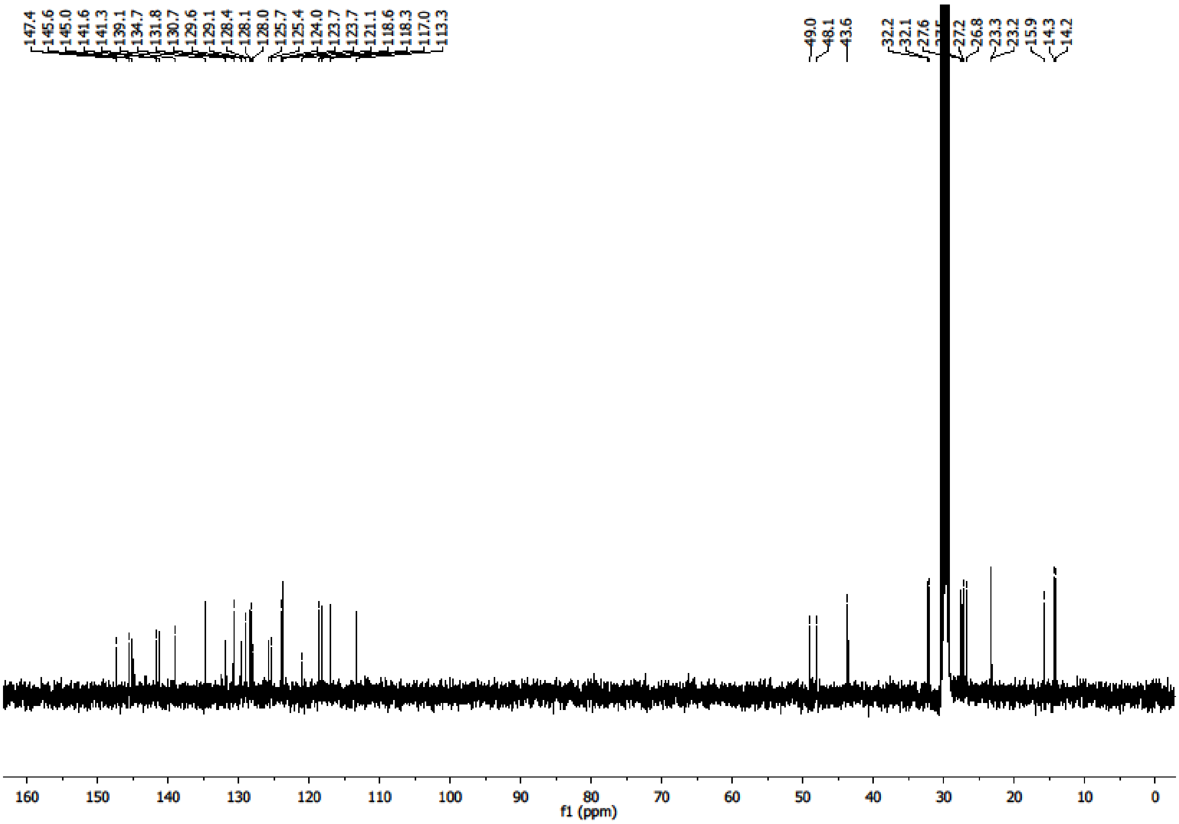

4. X-ray crystallography

X-ray diffraction experiments were performed on a Bruker D8 Venture 3-circle diffractometer with a PHOTON 100 CMOS area detector, using Mo-*Kα* or Cu-*Kα* radiation from Incoatec IµS microsources with focussing mirrors. Crystals were cooled using a Cryostream (Oxford Cryosystems) open-flow N_2 gas cryostat. The data were processed using APEX3 v.2016.1-0 and reflection intensities integrated using SAINT v8.38A software (Bruker AXS, 2016). For **4** and **5** the data were corrected for absorption by numerical integration based on crystal face-indexing; for the other compounds by semi-empirical method based on Laue equivalents and multiple scans, using SADABS 2014/5 program.[3] The structures were solved by direct methods using SHELXS 2013/1 software[4] and refined by full-matrix least squares using SHELXL 2018/3 software[5] on OLEX2 platform.[6] Compound **2** undergoes a reversible phase transition below 160 K, **3** below 200 K and **4** between 220 and 200 K; in each case only the higher-temperature polymorph was characterized. Compound **3** crystallized as non-merohedral twins with the twin law (-1 0 0 0 -1 0 0.156 0 1). In Fig S4.1-S4.9 C atoms are shown grey, N blue, O red, S yellow; H atoms are white (when shown) or omitted. Thermal ellipsoids are drawn at the 50% probability level.

Table S1 Crystal data and experimental details

**Compound**	**1**	**2**	**3**	**4**	**5**	**9**
CCDC dep.no	1888002	1888003	1888004	1888022	1888005	1888006
Formula	$C_{38}H_{44}N_2O_2S_2$	$C_{38}H_{44}N_2S_4$	$C_{38}H_{44}N_2O_6S_2$ ·$CDCl_3$	$C_{38}H_{44}N_2O_8S_4$	$C_{38}H_{44}N_2O_4S_4$	$C_{30}H_{28}N_2O_2S_2$
$D_{calc.}$/ g cm^{-3}	1.237	1.251	1.298	1.359	1.323	1.383
μ/mm^{-1}	0.20	0.30	0.37	2.72	2.75	0.25
F.w.	624.87	656.99	809.24	784.99	720.99	512.66
T/K	120	180	200	220	120	120
Crystal System	orthorhombic	monoclinic	monoclinic	monoclinic	monoclinic	orthorhombic
Space Group	$P2_12_12$ (no.18)	$I2/a$ (no.15)	$C2/c$ (no. 15)	$P2_1/n$ (no.14)	$P2_1/c$ (no.14)	$Fdd2$ (no.43)
a/Å	17.3991(10)	17.9715(8)	34.621(2)	13.6754(4)	12.8446(3)	22.3337(13)
b/Å	8.6325(5)	14.0355(6)	9.3750(5)	10.9130(3)	11.4013(2)	27.6872(16)
c/Å	11.1700(6)	27.8638(15)	25.6696(15)	26.2247(8)	25.2420(5)	7.9651(5)
β/°	90	96.8146(15)	96.049(2)	101.2974(13)	101.2348(9)	90
V/Å^3	1677.7(2)	6978.7(8)	8285.3(13)	3837.9(2)	3625.74(16)	4925.3(7)
Z	2	8	8	4	4	8
λ/Å	0.71073	0.71073	0.71073	1.54184	1.54184	0.71073
Radiation type	Mo-K_α	Mo-K_α	Mo-K_α	Cu-K_α	Cu-K_α	Mo-K_α
$2\theta_{max}$/°	63	50	55	151.3	151.5	60.4
Reflections total	42733	54365	69698	39876	40190	27288
unique	6069	6174	9582	7704	7239	3633
with $I > 2\sigma(I)$	5327	4430	8141	6247	5567	3401
R_{int}	0.046	0.053	0.039	0.045	0.070	0.038
Parameters	203, 0	421, 363	562, 480	523, 404	441, 369	167, 1
$\Delta\rho_{max,min}$, Å	0.35, -0.23	0.58, -0.38	0.66, -0.51	0.50, -0.40	0.42, -0.38	0.30, -0.25
Goodness of fit	1.065	1.035	1.034	1.039	1.014	1.055
R_1, wR_2 (all data)	0.050, 0.095	0.085, 0.164	0.086, 0.216	0.062, 0.130	0.063, 0.101	0.036, 0.081
R_1, wR_2 [$I > 2\sigma(I)$]	0.039, 0.091	0.057, 0.144	0.075, 0.205	0.049, 0.121	0.041, 0.091	0.032, 0.078

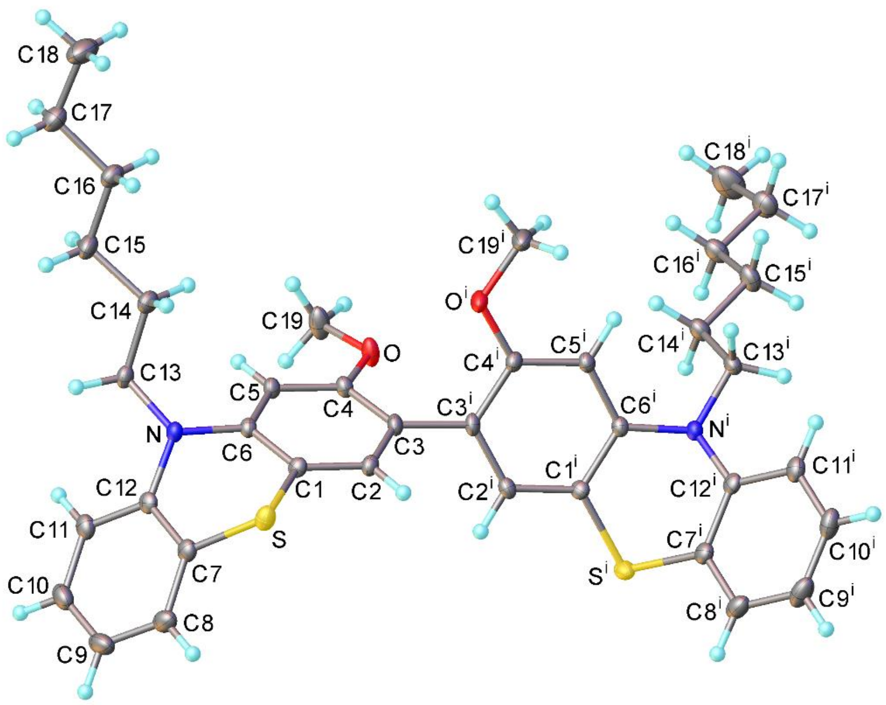

Figure S4.1 X-ray molecular structure of **1**. Primed atoms are generated by the twofold axis.

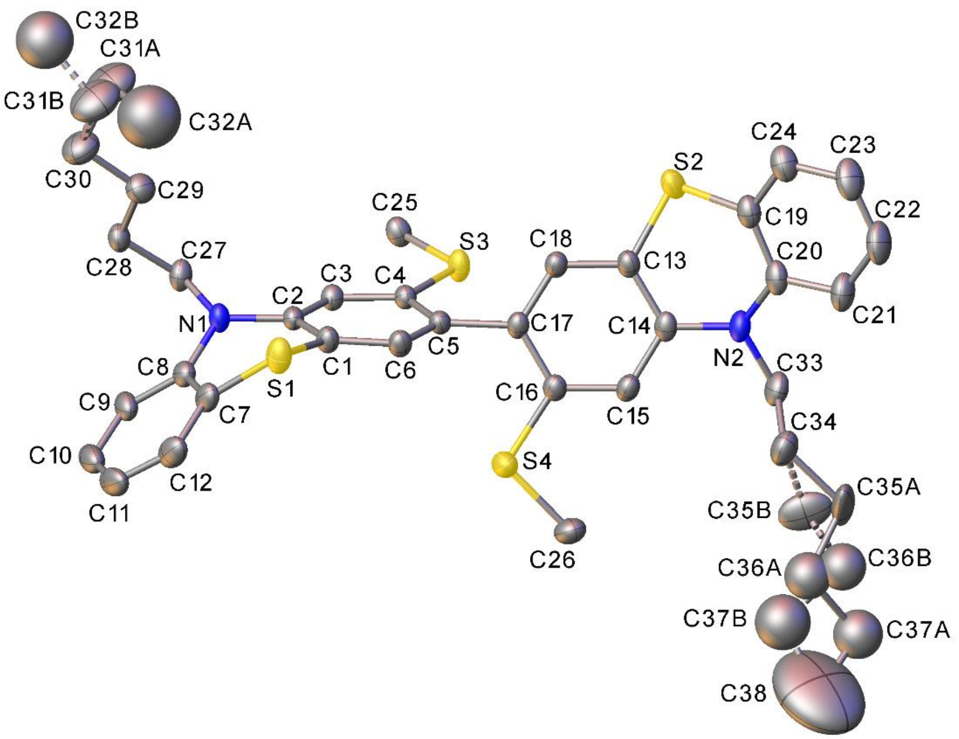

Figure S4.2. X-ray molecular structure of **2**, showing the disorder of n-hexyl chains. H atoms are omitted for clarity.

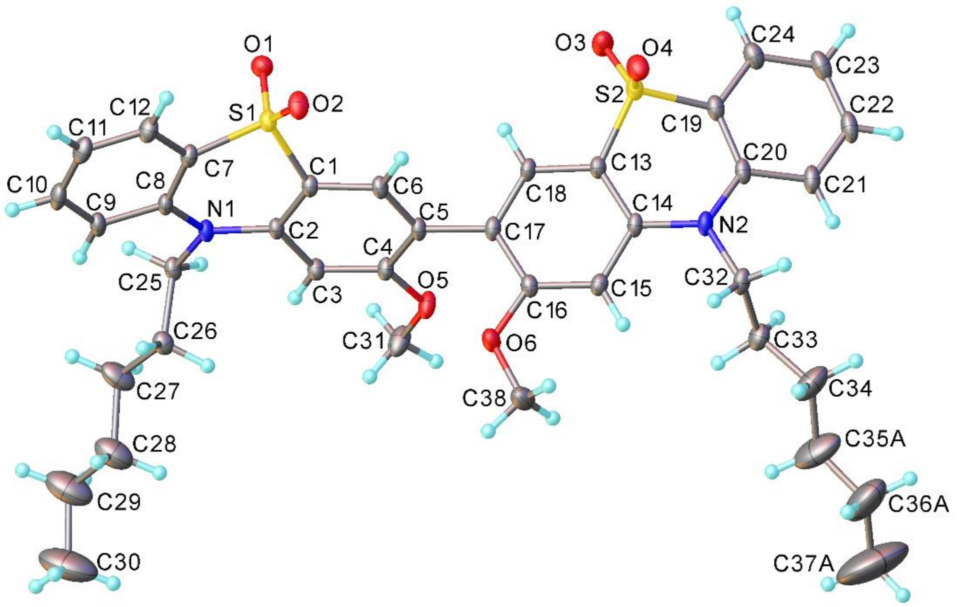

Figure S4.3 X-ray molecular structure of **3**. Minor positions of the disordered n-hexyl chains and the disordered d-chloroform molecules of crystallisation are omitted.

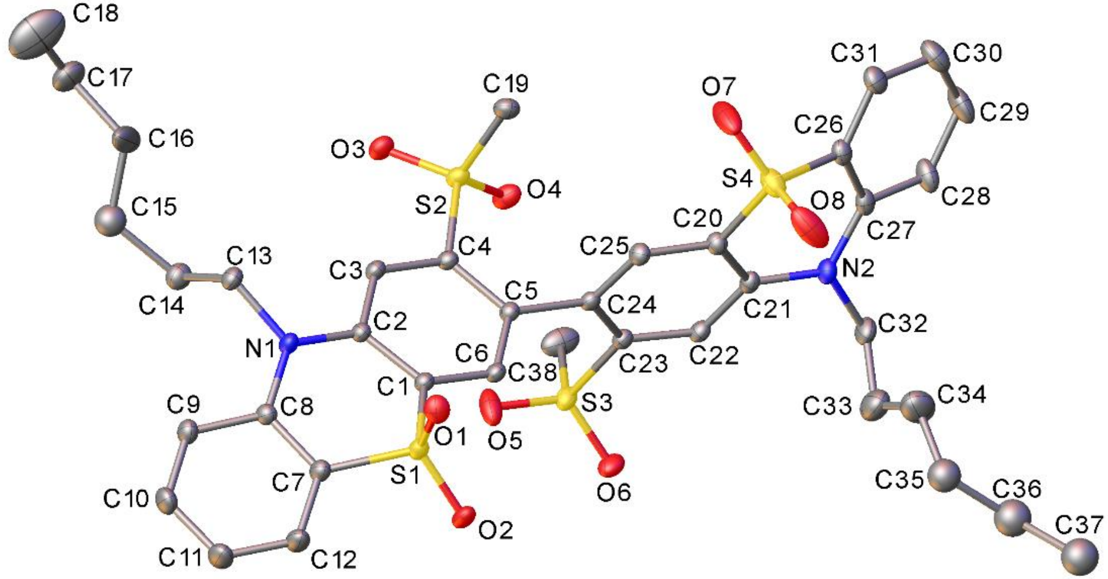

Figure S4.4 X-ray molecular structure of **4**, showing major conformations of the disordered n-hexyl chains. H atoms are omitted for clarity.

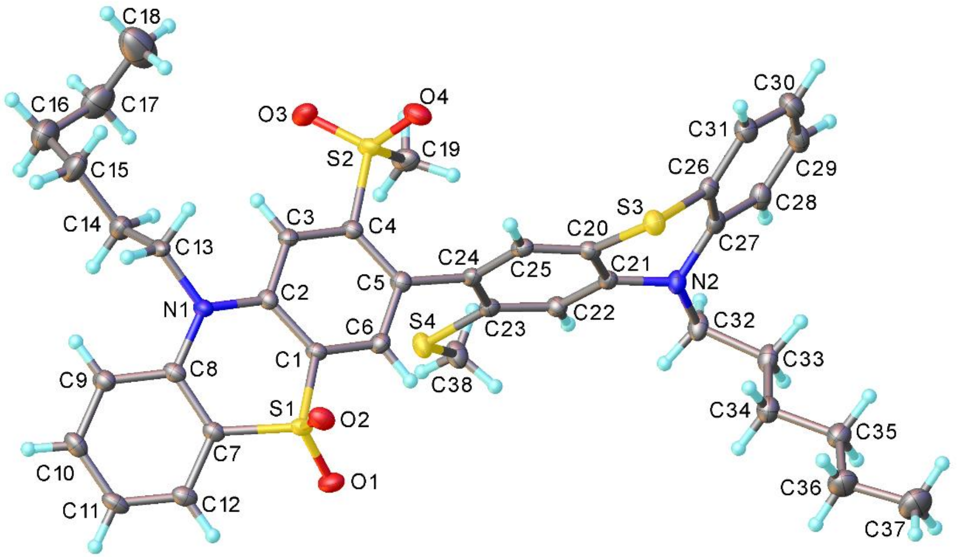

Figure S4.5 X-ray molecular structure of **5**.

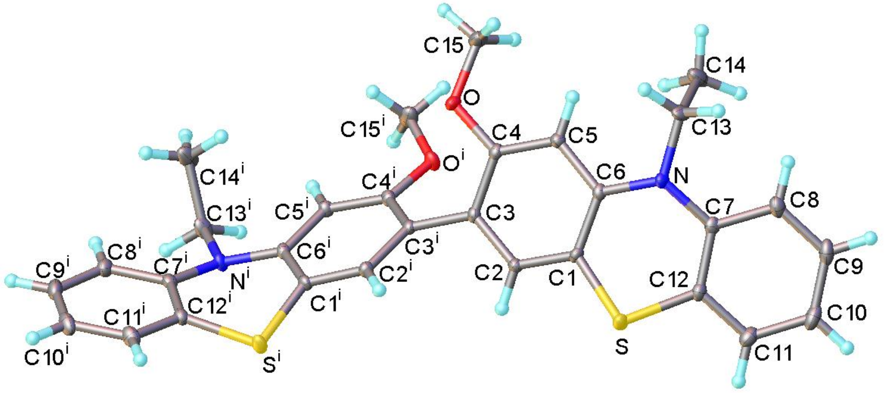

Figure S4.6 X-ray molecular structure of **9**. Primed atoms are generated by the twofold axis.

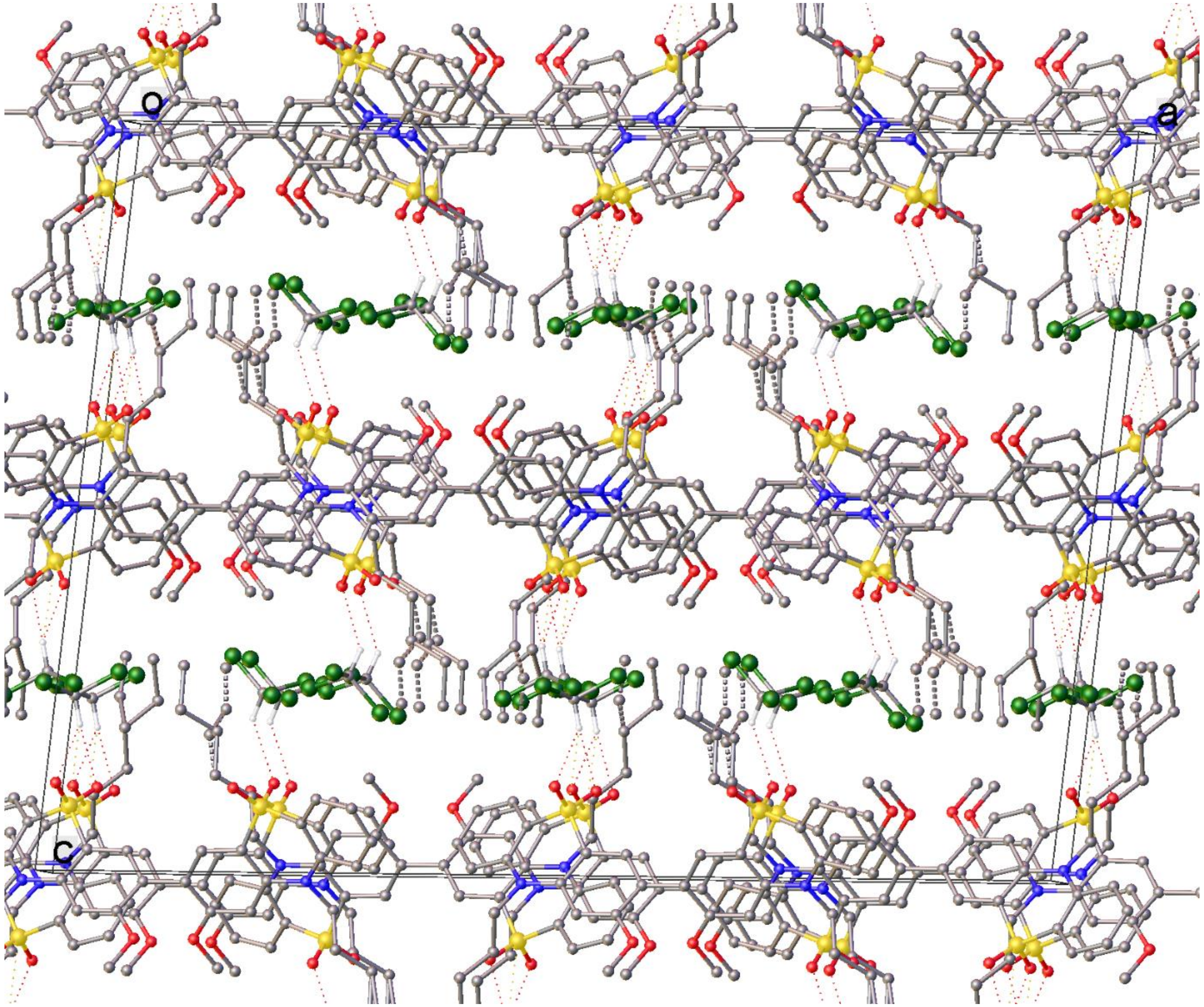

Figure S4.7 Crystal packing of **3**·$CDCl_3$ viewed down the *y* axis (H atoms are omitted). Note the segregated layers of ordered PTZ dimers alternating with layers of disordered *n*-hexyl chains (minor positions dashed) and d-chloroform molecules having non-stoichiometric occupancies. Red dots indicate C-D…O hydrogen bonds.

5. Electrochemistry

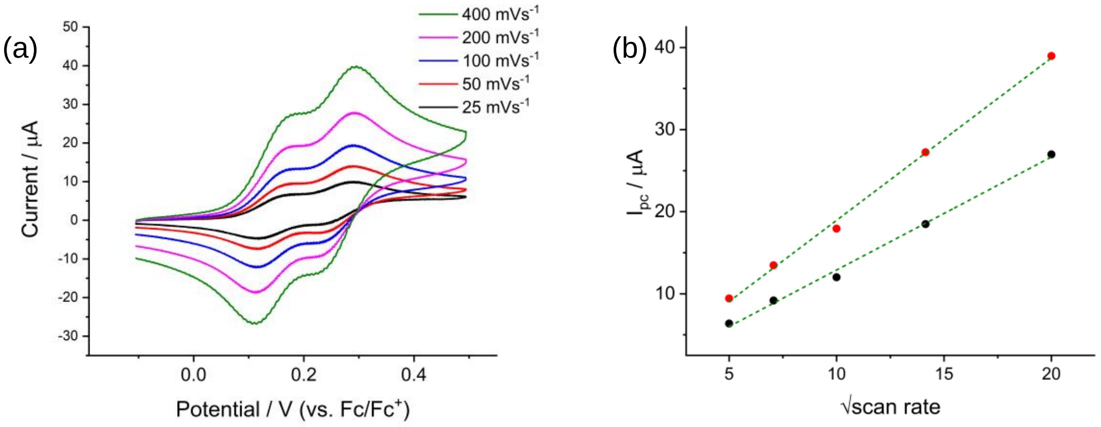

Figure S5.1 (a) Cyclic voltammetry of **1** at varying scan rates. (b) Linear dependence of peak current with the square root of the scan rate for both the first (black) and second (red) oxidation of 1 ($R^2 > 0.99$ in both cases).

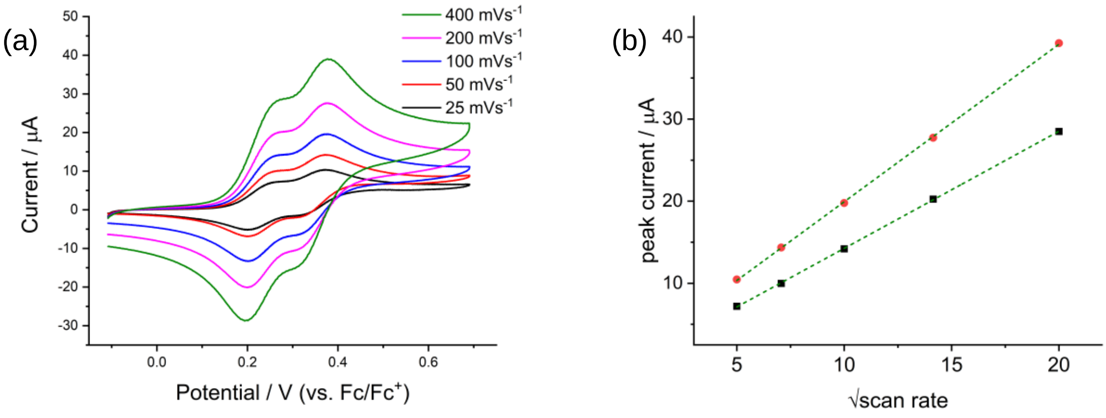

Figure S5.2 (a) Cyclic voltammetry of **2** at varying scan rates. (b) Linear dependence of peak current with the square root of the scan rate for both the first (black) and second (red) oxidation of 1 ($R^2 > 0.99$ in both cases).

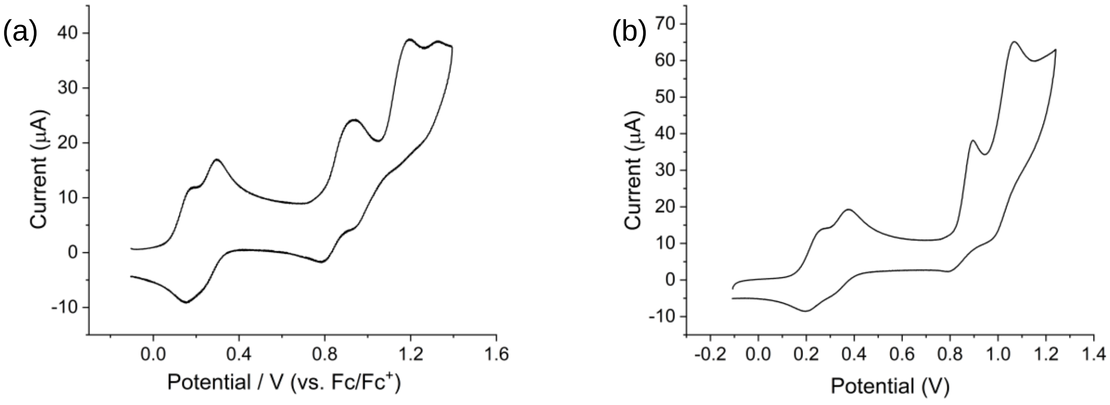

Figure S5.3 Wider cyclic voltammetry scans for (a) **1** and (b) **2** showing irreversible oxidation processes at higher applied potential.

6. Computational Results

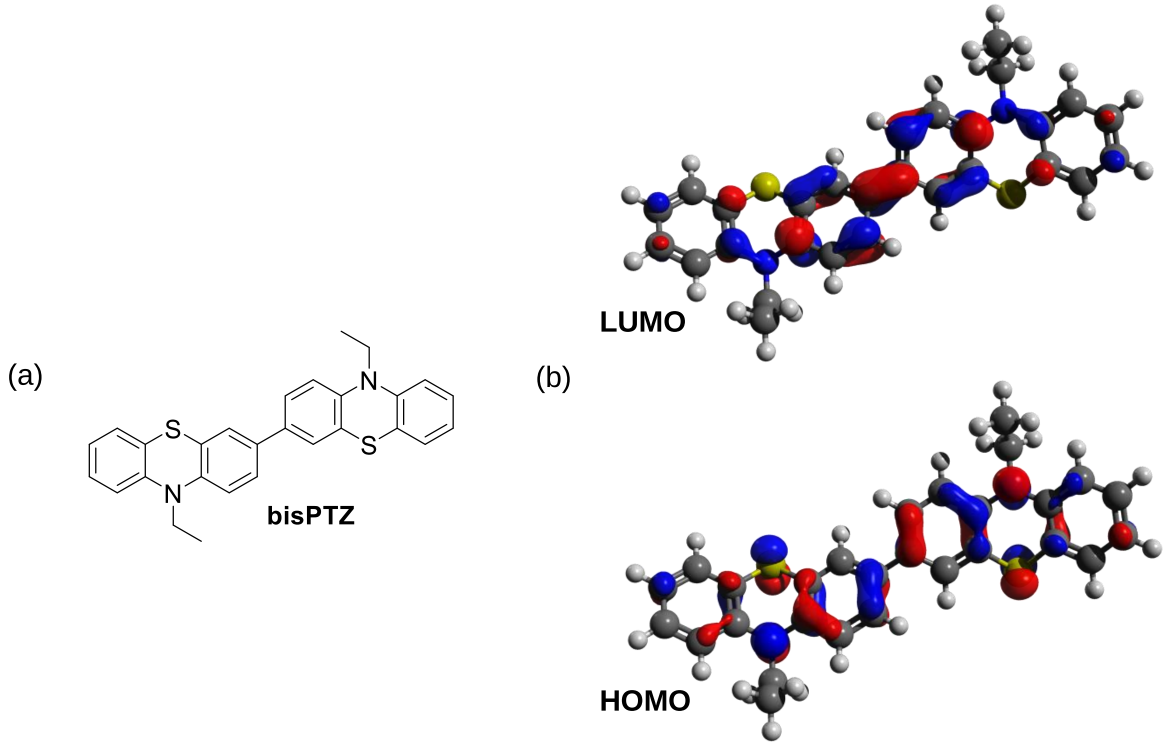

Figure S6.1 (a) Molecular structure of **bisPTZ** and (b) its DFT optimized structure and HOMO and LUMO distributions.

Table S6.1 DFT calculated structural properties of the radical cations of **1-5** and model compound **bisPTZ**.

	$\angle_{C2C3C3'C2'}{}^{RC}$ (°)	τ^{RC} (°)	θ^{RC} (°)
**1**	142.13	39.30	16.11, 16.09
**2**	63.63	60.70	17.73, 17.70
**3**	150.11	33.98	22.48, 22.53
**4**	123.54	60.28	24.74, 24.85
**5**	81.51	78.88	25.94 (SO2), 10.61 (S)
**bisPTZ**	154.52	25.58	16.63, 16.69

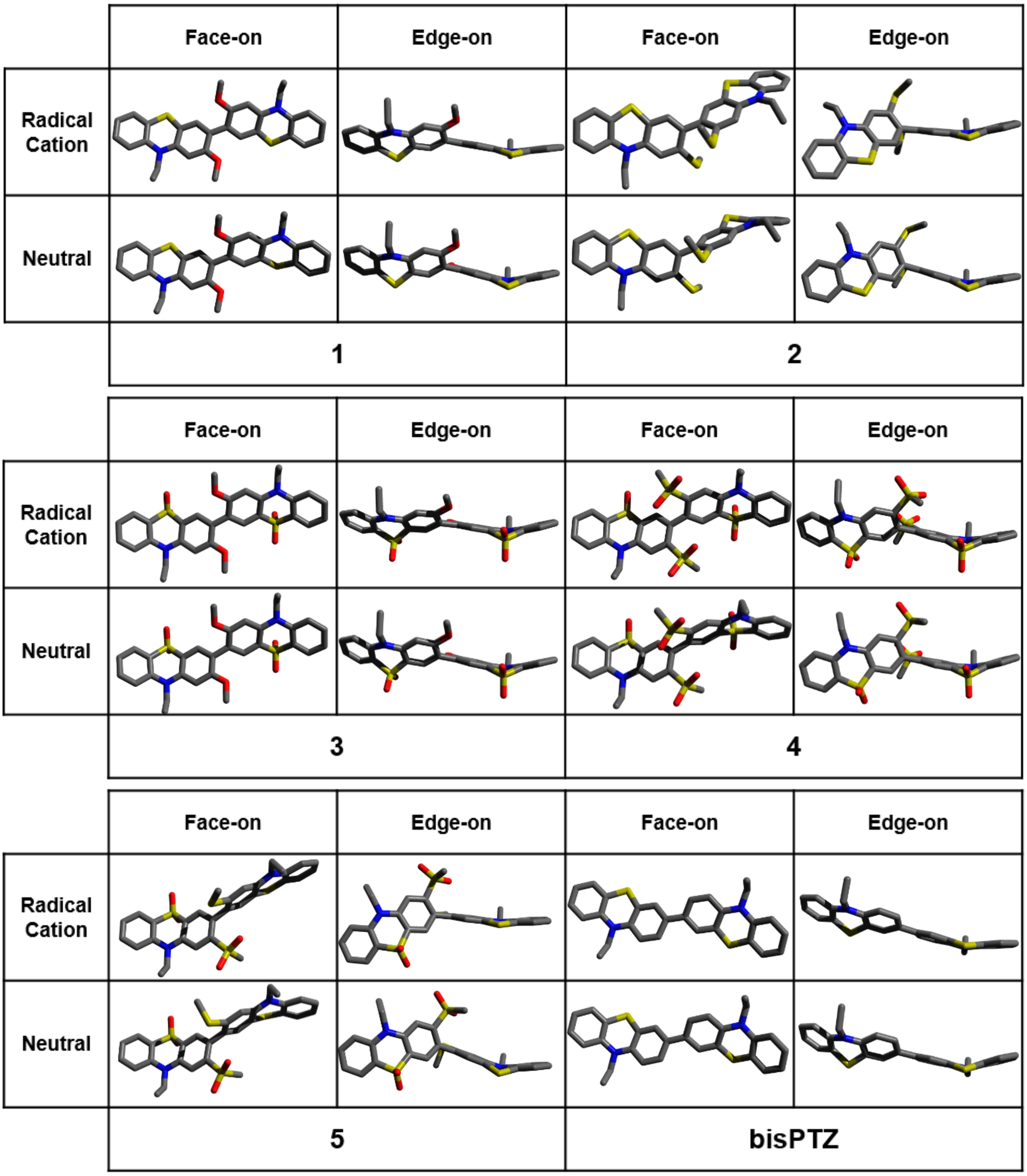

Figure S6.2 DFT optimized molecular structure of the ground state and radical cations of **1**-**5** and model compound **bisPTZ**. Face-on and edge-on perspectives are provided for each molecule with the edge-on view providing the best demonstrations of the greater planarization of **PTZ** compared to **PTZ(SO_2)** heterocycles upon electrochemical oxidation. This is most clearly shown for **5**. Ethyl rather than hexyl chains were appended to the nitrogen atoms of the phenothiazines in order to reduce the computational cost.

Table S6.2 Transitions to the first 10 singlet and first 10 triplet states calculated for the optimized ground state geometry of **1**/**9** at TD-B3LYP/6-31G**.

No.	Transition	Energy (cm^{-1})	Wavelength (nm)	*f*	Orbital contribution
1	$S_0 \rightarrow T_1$	22534	444	0	HOMO→LUMO (83%)
2	$S_0 \rightarrow T_2$	24336	411	0	H−1→LUMO (57%), HOMO→L+1 (29%)
3	$S_0 \rightarrow S_1$	26901	372	0.41	HOMO→LUMO (89%)
4	$S_0 \rightarrow T_3$	27521	363	0	H−1→L+1 (31%), HOMO→L+2 (32%)
5	$S_0 \rightarrow T_4$	27871	359	0	H−1→L+2 (32%), HOMO→L+3 (47%)
6	$S_0 \rightarrow T_5$	28048	357	0	H−1→L+3 (25%), HOMO→L+2 (17%), HOMO→L+4 (43%)
7	$S_0 \rightarrow S_2$	28355	353	0.0005	H−1→LUMO (71%), HOMO→L+1 (22%)
8	$S_0 \rightarrow T_6$	28529	351	0	H−1→LUMO (16%), H−1→L+4 (25%), HOMO→L+1 (16%), HOMO→L+5 (31%)
9	$S_0 \rightarrow T_7$	29746	336	0	H−1→L+5 (19%), HOMO→L+6 (31%)
10	$S_0 \rightarrow T_8$	30208	331	0	H−1→L+6 (20%), HOMO→L+5 (27%)
11	$S_0 \rightarrow S_3$	30884	324	0.001	H−1→LUMO (24%), HOMO→L+2 (64%)
12	$S_0 \rightarrow S_4$	31732	315	0.36	H−1→L+1 (16%), HOMO→L+2 (58%), HOMO→L+4 (13%)
13	$S_0 \rightarrow T_9$	31898	314	0	H−1→LUMO (13%), HOMO→L+1 (23%), HOMO→L+7 (13%)
14	$S_0 \rightarrow S_5$	32257	310	0.016	HOMO→L+3 (80%)
15	$S_0 \rightarrow S_6$	32577	307	0.055	H−1→L+1 (15%), HOMO→L+2 (23%), HOMO→L+4 (40%)
16	$S_0 \rightarrow T_{10}$	32789	305	0	H−2→LUMO (41%), H−2→L+4 (11%)
17	$S_0 \rightarrow S_7$	33651	297	0.047	H−1→L+2 (50%), HOMO→L+4 (31%)
18	$S_0 \rightarrow S_8$	33862	295	0.004	H−3→L+3 (10%), HOMO→L+5 (71%)
19	$S_0 \rightarrow S_9$	35266	284	0.006	H−1→L+3 (72%), HOMO→L+2 (15%)
20	$S_0 \rightarrow S_{10}$	35329	283	0.006	H−1→L+2 (75%), HOMO→L+3 (11%)

Table S6.3 Transitions to the first 10 singlet and first 10 triplet states calculated for the optimized ground state geometry of **2** at TD-B3LYP/6-31G**.

**No.**	**Transition**	**Energy (cm^{-1})**	**Wavelength (nm)**	***f***	**Orbital contribution**
1	$S_0 \rightarrow T_1$	23391	428	0	HOMO→LUMO (65%), H−1→L+1 (24%)
2	$S_0 \rightarrow T_2$	23750	421	0	H−1→LUMO (41%), HOMO→L+1 (47%)
3	$S_0 \rightarrow T_3$	26936	371	0	H−1→L+3 (23%), HOMO→L+1 (17%), HOMO→L+2 (48%)
4	$S_0 \rightarrow T_4$	27355	366	0	H−1→L+1 (14%), H−1→L+2 (33%), HOMO→L+3 (44%)
5	$S_0 \rightarrow S_1$	27575	363	0.080	H−1→L+1 (17%), HOMO→LUMO (77%)
6	$S_0 \rightarrow S_2$	28032	357	0.009	H−1→LUMO (36%), HOMO→L+1 (55%)
7	$S_0 \rightarrow T_5$	28576	350	0	H−1→L+1 (19%), H−1→L+5 (15%), HOMO→L+4 (36%)
8	$S_0 \rightarrow T_6$	28861	347	0	H−1→L+4 (31%), HOMO→L+5 (40%)
9	$S_0 \rightarrow T_7$	29690	337	0	H−1→L+5 (23%), HOMO→L+4 (15%), HOMO→L+6 (25%)
10	$S_0 \rightarrow S_3$	30134	332	0.012	H−1→LUMO (52%), HOMO→L+1 (40%)
11	$S_0 \rightarrow T_8$	30204	331	0	H−1→LUMO (15%), H−1→L+6 (24%), HOMO→L+5 (12%), HOMO→L+7 (14%)
12	$S_0 \rightarrow S_4$	30662	326	0.12	H−1→L+1 (76%), HOMO→LUMO (19%), HOMO→L+2 (58%), HOMO→L+4 (13%)
13	$S_0 \rightarrow T_9$	31062	322	0	H−1→LUMO (27%), HOMO→L+1 (13%), HOMO→L+2 (11%), HOMO→L+7 (13%)
14	$S_0 \rightarrow T_{10}$	31323	319	0	H−1→L+1 (28%), HOMO→LUMO (15%), HOMO→L+3 (14%)
15	$S_0 \rightarrow S_5$	32173	311	0.044	HOMO→L+2 (78%)
16	$S_0 \rightarrow S_6$	32493	308	0.13	H−1→L+2 (22%), HOMO→L+3 (52%), HOMO→L+4 (12%)
17	$S_0 \rightarrow S_7$	33242	301	0.16	H−1→L+2 (16%), H−1→L+5 (12%), HOMO→L+4 (57%)
18	$S_0 \rightarrow S_8$	33527	298	0.009	H−1→L+4 (23%), HOMO→L+5 (57%)
19	$S_0 \rightarrow S_9$	33817	296	0.005	H−1→L+2 (46%), H−1→L+5 (10%), HOMO→L+4 (35%)
20	$S_0 \rightarrow S_{10}$	34255	292	0.001	H−2→LUMO (25%), H−1→L+3 (49%)

Table S6.4 Transitions to the first 10 singlet and first 10 triplet states calculated for the optimized ground state geometry of **3** at TD-B3LYP/6-31G**.

**No.**	**Transition**	**Energy (cm^{-1})**	**Wavelength (nm)**	***ƒ***	**Orbital contribution**
1	$S_0 \rightarrow T_1$	25283	396	0	HOMO→LUMO (79%)
2	$S_0 \rightarrow T_2$	28472	352	0	H−1→L+2 (11%), HOMO→L+1 (70%)
3	$S_0 \rightarrow T_3$	28649	349	0	H−1→L+1 (18%), HOMO→L+2 (66%)
4	$S_0 \rightarrow T_4$	28946	346	0	H−1→LUMO (33%), HOMO→L+3 (36%),
5	$S_0 \rightarrow T_5$	30904	324	0	H−2→LUMO (16%), H−1→L+3 (22%), HOMO→L+6 (17%)
6	$S_0 \rightarrow S_1$	30961	323	1.18	HOMO→LUMO (93%)
7	$S_0 \rightarrow T_6$	31886	314	0	H−2→L+1 (11%), H−1→L+2 (12%), HOMO→L+4 (40%)
8	$S_0 \rightarrow T_7$	32054	312	0	H−2→L+2 (15%), H−1→L+2 (11%), HOMO→L+5 (39%)
9	$S_0 \rightarrow S_2$	32146	311	0.015	HOMO→L+1 (89%)
10	$S_0 \rightarrow T_8$	32537	307	0	H−1→L+6 (10%), HOMO→L+7 (18%)
11	$S_0 \rightarrow S_3$	32782	305	0.37	HOMO→L+2 (87%)
12	$S_0 \rightarrow T_9$	33940	295	0	H−3→LUMO (15%)
13	$S_0 \rightarrow T_{10}$	33969	294	0	H−3→LUMO (11%), H−3→L+1 (10%)
14	$S_0 \rightarrow S_4$	35586	281	0.0009	H−1→LUMO (87%)
15	$S_0 \rightarrow S_5$	36116	277	0.0009	H−3→LUMO (12%), HOMO→L+3 (61%), HOMO→L+4 (17%)
16	$S_0 \rightarrow S_6$	36618	273	0.024	H−2→LUMO (20%), H−1→L+1 (10%), HOMO→L+5 (55%)
17	$S_0 \rightarrow S_7$	36924	271	0.003	HOMO→L+3 (26%), HOMO→L+4 (47%)
18	$S_0 \rightarrow S_8$	36939	271	0.039	H−1→L+1 (70%)
19	$S_0 \rightarrow S_9$	37429	267	0.001	H−1→L+2 (78%)
20	$S_0 \rightarrow S_{10}$	39198	255	0.061	H−2→LUMO (46%), HOMO→L+5 (25%), HOMO→L+6 (10%)

Table S6.4 Transitions to the first 10 singlet and first 10 triplet states calculated for the optimized ground state geometry of **4** at TD-B3LYP/6-31G**.

**No.**	**Transition**	**Energy (cm^{-1})**	**Wavelength (nm)**	***f***	**Orbital contribution**
1	$S_0 \rightarrow T_1$	25648	390	0	HOMO→LUMO (54%), H−1→L+1 (27%)
2	$S_0 \rightarrow T_2$	25666	390	0	H−1→LUMO (54%), HOMO→L+1 (28%)
3	$S_0 \rightarrow T_3$	28096	356	0	H−1→L+3 (26%), HOMO→L+2 (43%)
4	$S_0 \rightarrow T_4$	28178	355	0	H−1→L+2 (42%), HOMO→L+3 (26%)
5	$S_0 \rightarrow S_1$	30468	328	0.11	HOMO→LUMO (84%)
6	$S_0 \rightarrow S_2$	30588	327	0.036	H−1→LUMO (84%)
7	$S_0 \rightarrow T_5$	31277	320	0	H−1→L+1 (30%), HOMO→LUMO (16%), HOMO→L+4 (15%)
8	$S_0 \rightarrow T_6$	31351	319	0	H−1→LUMO (18%), H−1→L+4 (16%), HOMO→L+1 (34%)
9	$S_0 \rightarrow T_7$	31821	314	0	H−1→L+7 (15%), HOMO→L+6 (12%)
10	$S_0 \rightarrow T_8$	31890	314	0	H−1→L+6 (13%), HOMO→L+7 (17%)
11	$S_0 \rightarrow T_9$	32123	311	0	H−1→L+4 (17%), HOMO→L+1 (19%), HOMO→L+5 (18%)
12	$S_0 \rightarrow T_{10}$	32264	310	0	H−1→L+1 (25%), H−1→L+5 (13%), HOMO→LUMO (12%), HOMO→L+4 (15%)
13	$S_0 \rightarrow S_3$	32317	309	0.083	H−1→L+1 (71%), HOMO→L+1 (23%)
14	$S_0 \rightarrow S_4$	32357	309	0.022	H−1→L+1 (23%), HOMO→L+1 (69%)
15	$S_0 \rightarrow S_5$	34490	290	0.84	H−1→L+3 (19%), HOMO→L+2 (63%)
16	$S_0 \rightarrow S_6$	35339	283	0.014	H−1→L+2 (73%)
17	$S_0 \rightarrow S_7$	35819	279	0.021	H−1→L+2 (64%), HOMO→L+2 (20%)
18	$S_0 \rightarrow S_8$	35861	279	0.009	HOMO→L+3 (75%)
19	$S_0 \rightarrow S_9$	37895	264	0.27	H−1→L+4 (10%), H−1→L+5 (31%), HOMO→L+4 (41%)
20	$S_0 \rightarrow S_{10}$	38098	263	0.002	H−1→L+4 (39%), HOMO→L+5 (31%)

Table S6.5 Transitions to the first 10 singlet and first 10 triplet states calculated for the optimized ground state geometry of **5** at TD-B3LYP/6-31G**.

No.	Transition	Energy (cm^{-1})	Wavelength (nm)	*f*	Orbital contribution
1	$S_0 \rightarrow T_1$	23428	427	0	HOMO→LUMO (29%), HOMO→L+2 (55%)
2	$S_0 \rightarrow T_2$	24207	413	0	HOMO→LUMO (61%), HOMO→L+2 (16%)
3	$S_0 \rightarrow S_1$	24485	408	0.025	HOMO→LUMO (98%)
4	$S_0 \rightarrow T_3$	25781	388	0	H−2→LUMO (23%), H−1→LUMO (63%)
5	$S_0 \rightarrow T_4$	27350	366	0	HOMO→L+3 (10%), HOMO→L+4 (68%)
6	$S_0 \rightarrow S_2$	27764	360	0.043	HOMO→L+1 (33%), HOMO→L+2 (57%)
7	$S_0 \rightarrow T_5$	28035	357	0	H−2→L+1 (19%), H−1→L+1 (32%), HOMO→L+1 (24%)
8	$S_0 \rightarrow T_6$	28935	346	0	H−1→L+1 (14%), HOMO→L+1 (33%), HOMO→L+5 (24%)
9	$S_0 \rightarrow S_3$	29107	344	0.004	HOMO→L+1 (65%), HOMO→L+2 (24%)
10	$S_0 \rightarrow T_7$	29373	340	0	HOMO→L+1 (22%), HOMO→L+5 (57%)
11	$S_0 \rightarrow S_4$	29955	334	0.02	H−1→LUMO (86%)
12	$S_0 \rightarrow T_8$	30112	332	0	H−2→LUMO (54%), H−1→LUMO (21%)
13	$S_0 \rightarrow T_9$	30577	327	0	HOMO→L+7 (44%)
14	$S_0 \rightarrow T_{10}$	31568	317	0	H−2→L+3 (18%), H−1→L+2 (12%), H−1→L+3 (21%)
15	$S_0 \rightarrow S_5$	31698	316	0.15	H−2→LUMO (70%)
16	$S_0 \rightarrow S_6$	31886	314	0.063	H−2→LUMO (12%), HOMO→L+2 (29%), HOMO→L+3 (39%)
17	$S_0 \rightarrow S_7$	32987	303	0.014	HOMO→L+2 (48%), HOMO→L+3 (34%)
18	$S_0 \rightarrow S_8$	33631	297	0.18	H−1→L+1 (48%), H−1→L+2 (24%)
19	$S_0 \rightarrow S_9$	33840	296	0.09	HOMO→L+5 (74%),
20	$S_0 \rightarrow S_{10}$	34085	293	0.039	H−3→LUMO (92%)

Table S6.6 Transitions to the first 10 singlet and first 10 triplet states calculated for the optimized ground state geometry of **bisPTZ** at TD-B3LYP/6-31G**.

No.	Transition	Energy (cm^{-1})	Wavelength (nm)	*f*	Orbital contribution
1	$S_0 \rightarrow T_1$	22139	452	0	HOMO→LUMO (85%)
2	$S_0 \rightarrow T_2$	24388	410	0	H−1→LUMO (57%), HOMO→L+1 (28%)
3	$S_0 \rightarrow S_1$	26730	374	0.50	HOMO→LUMO (89%)
4	$S_0 \rightarrow T_3$	27199	368	0	H−1→L+3 (17%), HOMO→L+1 (34%), HOMO→L+2 (29%)
5	$S_0 \rightarrow T_4$	27479	364	0	H−1→L+1 (29%), HOMO→L+3 (39%), HOMO→L+4 (19%)
6	$S_0 \rightarrow T_5$	27899	358	0	H−1→L+2 (33%), HOMO→L+3 (20%), HOMO→L+4 (19%)
7	$S_0 \rightarrow S_2$	28151	355	0.0002	H−1→LUMO (75%), HOMO→L+1 (16%)
8	$S_0 \rightarrow T_6$	28546	350	0	H−1→LUMO (10%), H−1→L+4 (26%), HOMO→L+2 (10%), HOMO→L+2 (25%), HOMO→L+5 (15%)
9	$S_0 \rightarrow T_7$	29538	339	0	H−1→L+5 (29%), HOMO→L+4 (21%), HOMO→L+6 (27%)
10	$S_0 \rightarrow T_8$	30107	332	0	H−1→LUMO (13%), H−1→L+6 (23%), HOMO→L+5 (38%)
11	$S_0 \rightarrow S_3$	30440	329	0.0026	H−1→LUMO (17%), HOMO→L+1 (74%)
12	$S_0 \rightarrow S_4$	31786	315	0.34	H−1→L+1 (22%), HOMO→L+3 (17%), HOMO→L+4 (45%)
13	$S_0 \rightarrow T_9$	31942	313	0	H−1→L+1 (34%), HOMO→L+3 (12%)
14	$S_0 \rightarrow S_5$	32433	308	0.022	HOMO→L+2 (82%)
15	$S_0 \rightarrow T_{10}$	32517	308	0	H−3→L+3 (10%), H−2→L+1 (13%), H−1→L+3 (17%)
16	$S_0 \rightarrow S_6$	32745	305	0.107	HOMO→L+3 (63%), HOMO→L+4 (19%)
17	$S_0 \rightarrow S_7$	33404	299	0.081	H−1→L+1 (69%), HOMO→L+4 (16%)
18	$S_0 \rightarrow S_8$	33939	295	0.0002	HOMO→L+5 (73%)
19	$S_0 \rightarrow S_9$	34937	286	0.002	H−1→L+3 (57%), H−1→L+4 (30%)
20	$S_0 \rightarrow S_{10}$	35318	283	0.18	H−1→L+2 (60%), H−1→L+5 (16%)

7. Photophysics

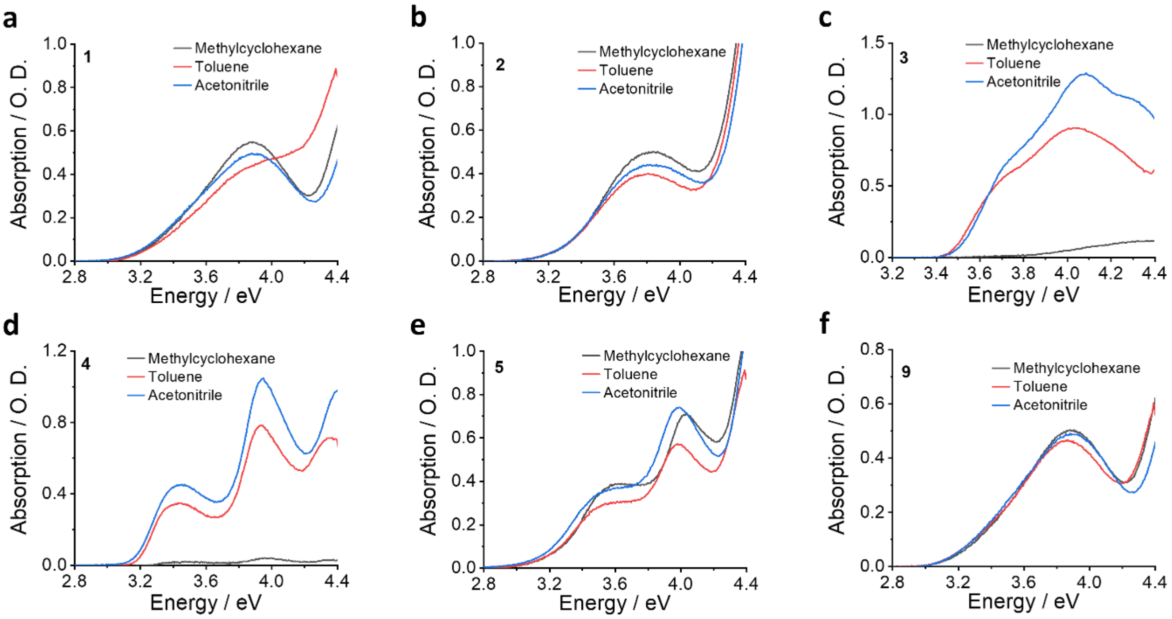

Figure S7.1 Absorption spectra of compound a) **1**, b) **2**, c) **3**, d) **4**, e) **5** and f) **9** measured at 40 µM concentration.

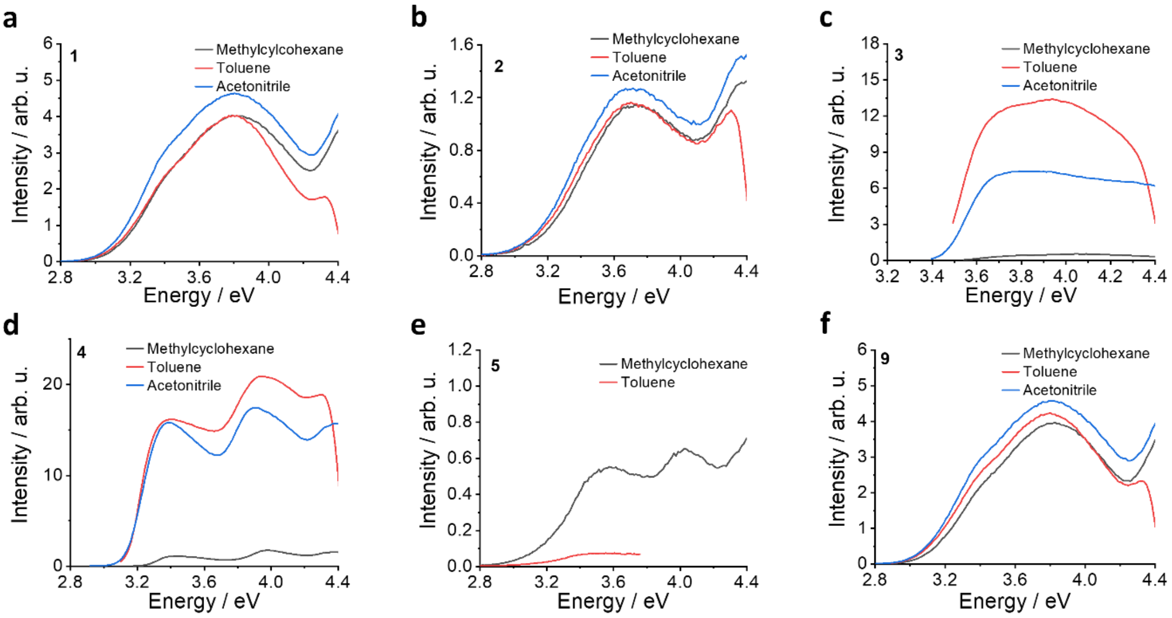

Figure S7.2 Excitation spectra of compound a) **1**, b) **2**, c) **3**, d) **4**, e) **5** and f) **9** measured at 40 µM concentration to the peak of the emission band.

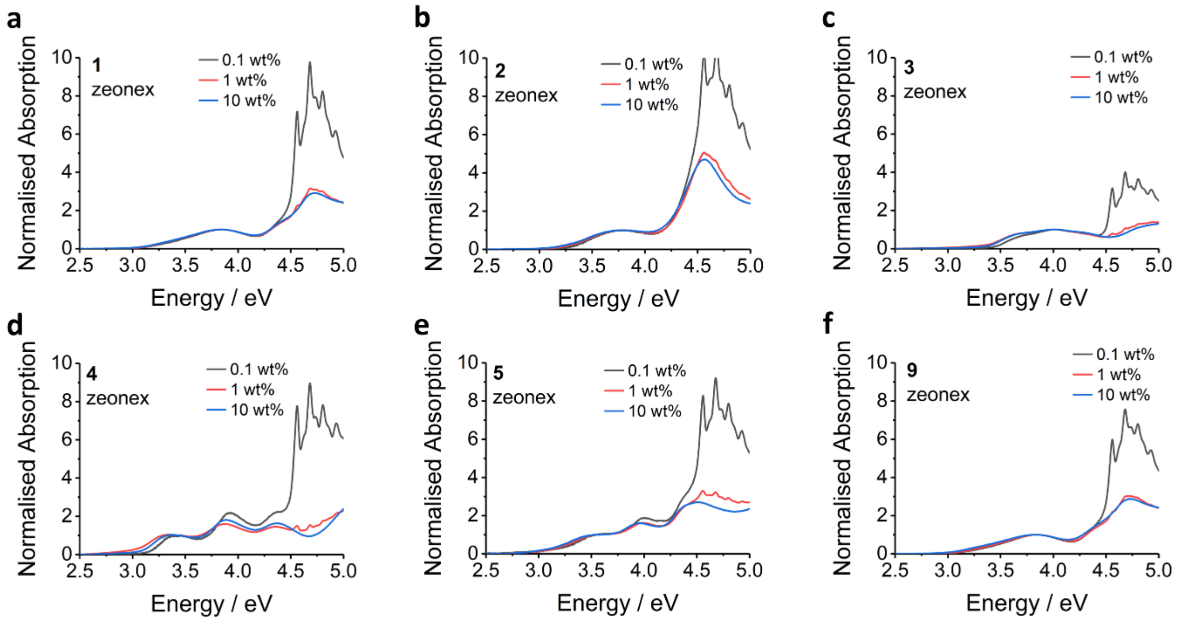

Figure S7.3 Absorption spectra of compound a) **1**, b) **2**, c) **3**, d) **4**, e) **5** and f) **9** measured in zeonex films at different weight percentages.

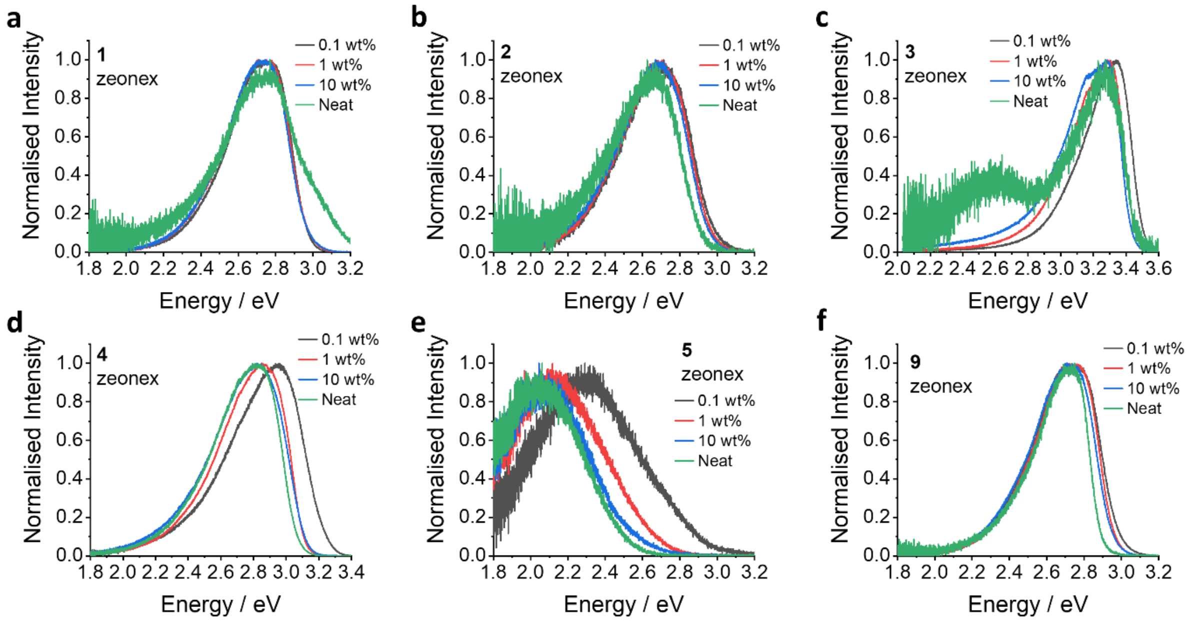

Figure S7.4 Photoluminescence spectra of compound a) **1**, b) **2**, c) **3**, d) **4**, e) **5** and f) **9** measured in zeonex films at different weight percentages and in neat film.

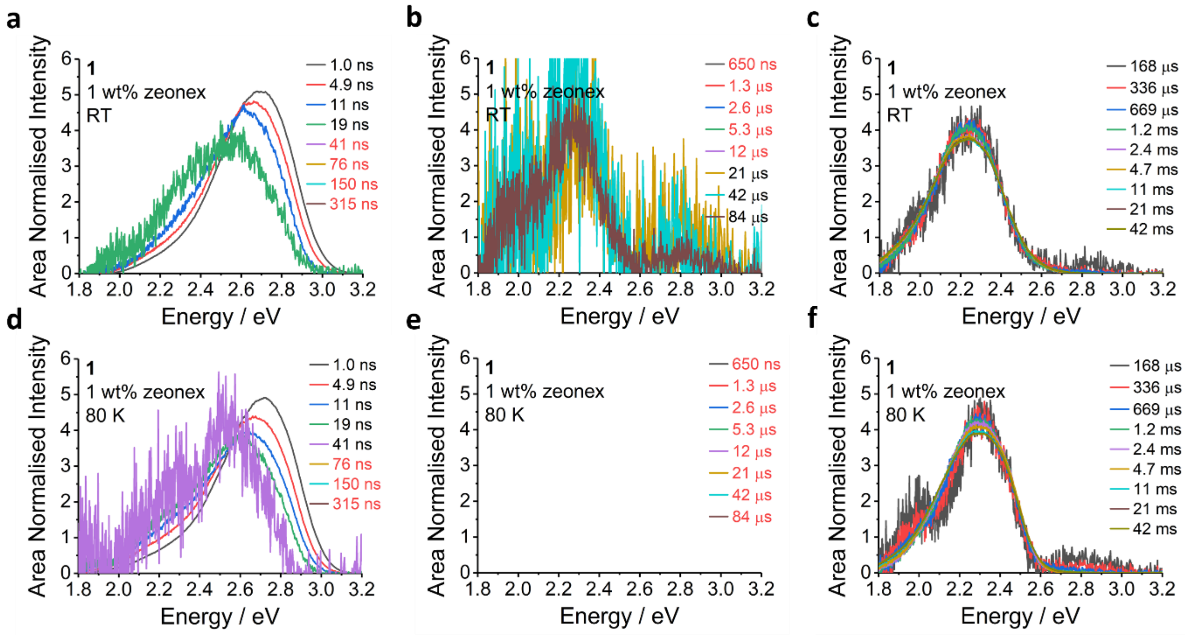

Figure S7.5 Time resolved photoluminescence spectra of **1** at 1 wt% in zeonex. Measured at (a-c) room temperature and (d-f) 80 K.

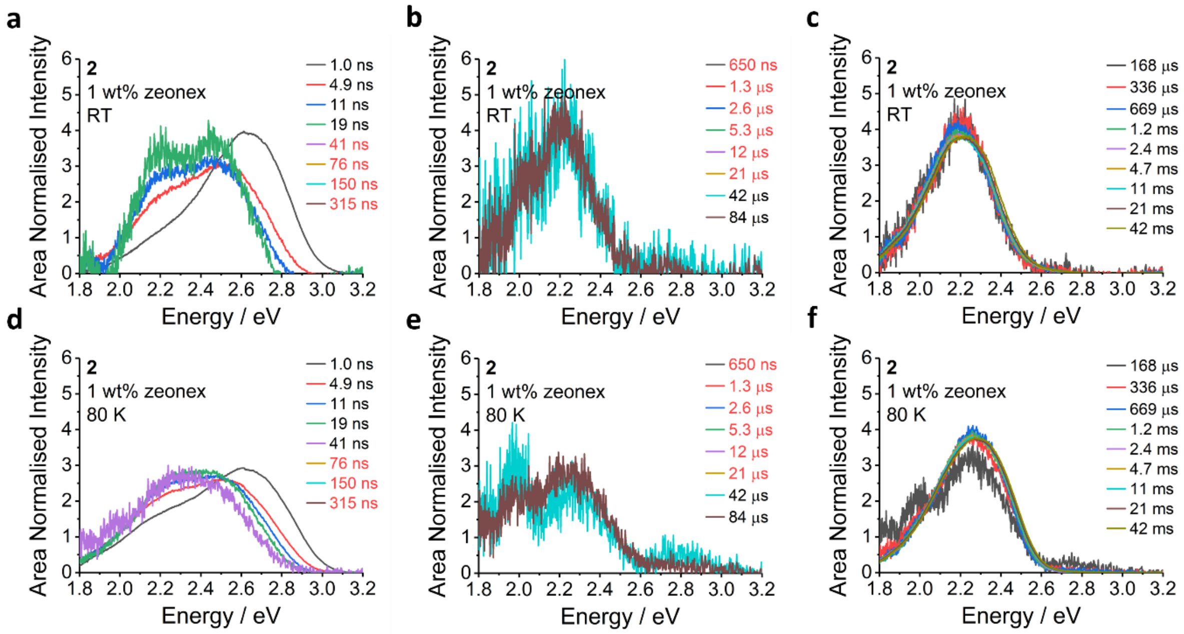

Figure S7.6 Time resolved photoluminescence spectra of **2** at 1 wt% in zeonex. Measured at (a-c) room temperature and (d-f) 80 K.

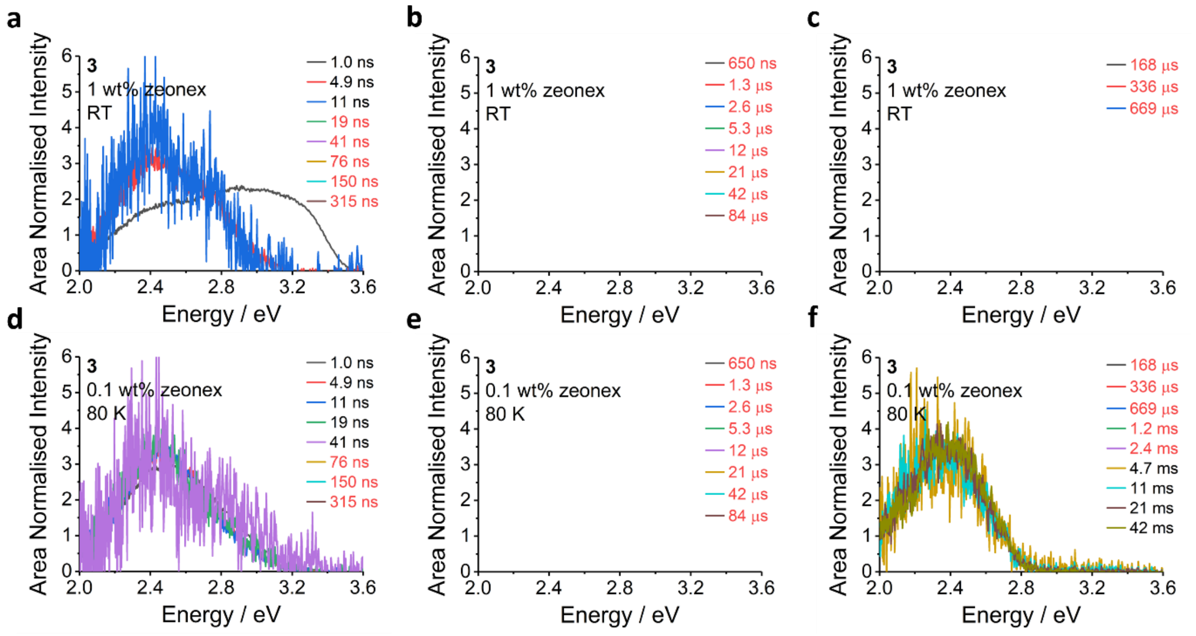

Figure S7.7 Time resolved photoluminescence spectra of **3** at (a-c) 1 wt% in zeonex at room temperature and at (d-f) 0.1 wt% in zeonex at 80 K.

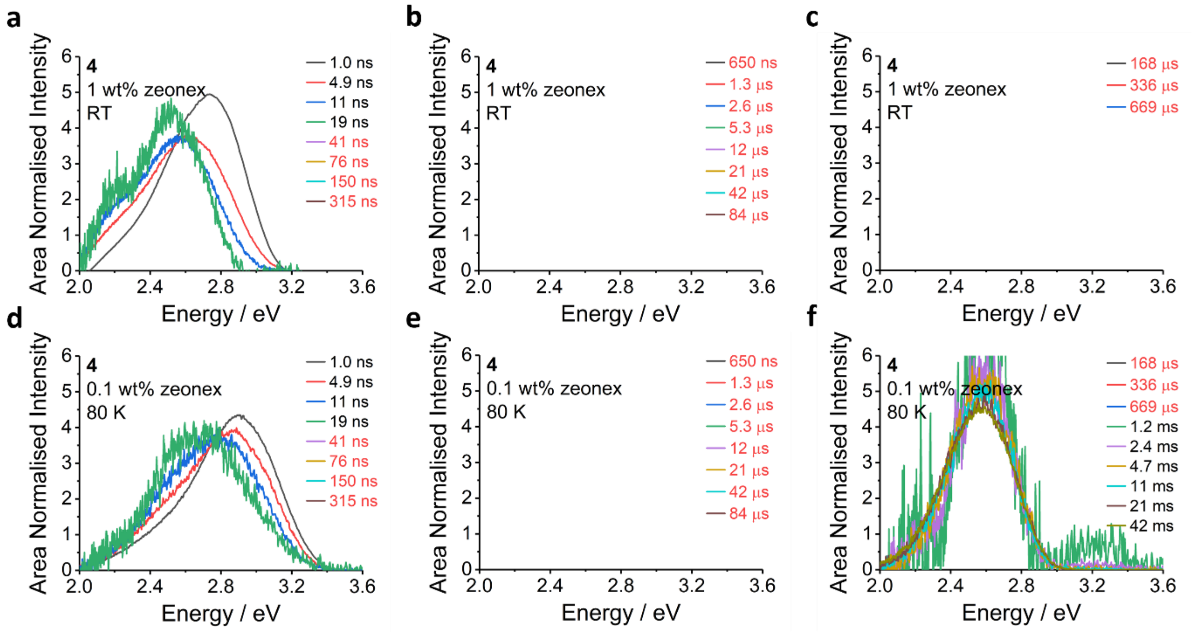

Figure S7.8 Time resolved photoluminescence spectra of **4** at (a-c) 1 wt% in zeonex at room temperature and at (d-f) 0.1 wt% in zeonex at 80 K.

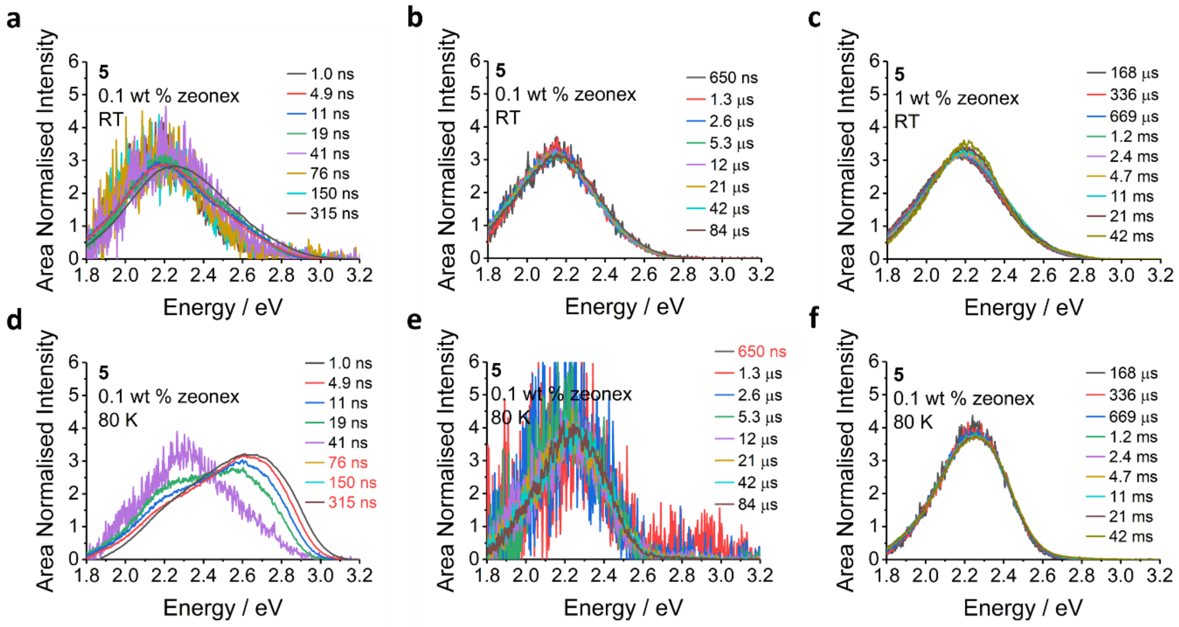

Figure S7.9 Time resolved photoluminescence spectra of **5** at 0.1 wt% in zeonex. Measured at (a-c) room temperature and (d-f) 80 K.

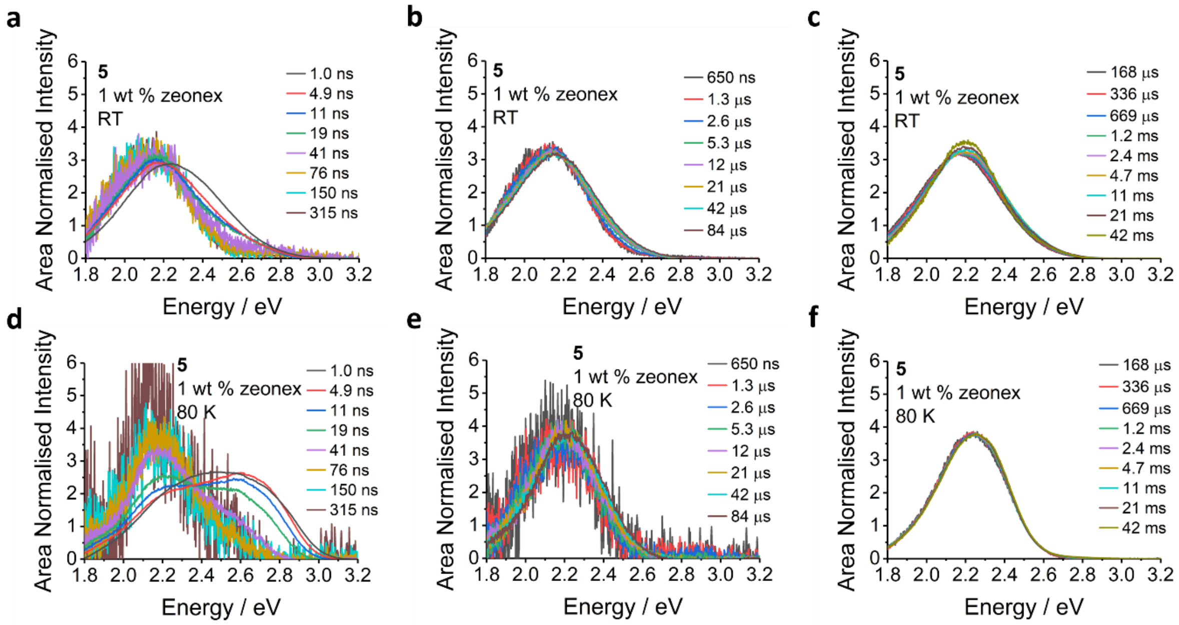

Figure S7.10 Time resolved photoluminescence spectra of **5** at 1 wt% in zeonex. Measured at (a-c) room temperature and (d-f) 80 K.

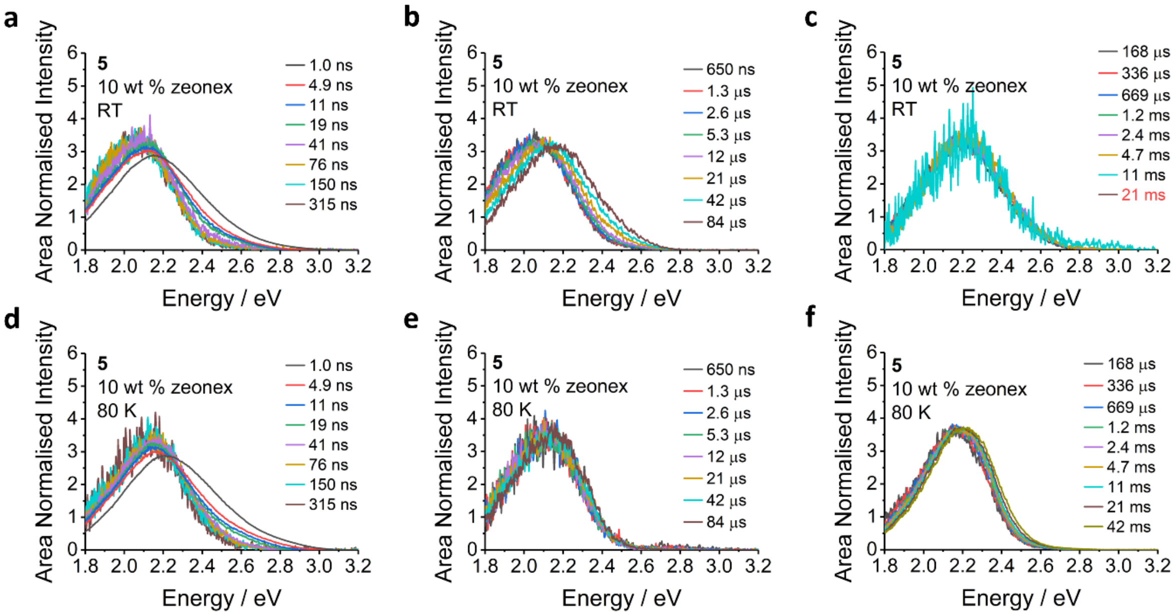

Figure S7.11 Time resolved photoluminescence spectra of **5** at 10 wt% in zeonex. Measured at (a-c) room temperature and (d-f) 80 K.

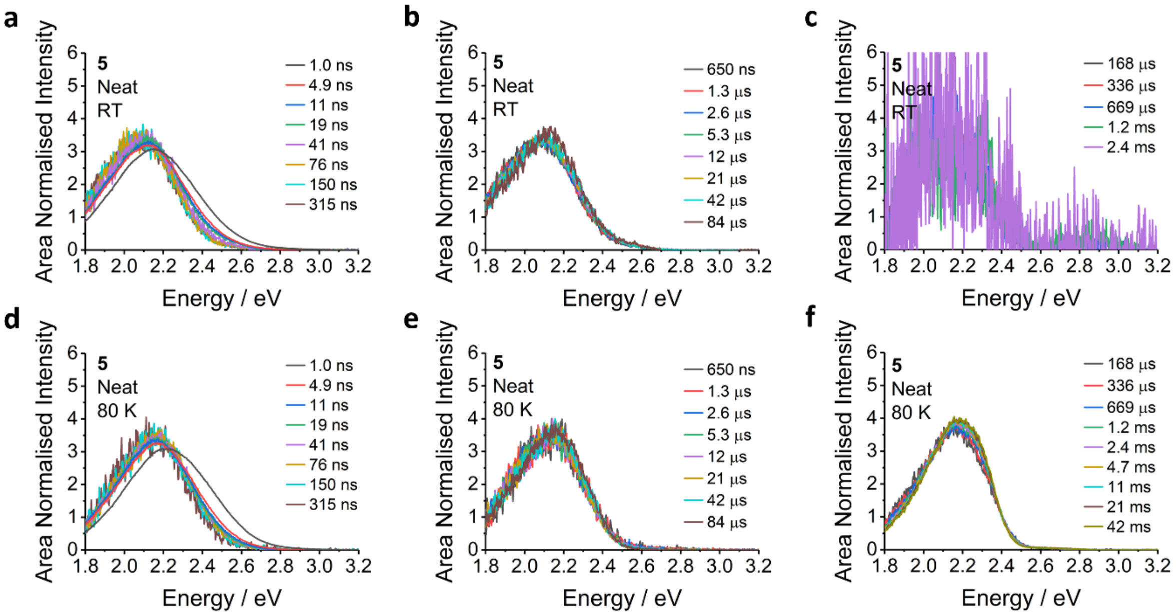

Figure S7.12 Time resolved photoluminescence spectra of **5** in neat film. Measured at (a-c) room temperature and (d-f) 80 K.

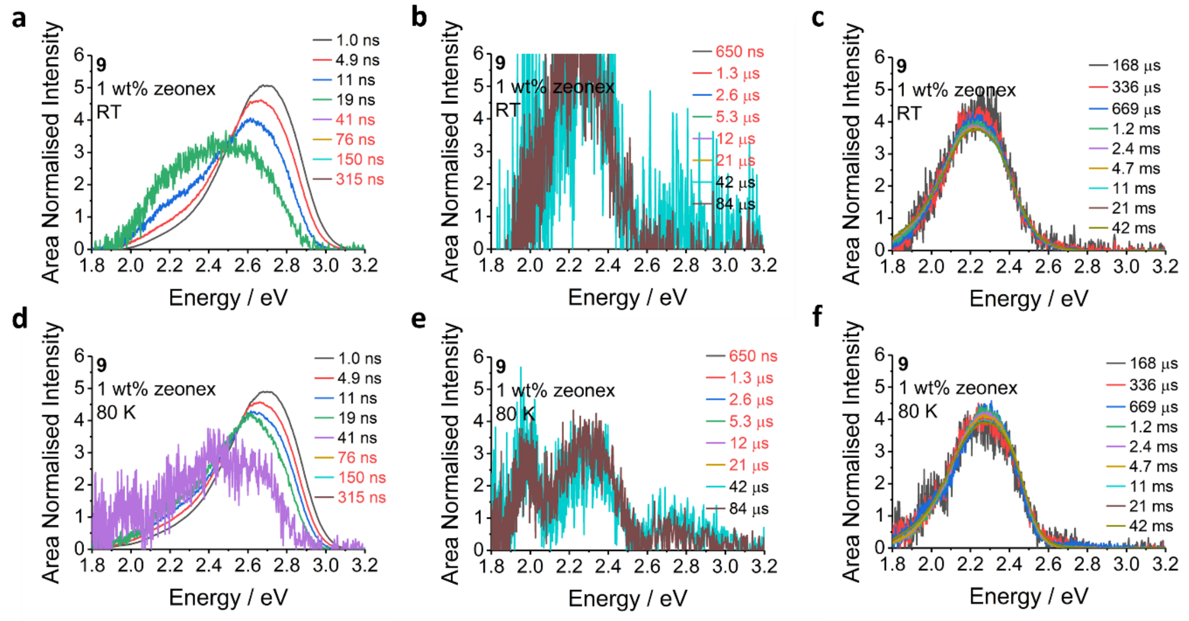

Figure S7.13 Time resolved photoluminescence spectra of **9** at 1 wt% in zeonex. Measured at (a-c) room temperature and (d-f) 80 K.

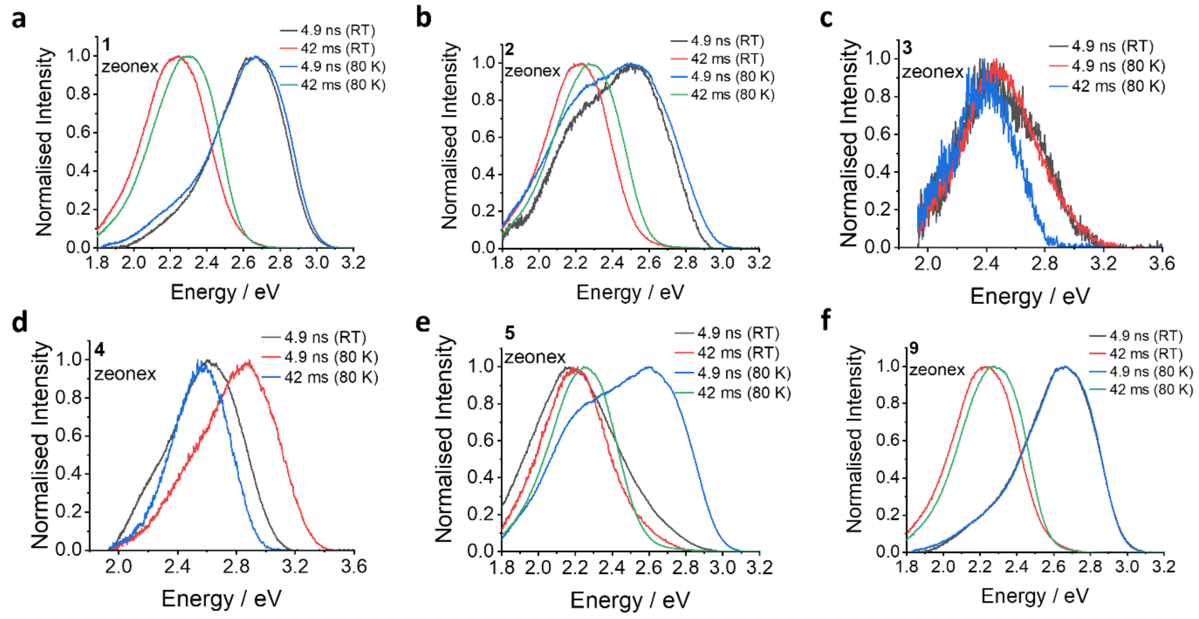

Figure S7.14 Time resolved photoluminescence spectra at prompt (4.9 ns) and delayed (42 ms) time regimes for a) **1** 1 wt% zeonex at RT and 80 K, b) **2** 1 wt% zeonex at RT and 80K, c) **3** 1 wt% zeonex at RT and 0.1 wt% zeonex at 80 K, d) **4** 1 wt% zeonex at RT and 0.1 wt% zeonex at 80 K, e) **5** 1 wt% zeonex at RT and 80 K and f) **9**1 wt% in zeonex at RT and 80 K.

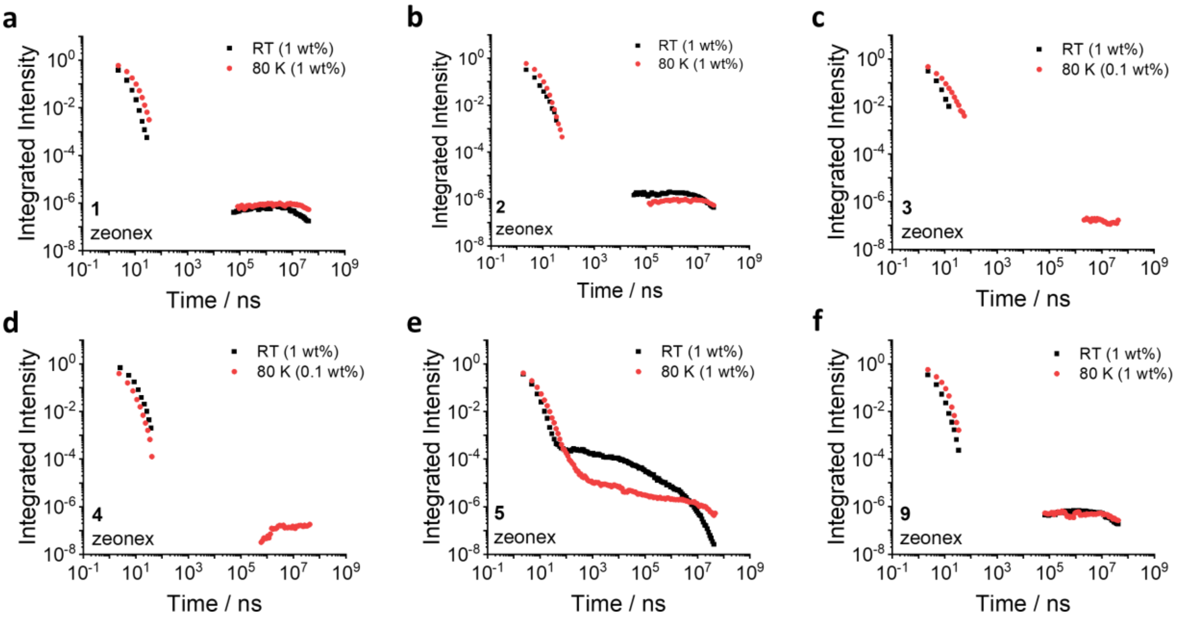

Figure S7.15 Integrated decay curves of compound a) **1**, b) **2**, c) **3**, d) **4**, e) **5** and f) **9** in zeonex film at RT and 80 K.

8. References

[1] C. J. O’Brien, E. A. B. Kantchev, C. Valente, N. Hadei, G. A. Chass, A. Lough, A. C. Hopkinson, M. G. Organ, *Chem. - A Eur. J.* **2006**, *12*, 4743–4748.

[2] C. Rothe, A. P. Monkman, *Phys. Rev. B - Condens. Matter Mater. Phys.* **2003**, *68*, 075208.

[3] L. Krause, R. Herbst-Irmer, G. M. Sheldrick, D. Stalke, *J. Appl. Crystallogr.* **2015**, *48*, 3–10.

[4] G. M. Sheldrick, *Acta Crystallogr. Sect. A Found. Crystallogr.* **2008**, *64*, 112–122.

[5] G. M. Sheldrick, *Acta Crystallogr. Sect. C Struct. Chem.* **2015**, *71*, 3–8.

[6] O. V. Dolomanov, L. J. Bourhis, R. J. Gildea, J. A. K. Howard, H. Puschmann, *J. Appl. Crystallogr.* **2009**, *42*, 339–341.
